# Predicting the Proteins of *Angomonas deanei*, *Strigomonas culicis* and Their Respective Endosymbionts Reveals New Aspects of the Trypanosomatidae Family

**DOI:** 10.1371/journal.pone.0060209

**Published:** 2013-04-03

**Authors:** Maria Cristina Machado Motta, Allan Cezar de Azevedo Martins, Silvana Sant’Anna de Souza, Carolina Moura Costa Catta-Preta, Rosane Silva, Cecilia Coimbra Klein, Luiz Gonzaga Paula de Almeida, Oberdan de Lima Cunha, Luciane Prioli Ciapina, Marcelo Brocchi, Ana Cristina Colabardini, Bruna de Araujo Lima, Carlos Renato Machado, Célia Maria de Almeida Soares, Christian Macagnan Probst, Claudia Beatriz Afonso de Menezes, Claudia Elizabeth Thompson, Daniella Castanheira Bartholomeu, Daniela Fiori Gradia, Daniela Parada Pavoni, Edmundo C. Grisard, Fabiana Fantinatti-Garboggini, Fabricio Klerynton Marchini, Gabriela Flávia Rodrigues-Luiz, Glauber Wagner, Gustavo Henrique Goldman, Juliana Lopes Rangel Fietto, Maria Carolina Elias, Maria Helena S. Goldman, Marie-France Sagot, Maristela Pereira, Patrícia H. Stoco, Rondon Pessoa de Mendonça-Neto, Santuza Maria Ribeiro Teixeira, Talles Eduardo Ferreira Maciel, Tiago Antônio de Oliveira Mendes, Turán P. Ürményi, Wanderley de Souza, Sergio Schenkman, Ana Tereza Ribeiro de Vasconcelos

**Affiliations:** 1 Laboratório de Ultraestrutura Celular Hertha Meyer, Instituto de Biofísica Carlos Chagas Filho, Universidade Federal do Rio de Janeiro, Rio de Janeiro, Rio de Janeiro, Brazil; 2 Laboratório de Metabolismo Macromolecular Firmino Torres de Castro, Instituto de Biofísica Carlos Chagas Filho, Universidade Federal do Rio de Janeiro, Rio de Janeiro, Rio de Janeiro, Brazil; 3 Laboratório Nacional de Computação Científica, Laboratório de Bioinformática, Petrópolis, Rio de Janeiro, Brazil; 4 BAMBOO Team, INRIA Grenoble-Rhône-Alpes, Villeurbanne, France; 5 Laboratoire de Biométrie et Biologie Evolutive, Université de Lyon, Université Lyon 1, CNRS, UMR5558, Villeurbanne, France; 6 Departamento de Genética, Evolução e Bioagentes, Instituto de Biologia, Universidade Estadual de Campinas, Campinas, São Paulo, Brazil; 7 Departamento de Ciências Farmacêuticas, Faculdade de Ciências Farmacêuticas de Ribeirão Preto, Universidade de São Paulo, Ribeirão Preto, São Paulo, Brazil; 8 Laboratório Nacional de Ciência e Tecnologia do Bioetanol, Campinas, São Paulo, Brazil; 9 Departamento de Bioquímica e Imunologia, Instituto de Ciências Biológicas, Universidade Federal de Minas Gerais, Belo Horizonte, Minas Gerais, Brazil; 10 Laboratório de Biologia Molecular, Instituto de Ciências Biológicas, Universidade Federal de Goiás, Goiânia, Goiás, Brazil; 11 Laboratório de Biologia Molecular de Tripanossomatídeos, Instituto Carlos Chagas/Fundação Oswaldo Cruz, Curitiba, Paraná, Brazil; 12 Laboratório de Genômica Funcional, Instituto Carlos Chagas/Fundação Oswaldo Cruz, Curitiba, Paraná, Brazil; 13 Centro Pluridisciplinar de Pesquisas Químicas, Biológicas e Agrícolas, Universidade Estadual de Campinas, Campinas, São Paulo, Brazil; 14 Departamento de Parasitologia, Instituto de Ciências Biológicas, Universidade Federal de Minas Gerais, Belo Horizonte, Minas Gerais, Brazil; 15 Laboratórios de Protozoologia e de Bioinformática, Departamento de Microbiologia, Imunologia e Parasitologia, Centro de Ciências Biológicas, Universidade Federal de Santa Catarina, Florianópolis, Santa Catarina, Brazil; 16 Departamento de Bioquímica e Biologia Molecular, Centro de Ciências Biológicas e da Saúde, Universidade Federal de Viçosa, Viçosa, Minas Gerais, Brazil; 17 Laboratório Especial de Ciclo Celular, Instituto Butantan, São Paulo, São Paulo, Brazil; 18 Departamento de Biologia, Faculdade de Filosofia, Ciências e Letras de Ribeirão Preto, Universidade de São Paulo, Ribeirão Preto, São Paulo, Brazil; 19 Departamento de Microbiologia, Imunologia e Parasitologia, Escola Paulista de Medicina, Universidade Federal de São Paulo, São Paulo, São Paulo, Brazil; Hospital for Sick Children, Canada

## Abstract

Endosymbiont-bearing trypanosomatids have been considered excellent models for the study of cell evolution because the host protozoan co-evolves with an intracellular bacterium in a mutualistic relationship. Such protozoa inhabit a single invertebrate host during their entire life cycle and exhibit special characteristics that group them in a particular phylogenetic cluster of the Trypanosomatidae family, thus classified as monoxenics. In an effort to better understand such symbiotic association, we used DNA pyrosequencing and a reference-guided assembly to generate reads that predicted 16,960 and 12,162 open reading frames (ORFs) in two symbiont-bearing trypanosomatids, *Angomonas deanei* (previously named as *Crithidia deanei*) and *Strigomonas culicis* (first known as *Blastocrithidia culicis*), respectively. Identification of each ORF was based primarily on TriTrypDB using tblastn, and each ORF was confirmed by employing getorf from EMBOSS and Newbler 2.6 when necessary. The monoxenic organisms revealed conserved housekeeping functions when compared to other trypanosomatids, especially compared with *Leishmania major*. However, major differences were found in ORFs corresponding to the cytoskeleton, the kinetoplast, and the paraflagellar structure. The monoxenic organisms also contain a large number of genes for cytosolic calpain-like and surface gp63 metalloproteases and a reduced number of compartmentalized cysteine proteases in comparison to other TriTryp organisms, reflecting adaptations to the presence of the symbiont. The assembled bacterial endosymbiont sequences exhibit a high A+T content with a total of 787 and 769 ORFs for the *Angomonas deanei* and *Strigomonas culicis* endosymbionts, respectively, and indicate that these organisms hold a common ancestor related to the Alcaligenaceae family. Importantly, both symbionts contain enzymes that complement essential host cell biosynthetic pathways, such as those for amino acid, lipid and purine/pyrimidine metabolism. These findings increase our understanding of the intricate symbiotic relationship between the bacterium and the trypanosomatid host and provide clues to better understand eukaryotic cell evolution.

## Introduction

Protists of the Trypanosomatidae family have been intensively studied because some of them are agents of human illnesses such as Chagas’ disease, African sleeping sickness, and leishmaniasis, which have a high incidence in Latin America, Sub-Saharan Africa, and parts of Asia and Europe, together affecting approximately 33 million people. Some species are also important in veterinary medicine, seriously affecting animals of economic interest such as horses and cattle. In addition, some members of the *Phytomonas* genus infect and kill plants of considerable economical interest such as coconut, oil palm, and cassava. These organisms circulate between invertebrate and vertebrate or plant hosts. In contrast, monoxenic species, which predominate in this family, inhabit a single invertebrate host during their entire life cycle [1].

Among the trypanosomatids, six species found in insects bear a single obligate intracellular bacterium in their cytoplasm [2], with *Angomonas deanei* and *Strigomonas culicis* (previously named as *Crithidia deanei* and *Blastocrithidia culicis*, respectively) representing the species better characterized by ultrastructural and biochemical approaches [3]. In this obligatory association, the endosymbiont is unable to survive and replicate once isolated from the host, whereas aposymbiotic protozoa are unable to colonize insects [4], [5]. The symbiont is surrounded by two membrane units and presents a reduced peptidoglycan layer, which is essential for cell division and morphological maintenance [6]. The lack of a typical gram-negative cell wall could facilitate the intense metabolic exchange between the host cell and the symbiotic bacterium.

Biochemical studies revealed that the endosymbiont contains enzymes that complete essential metabolic pathways of the host protozoan for amino acid production and heme biosynthesis, such as the enzymes of the urea cycle that are absent in the protozoan [7], [8], [9], [10], [11]. Furthermore, the bacterium enhances the formation of polyamines, which results in high rates of cell proliferation in endosymbiont-bearing trypanosomatids compared to other species of the family [12]. Conversely, the host cell supplies phosphatidylcholine, which composes the endosymbiont envelope [5], and ATP produced through the activity of protozoan glycosomes [13].

The synchrony in cellular division is another striking feature of this symbiotic relationship. The bacterium divides in coordination with the host cell structures, especially the nucleus, with each daughter cell carrying only one symbiont [14]. The presence of the prokaryote causes ultrastructural alterations in the host trypanosomatid, which exhibits a reduced paraflagellar structure and a typical kinetoplast DNA network [15], [16], [17]. The endosymbiont-harboring strains exhibit a differential surface charge and carbohydrate composition than the aposymbiotic cells obtained after antibiotic treatment [18], [19]. Furthermore, the presence of the symbiotic bacterium influences the protozoan interaction with the insect host, which seems to be mediated by gp63 proteases, sialomolecules, and mannose-rich glycoconjugates [20], [21].

Molecular data support the grouping of all endosymbiont-containing trypanosomatids together in a single phylogenetic branch. Moreover, studies based on rRNA sequencing suggest that symbionts from different protozoan species share high identities and are most likely derived from an ancestor of a β-proteobacterium of the genus *Bordetella*, which belongs to the Alcaligenaceae family [2], [22], [23]. Taken together, these results suggest that a single evolutionary event gave rise to all endosymbiont-bearing trypanosomatids, recapitulating the process that led to the formation of the mitochondrion in eukaryotic cells [24].

In this work, we analyzed the predicted protein sequences of *A. deanei* and *S. culicis* and their respective symbionts. This is the first time that genome databases have been generated from endosymbiont-containing trypanosomatids, which represent an excellent biological model to study eukaryotic cell evolution and the bacterial origin of organelles. The analysis presented here also clarifies aspects of the evolutionary history of the Trypanosomatidae family and helps us to understand how these protozoa maintain a close symbiotic relationship.

## Materials and Methods

Materials and methods are described in the Text S1.

### Nucleotide Sequence Accession Numbers

The sequences of *Angomonas deanei*, *Strigomonas culicis*, *Candidatus* Kinetoplastibacterium crithidii and *Candidatus* Kinetoplastibacterium blastocrithidii were assigned as PRJNA169008, PRJNA170971, CP003978 and CP003733, respectively, in the DDBJ/EMBL/GenBank.

## Results and Discussion

### General Characteristics

A 454-based pyrosequencing generated a total of 3,624,411 reads with an average length of 365 bp for *A. deanei* and a total of 2,666,239 reads with an average length of 379 bp for *S. culicis* (Table 1). A total of 16,957 and 12,157 ORFs were obtained for *A. deanei* and *S. culicis* genomes using this strategy, while their respective endosymbionts held a total of 787 and 769 ORFs, respectively. The total number of ORFs includes non-coding protein tRNA and rRNA genes. Tables 1 and 2 present the number of known proteins, hypothetical and partial ORFs for the two trypanosomatids and their endosymbionts, respectively.

**Table 1:** Protein Reference Sequence-Guided Assembly data of *A. deanei* and *S. culicis* genomes.

Parameter	*A. deanei*	*S. culicis*
Reads	3,624,411	2,666,239
Average reads length (bp)	365	379
Steps	3	5
Genes in contigs (protein reference sequence)	12,469	9,902
Genes in exclusive contigs	4,435	2,202
Number of known protein ORFs	7,912	6,192
Number of hypothetical ORFs	8,791	5,700
Number of partial ORFs	206	217
Total number of genes (including tRNAs and rRNAs)	16,957	12,157

**Table 2:** General characteristics of the *A. deanei* and *S. culicis* symbionts.

Parameter	*A. deanei* symbiont	*S. culicis* symbiont
Length (BP)	821,813	820,037
G+C (%)	30.96%	32.55%
Number of known protein CDSs	640	637
Number of hypothetical CDSs	94	78
Coding region (% of genome size)	88	87
Average CDSs length (bp)	987 bp	1,004 bp
rRNA	9	9
rRNA 16 s	3	3
rRNA 23 s	3	3
rRNA 5 s	3	3
tRNA	44	45
Total number of genes	787	769

The tRNA genes representing all 20 amino acids were identified in both trypanosomatids and their respective symbionts. At least one copy of the rRNA genes (18S, 5.8S and 28S) was identified in the genomes of *A. deanei* and *S. culicis*. We found that bacterial endosymbiont genomes also contain at least three copies of the rRNA operon.

### General Protein Cluster Analysis

**Figure 1:**
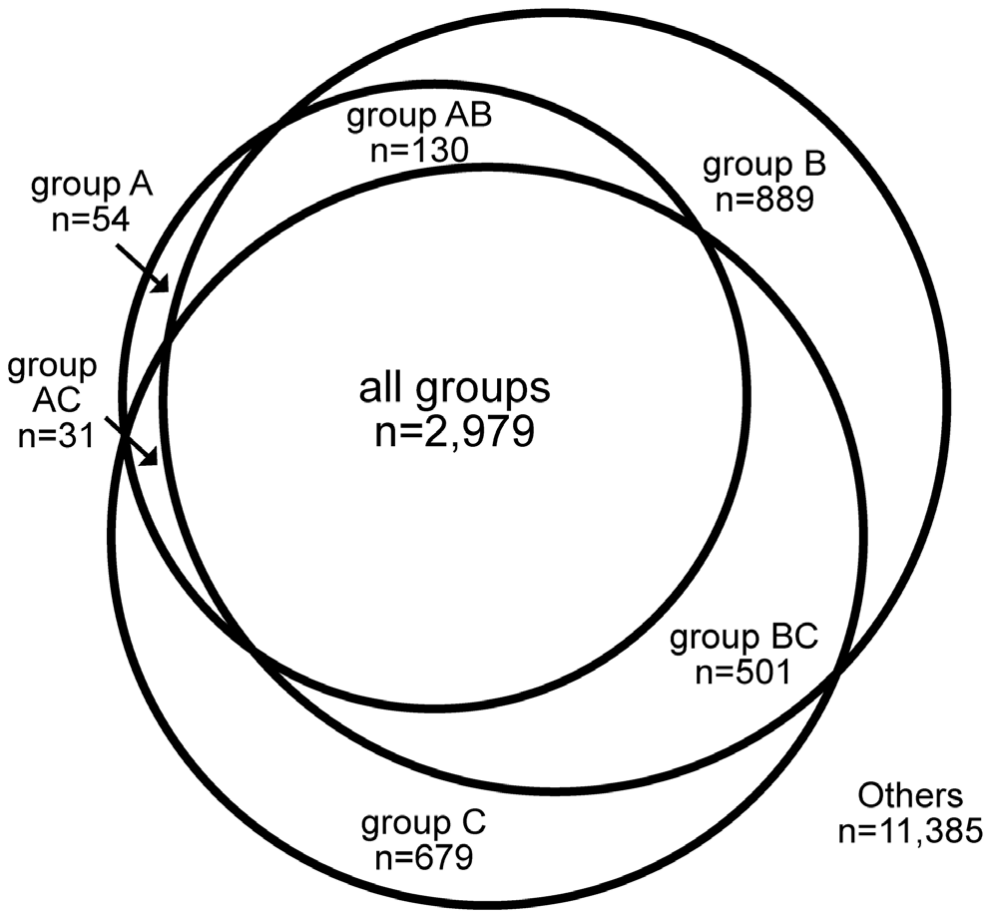
Venn diagram illustrating the distribution of MCL protein clusters. The diagram shows the cluster distribution comparing endosymbiont-bearing trypanosomatids (group A), *Leishmania* sp. (group B) and *Trypanosoma* sp. (group C). Protein clusters with less clear phylogenetic distributions are identified as others.

A total of 16,648 clusters were identified. Of those, 2,616 (16.4%) contained proteins from all species analyzed. To provide a more comprehensive coverage of the phylogenetic distribution, we have separated the species into three groups: endosymbiont-bearing trypanosomatids (A, s = 2 species), *Leishmania* sp. (B, s = 5) and *Trypanosoma* sp. (C, s = 4), and we considered a protein cluster to be present in the group even if zero, two or one species were missing, respectively. The protein cluster distribution is shown in Figure 1.

In this way, 2,979 protein clusters (17.9%) were identified in all groups, with 130 (0.8%) identified only in groups A and B (AB group), 31 (0.2%) only in groups A and C (AC group), and 501 (3.2%) only in groups B and C (BC group). The AB group represents the proteins that are absent in the *Trypanosoma* sp. branch. These proteins are mainly related to general metabolic function (p = 46 proteins), hypothetical conserved (p = 37) or transmembrane/surface proteins (p = 33). The AC group is four-fold smaller than the AB group, in accordance with the closer relationship between endosymbiont-bearing trypanosomatids and *Leishmania* sp [25]. The proteins in the AC group are mainly related to general metabolic function (p = 11), transmembrane/surface proteins (p = 8) and hypothetical conserved proteins (p = 7), and the relative distribution between these categories is very similar to the distribution in the AB group. The BC group is almost four-fold larger than the AB group, and mainly consists of conserved hypothetical proteins. One hypothesis to explain these different levels of conservation could be that organisms from the genera *Trypanosoma* and *Leishmania* inhabit insect and mammalian hosts, while the symbiont-bearing protozoa are mainly insect parasites. Thus, different surface proteins would be involved in host/protozoa interactions and distinct metabolic proteins are required for survival in these diverse environments.

Only a small fraction of protein clusters (n = 54, 0.3%) was identified in group A. This finding is in striking contrast to protein clusters identified only in group B (n = 889, 5.3%) or only in group C (n = 679, 4.5%), which represent specializations of the *Leishmania* or *Trypanosoma* branches. This small set is mainly composed of hypothetical proteins without similar proteins in the GenBank database. Only three of the group A clusters are similar to bacterial proteins, with two of these similar to *Bordetella* (clusters 04518 and 05756). The third one is similar to the bacterial-type glycerol dehydrogenase of *Crithidia sp.* (cluster 07344).

Of all the clusters that are present in all species except for one (n = 1,274, 7.6%), 694 (54.5%) are missing in *S. culicis*, followed by *T. congolense* (n = 211, 16.6%), *A. deanei* (n = 201, 15.8%) and *T. vivax* (n = 104, 8.0%). The fact that endosymbiont-bearing species are better represented in these sets could be due to unidentified proteins in the assembly and/or cluster analysis. This is reinforced by the fact that among clusters containing proteins from just one species (n = 9,477; 56.9%), most (73.9%) are from species with genomes that are not completely assembled (*T. vivax,* n = 1,881, 19.8%; *T. congolense*, n = 1,845, 19.5%; *A. deanei,* n = 1,745, 18.4%; *S. culicis,* n = 1,530, 16.1%). *T. brucei* and *T. cruzi* also account for significant numbers of clusters with only a single species (n = 1,094, 11.5% and n = 1,071, 11.3%, respectively), and these clusters mainly consist of multigenic surface proteins.

Our data support the idea that endosymbiont*-*bearing trypanosomatids share a larger proportion of their genes with the *Leishmania* sp. in accordance with previous phylogenetic studies [2], [25]. Only one fifth of all trypanosomatid protein clusters are shared among most of the species analyzed here. This proportion increases to one fourth if we only analyze the *Leishmania* and *Trypanosoma* genera; however, the number of clusters specific for endosymbiont-bearing kinetoplastids is a relatively small proportion (0.6%) of all clusters, indicating that the specialization of genes in the species following this evolutionary process was relatively small.

### Genomic Characteristics of the *A. deanei* and *S. culicis* Endosymbionts

#### The endosymbiont genomes

Table 2 summarizes the genome analyses of both symbionts. The genome of the *A. deanei* endosymbiont contains 821,813 bp, with almost 31% G+C content and 787 CDSs. Of these, 640 (81.3%) were characterized as known CDSs, 94 (11.9%) as hypothetical, and 53 (6.7%) as rRNA or tRNA. The average CDS length is 987 bp, and coding regions account for 88% of the genome, indicating that the genome is highly compact. There are three copies of each rRNA and 44 tRNAs, suggesting a functional translation metabolism. The endosymbiont of *S. culicis* has a genome composed of 820,037 bps and 769 CDSs, 637 (83.5%) coding for known proteins, 78 (9.5%) annotated as hypothetical proteins, and 54 (6.0%) as rRNA or tRNA. The G+C content (32.6%) is similar to but slightly higher than that of the *A. deanei* endosymbiont (30.96%). *A. deanei* and *S. culicis* endosymbiont genomes are composed of 88 and 87% of CDSs with few regions formed by non-coding sequences.

**Figure 2:**
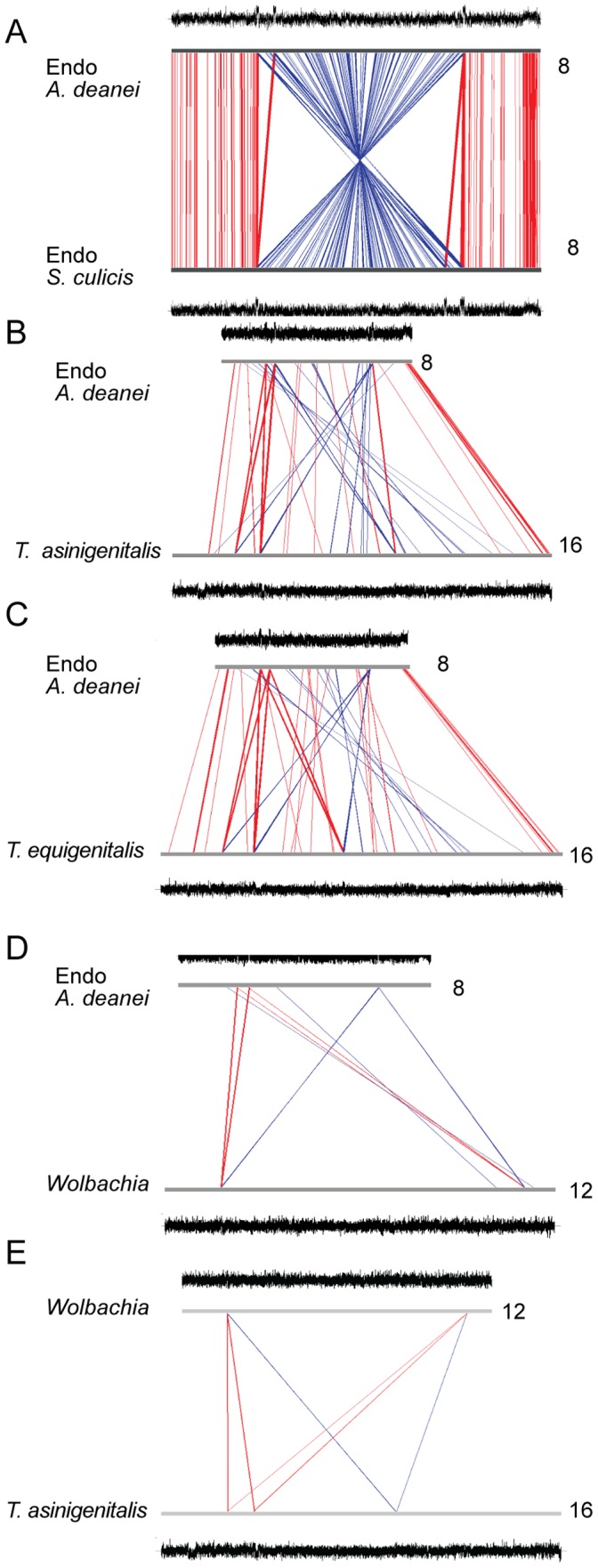
Genome alignments. The figure shows the alignment of the *A. deanei* endosymbiont (Endo-*A. deanei*) and the *S. culicis* endosymbiont (Endo-*S. culicis*) (A); between Endo-*A. deanei* and *T. asinigenitalis* (B), *T. equigenitalis* (C), or *Wolbachia* (D); and between *Wolbachia* and *T. asinigenitalis* (E). Alignments were performed with the ACT program based on tblastx analyses. Red (direct similarity) and blue lines (indirect similarity) connect similar regions with at least 700 bp and a score cutoff of 700. The numbers on the right indicate the size of the entire sequence for each organism.

A direct comparison between the two endosymbionts indicated that they share 507 genes that meet the criteria for inclusion in a cluster as described in the Materials and Methods. This represents approximately 70% of the annotated genes in both genomes, indicating a certain degree of genetic similarity. Figure 2A shows the full alignment of the *A. deanei* and *S. culicis* symbionts. This alignment indicates the occurrence of an inversion involving approximately one half of the genomes. However, this inversion would be validated by experimental work. The observed differences agree with phylogenetic analyses suggesting the classification of these symbionts as different species, *Candidatus* Kinetoplastibacterium crithidii and *Candidatus* Kinetoplastibacterium blastocrithidii [2], [23].

#### The origins of symbionts in trypanosomatids

Previous phylogenetic studies based on sequencing of the small-subunit ribosomal DNA suggested that symbionts of trypanosomatids descended from a common ancestor, a β-proteobacteria of the *Bordetella* genus [2], [22], [23]. Comparisons of the endosymbiont genomes with the KEGG database revealed eight organisms that share high numbers of similar CDSs: *Bordetella petrii*, *A. xylosoxidans*, *Bordetella avium*, *Bordetella parapertussis*, *Pusillimonas*, *Bordetella bronchiseptica* and *Taylorella equigenitalis*. All these species are phylogenetically related to β-proteobacteria belonging to the Alcaligenaceae family. The genus *Taylorella* consists of two species, *T. equigenitalis* and *T. asinigenitalis*, which are microaerophilic, slow-growing gram-negative bacteria belonging to the family Alcaligenaceae [26], [27]. *T. equigenitalis* is an intracellular facultative pathogen in horses that causes contagious equine metritis (CEM), a sexually transmitted infection [28].

Based on these facts, clustering analysis was performed to compare these genomes and establish the genetic similarity among them. The clustering analysis compared the genomes of *A. deanei* and *S. culicis* endosymbionts, *T. equigenitalis* MCE9, *T. asinigenitalis* MCE3, *B. petrii* DSM 12804, *A. xylosoxidans* A8 and *Wolbachia pipiens* (WMel). For the *A. deanei* endosymbiont, the highest numbers of shared clusters are observed for *A. xylosoxidans* (490 clusters) and *B. petrii* (483 clusters), followed by *T. asinigenitalis* (376 clusters) and *T. equigenitalis* (375 clusters). However, considering the genome length, *T. equigenitalis* and *T. asinigenitalis* had the greater proportion of genes in clusters (24.1 and 24.67% of the annotated genes, respectively). The values for *A. xylosoxidans* and *B. petrii* are 7.59 and 9.61%, respectively. Note that the *A. xylosoxidans* plasmids pA81 and pA82 are not included in these comparisons. The *S. culicis* endosymbiont shares a high number of clusters (74%) with other genomes; considering 714 annotated genes (rRNA and tRNA genes were not taken into account), 544 (76.19%) were similar to genes of the other microorganisms. The highest number of clusters is shared between *A. xylosoxidans* (501 clusters) and *B. petrii* (495 clusters), followed by *T. asinigenitalis* (390) and *T. equigenitalis* (388 clusters). Using *W. pipiens* (wMel), an endosymbiont of *Drosophila melanogaster*, as an out-group, we found 70 clusters for *A. deanei* and 73 clusters for *S. culicis*. *Wolbachia* also shares a lower number of clusters with *T. asinigenitalis* (79) and *T. equigenitalis* (81).

*T. equigenitalis* MCE9 and *T. asinigenitalis* MCE3 contain 1,695,860 and 1,638,559 bps, respectively. Therefore, the *A. deanei* and *S. culicis* symbiont genomes are reduced when compared to *Taylorella,* which also have reduced genomes when compared to *Bordetella* or *Achromobacter* [26], [27]. Alignments indicate the existence of similar sequences between the *Taylorella* and the kinetoplastid symbionts (Figure 2B and C), corroborating the results obtained in the clustering analyses. Much less similarity is observed between *A. deanei* and *W. pipientis* wMel, as well as between *W. pipientis* and *T. asinigenitalis* using the same alignment parameters (Figure 2D and E). Both *Taylorella* genomes are AT-rich (37.4 and 38.3% for *T. equigenitalis* and *T. asinigenitalis*, respectively), a characteristic also shared with both symbionts. Therefore, it is possible that the process of adaptation to intracellular life involved substantial base-composition modification, as most symbiotic bacteria are AT-rich [29], [30].

The degree of similarity and even identity of the endosymbionts with *Taylorella* genomes and even with genomes of other species such as *Bordetella* and *Achromobacter* reinforce the origin of both endosymbionts from an ancestor of the Alcaligenaceae group. Both endosymbionts are similar to *T. equigenitalis*, *T. asinigenitalis*, *B. petrii,* and *A. xylosoxidans* and to other species of this family to different degrees. In absolute numbers, *B. petrii* and *A. xylosoxidans* have the highest numbers of clusters in common with the symbionts. However, considering the genome length, *Taylorella* species have the highest proportions of clusters in common with the *A. deanei* and *S. culicis* endosymbionts. A phylogenomic analysis using 235 orthologs was performed in order to establish the evolutionary history among *A. xylosoxidans* A8, *B. petrii* DSM 12804, *T. asinigenitalis* MCE3, *T. equigenitalis* MCE9, *Ca.* K. blastocrithidii and *Ca.* K. crithidii. The results indicated that symbionts present in both trypanosomatid species are closely related to the Alcaligenaceae family (Figure S1). *Pseudomonas aeruginosa* PA7 was the Gammaproteobacteria used as outgroup. These data corroborate the results from Alves et al. 2011 [11].

Although the genome lengths of both trypanosomatid bacteria are slightly larger than those of *Buchnera* sp. [31], they are several fold larger than those of symbiotic bacteria, which have extremely reduced genomes [32]. Analysis of the *B. pertussis* and *B. parapertussis* genomes revealed a process of gene loss during host adaptation [33], [34]. This process was proposed to be associated with mobile DNA elements such as Insertion Sequences (IS) and the presence of pseudo genes [33], [34]. However, the mechanism(s) involved in the length reduction observed for the genomes of the two symbionts studied here needs further investigation. Our data enable future studies examining the relationship between endosymbiosis in trypanosomatids and the origin of organelles in eukaryotic cells.

### Host Trypanosomatid Characteristics

#### The microtubule cytoskeleton and flagellum of the host trypanosomatids

The cytoskeleton is composed of structures such as the microtubular subpelicular corset, the axoneme, the basal body, and the paraflagellar rod [35]. Thus, the cytoskeleton controls several characteristics of trypanosomatids such as their shape, the positions of structures, the flagellar beating and the host colonization. The presence of the symbiont has been related to unique characteristics of the host trypanosomatid.

Six members of the tubulin superfamily (α, β, δ, γ, ε and ζ) are present in *A. deanei* and *S. culicis.* Accordingly, δ and ε-tubulins are present in organisms that possess basal bodies and flagella [36]. γ-tubulin is localized in the basal body of *A. deanei* [14] as in other trypanosomatids [35]. Additionally, in common with other trypanosomatids, five centrins were identified in *A. deanei* and *S. culicis*. Furthermore, symbiont-containing trypanosomatids contain ε-tubulin, as in algae genomes, which can be related to the replication and inheritance of the centriole and basal bodies [37], [38]. Interestingly, the absence of microtubules that form the subpelicular corset in areas where the mitochondrion touches the plasma membrane is unique to symbiont-containing trypanosomatids [15]. However, we cannot explain this atypical microtubule distribution based on database searches. Moreover, no classical eukaryotic microtubule associated proteins (MAPs) or intermediate filament homologues were identified in symbiont-bearing or other trypanosomatids, except for TOG/MOR1 and Asp.

Actin and other protein homologues that play roles in the binding and nucleation of actin filaments are present in *A. deanei* and *S. culicis.* However, the ARP 2/3 complex, which is involved in the nucleation of actin, is absent in symbiont-bearing species. As actin seems to be necessary for endocytosis in trypanosomatids [39], the absence of some proteins involved in actin nucleation may be related to the low rates of endocytosis of these protozoa (unpublished data). Indeed, both symbiont-bearing trypanosomatids have low nutritional requirements, as the symbiotic bacterium completes essential metabolic routes of the host cell [3].

Trypanosomatids are the only organisms from the orders Euglenida and Kinetoplastida that have a paraflagellar rod. This structure is continuously associated with axoneme and it contains two major proteins designated PFR1 and PFR2 [35]. Importantly, only PFR1 was identified in *A. deanei* and *S. culicis*. Perhaps we missed PFR2 since these PFR proteins are highly repetitive and their assemblies are difficult. Nevertheless, these species have a reduced paraflagellar rod located at the proximal area of the flagellum [15], [16], although the same pattern of flagellar beating described for other trypanosomatids is observed for *A. deanei* [40]. The paraflagellar rod components (PFC) 4, PFC 10, PFC 16, and PFC 18 were detected in the *A. deanei* database, whereas in *S. culicis* PFC 11 was also identified. Other minor components of the paraflagellar rod could not be detected. Accordingly, RNA interference (RNAi) knockdown of PFCs such as PFC3 does not impair the flagellar movement of *T. brucei* [41], differently from PFC4 and PFC6 depletion [42].

Several other minor flagellar proteins detected in these and other trypanosomatids are absent in *A. deanei* and *S. culicis*, especially the flagellar membrane proteins and those involved in intraflagellar transport (kinesins). Symbiont-containing species had adenylate kinase B (ADKB) but not ADKA, in contrast to other trypanosomatids, which express both. These proteins are involved in the maintenance of ATP supply to the distal portion of the flagellum [43], [44].

Taken together, the differences in the composition and function of the cytoskeleton in symbiont-containing trypanosomatids seem to represent adaptations to incorporate the endosymbiont. Further exploration of these differences could enable a better understanding of how endosymbiosis was established.

#### The kinetoplast

The kinetoplast is an enlarged portion of the single mitochondrion that contains the mitochondrial DNA, which exhibits an unusual arrangement of catenated circles that form a network. The kinetoplast shape and the kDNA topology vary according to species and developmental stage. Endosymbiont-containing trypanosomatids show differences in the morphology and topology of the kDNA network when compared to other species of the same family. Both species present a loose kDNA arrangement, but in *A. deanei*, the kinetoplast has a trapezoid-like shape with a characteristic transversal electron-dense band, whereas in *S. culicis* the disk shape structure is wider at the center in relation to the extremities [2], [17].

Differences in kDNA arrangement are related to low molecular weight basic proteins such as kinetoplast-associated protein (KAP), taking part in the organization and segregation of the kDNA network [45], [46]. Our data indicate that KAP4 and KAP3 homologues are present in *A. deanei*, while KAP4, KAP2 homologues, and ScKAP-like protein are found in *S. culicis* (Table S1). In addition, a conserved nine amino acid domain in the N-terminal region, most likely a mitochondrial import signal [47], [48], is found in AdKAP4 and ScKAP4 (amino acid positions 10 to 16) (Figure S2). Furthermore, ScKAP2 has a conserved domain called the High Mobility Group (HMG), indicating that this protein may be involved in protein-protein interactions. These KAPs might be related to the typical kDNA condensation of symbiont-bearing trypanosomatids.

#### Housekeeping genes

**Figure 3:**
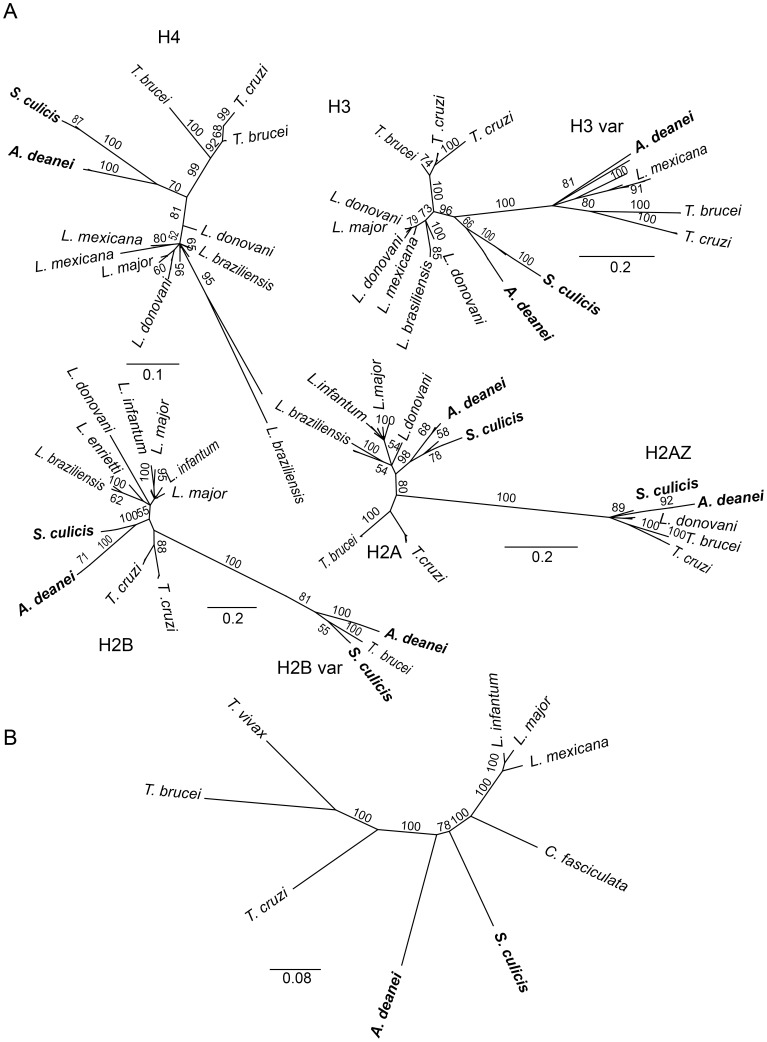
Phylogenetic of histones of *A. deanei, S. culicis,* and other trypanosomatids. Histone protein (panel A) and nucleotide (panel B) sequences were generated by MUSCLE tool using 10 iterations in the Geneious package [120]. Trees were constructed using the Geneious Tree Builder, by employing Jukes-Cantor genetic distance model with a neighbor-joining method and no out-groups. The consensus trees were generated from 100 bootstrap replicates of all detected histone genes, as shown below. Scale bars are indicated for each consensus tree. The trees in panel A are based in a collection of sequences of all trypanosomatids. The nucleotide sequences used for dihydrofolate reductase-thymidylate synthase are: *T. cruzi,* XM_810234; *T. brucei*, XM_841078; *T. vivax,* HE573023; *L. mexicana*, FR799559; *L. major*, XM_001680805; *L. infantum*, XM_001680805; and *C. fasciculata*, M22852.

Histones, which are responsible for structuring the chromatin, are highly conserved proteins that appeared in the eukaryotic branch of evolution. Although well conserved, Trypanosomatidae histones display differences in the N and C-terminal sequences, sites of post-translational modifications, when compared to other eukaryotes. Phylogenetic analysis revealed that histones and their variants in both *A. deanei* and *S. culicis* are clustered in a separate branch, between the *Trypanosoma* and *Leishmania* species (Figure 3A). Similar phylogenetic distribution is seen for the dihydrofolate reductase-thymidylate synthase when we performed the analysis using nucleotide sequences (Figure 3B). Nevertheless, the symbiont-bearing species show conservation in the sites of post-translation when compared to other trypanosomes as shown in supplementary Figure S3. In *A. deanei* and *S. culicis* the proteins related to the chromatin assembly are also maintained, including histones and histone-modifying enzymes as shown in Tables S2–S7 and Figure S4 of the supporting information. For a more detailed analysis about housekeeping genes of *A. deanei* and *S. culicis* see Text S1.

DNA replication, repair, transcription, translation and signal transduction in *A. deanei* and *S. culicis* functions can be respectively attributed at least to 914 ORFs and 643 ORFs (Table 3). Most of the genes are exclusive to the protozoan and are absent in the endosymbiont (Table 4), thus indicating that these processes are exclusive to the host organism as shown in the supplementary Tables S8–S13, typically containing a conserved spliced-leader RNA as found in other trypanosomes (see Figure S5 for more information). A total of 133 and 130 proteins with similar functions are detectable in the endosymbionts of both species, with up to 95% amino acid identity to proteins of *Bordetella* sp. and *A. xylosoxidans.*

**Table 3:** Numbers of ORFs identified in *A. deanei* and *S. culicis* and their symbionts, according to the mechanisms of DNA replication and repair, signal transduction, transcription and translation.

	Number of ORFs			
Mechanism	*A. deanei*	*S. culicis*	*A. deanei* symbiont	*S. culicis* symbiont
Replication and Repair	178	148	56	54
Base excision repair	34	34	9	9
DNA replication	54	32	11	11
Homologous recombination	11	11	16	15
Mismatch repair	28	29	12	12
Non-homologous end-joining	8	7	–	–
Nucleotide excision repair	43	35	8	7
Signal Transduction	136	46	1	1
Phosphatidylinositol signaling system	23	17	–	–
mTOR signaling pathway	113	29	–	–
Two component system	–	–	1	1
Transcription	96	61	3	3
Basal transcription factors	15	4	–	–
RNA polymerase	28	16	3	3
Spliceosome	53	41	–	–
Translation	504	388	73	72
Aminoacyl-tRNA biosynthesis	63	56	25	25
mRNA surveillance pathway	43	45	–	–
Ribosome proteins	231	152	48	47
Ribosome biogenesis in eukaryotes	84	66	–	–
RNA transport	83	69	–	–
TOTAL	914	643	133	130

**Table 4:** Summary of the origin of ORFs found in *A. deanei* and *S. culicis.*

	*A. deanei*		Symbiont
Functional Classification	Prokaryotes*	Eukaryotes**	P/E***
**Replication and Repair**			
Base excision repair	5	11	4/0
Nucleotide excision repair	2	16	9/0
Non-homologous end-joining	1	5	N
Mismatch repair	2	13	8/0
Homologous recombination	2	9	10/0
DNA replication	3	22	10/0
**Signal Transduction**			
Two-component system	N	N	1
Phosphatidylinositol signaling system	0	16	N
mTOR signaling pathway	0	8	N
MAPK signaling pahway - yeast	0	1	N
**Transcription**			
Spliceosome	0	20	N
RNA polymerase	0	16	3/0
Basal transcription factors	0	5	N
**Translation**			
RNA transport	0	31	N
Ribosome biogenesis in eukaryotes	0	27	N
Ribosome	0	75	48/0
mRNA surveillance pathway	0	17	N
Aminoacyl-tRNA biosynthesis	0	22	23

	*S. culicis*		Symbiont
Functional Classification	Prokaryotes	Eukaryotes	P/E
**Replication and Repair**			
Base excision repair	2	6	5/0
Nucleotide excision repair	2	10	7/0
Non-homologous end-joining	1	1	N
Mismatch repair	1	5	8/0
Homologous recombination	1	4	11/0
DNA replication	2	15	9/0
**Signal Transduction**			
Two-component system	N	N	1
Phosphatidylinositol signaling system	0	11	N
mTOR signaling pathway	0	8	N
MAPK signaling pathway - yeast	0	0	N
**Transcription**			
Spliceosome	0	13	
RNA polymerase	0	11	3/0
Basal transcription factors	0	2	
**Translation**			
RNA transport	0	19	N
Ribosome biogenesis in eukaryotes	0	20	N
Ribosome	0	53	46/0
mRNA surveillance pathway	0	16	N
Aminoacyl-tRNA biosynthesis	0	18	23

* Number of genes with identity to Prokaryotes.

** Number of genes with identity to Eukaryotes.

*** Ratio of the number of genes with identity to Prokaryotes/Eukaryotes.

**Figure 4:**
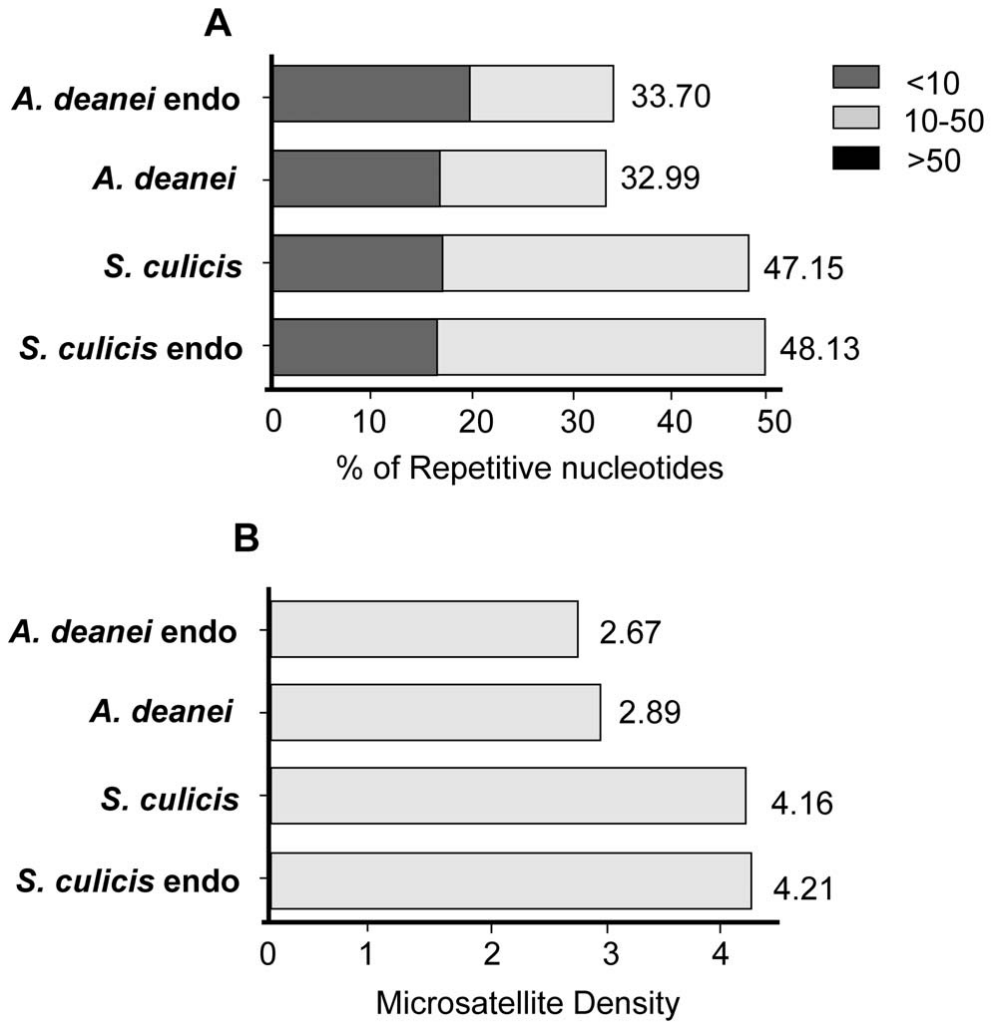
Microsatellite content in the genomes of *A. deanei*, *S. culicis,* and their endosymbionts. Panel (A) shows the percentage of repetitive nucleotides for each repeat length. The total numbers of nucleotides are derived from microsatellite sequences divided by the total number of assembled nucleotides. Panel (B) shows the microsatellite density. The values indicate the number of microsatellite *loci* divided by the genome length×100.

Similar DNA repair proteins are present in both eukaryote and prokaryote predicted sequences. These findings demonstrate that the endosymbionts conserved essential housekeeping proteins despite their genome reduction. Some differences were found in mismatch repair (MMR) between symbiont-bearing trypanosomatid genomes. As microsatellite instability is considered the molecular fingerprint of the MMR system, we compared the abundance of tandem repeats in the genomes of *A. deanei* and *S. culicis* and their respective endosymbionts. We noticed that the genomes of *S. culicis* and its endosymbiont are more repetitive than the genomes of *A. deanei* and its endosymbiont (Figure 4A). However, the higher repetitive content of the genomes of *S. culicis* and its endosymbiont is not only due to the higher number of microsatellite loci (Figure 4B) but also to the expansion of the size of the microsatellite sequences. These data suggest that microsatellites of *S. culicis* and its endosymbiont evolved faster than those of *A. deanei* and its endosymbiont. Interestingly, we identified some missing components of the MMR machinery in *S. culicis* that are present in *A. deanei*, such as exonuclease I (Exo I), a 5′-3′ exonuclease that is implicated in the excision step of the DNA mismatch repair pathway (Table S9). Several studies have correlated the silencing of the ExoI protein and/or mutations of the ExoI gene and microsatellite instability with development of lymphomas and colorectal cancer [49], [50], [51]. Therefore, we speculate that deficiencies in the MMR machinery in *S. culicis* may be related to the high proportion of microsatellites in its genome. The association between microsatellite instability and MMR deficiency has already been described for *T. cruzi* strains [52], [53]. The same variability pattern is observed for each symbiont, despite the fact that the MMR machinery seems to be complete in both symbiotic bacteria (Table S10). It is tempting to speculate that this finding may indicate that the parasite and its endosymbiont are exposed to the same environment and therefore may be subjected to similar selective pressures imposed by an external oxidative condition.

*A. deanei* and *S. culicis* have 607 and 421 putative kinase-encoding genes, respectively (Table 5). Thirty one of the *A. deanei* kinases were classified in the AGC family, 31 as atypical, 49 as CAMK, 15 as CK1, 108 as CMGC, 64 as STE, 1 as TKL, 81 as others, and 227 that could not be classified in any of these families. No typical tyrosine kinases (TK) are present in *A. deanei* or *S. culicis*, as in other trypanosomes, although tyrosine residues are subjected to phosphorylation [54], [55]. Several phosphatases have also been described in trypanosomes, pointing toward their regulatory role in the development of these organisms. The *T. brucei* PTP (*Tb*PTP1) is associated with the cytoskeleton and has been reported to be intrinsically involved in this parasite’s cycle [56]. Similar sequences are found in the *A. deanei* genome, including PTP1, which is not found in the *S. culicis* database. Additionally, a large number of other PTPs appear in both genomes, including ectophosphatases (Table S14).

**Table 5:** Kinase families identified in trypanosomatids.

Kinase family	*A. deanei*	*S. culicis*
AGC	31	23
Atypical	31	21
CAMK	49	39
CK1	15	8
CMGC	108	77
STE	64	31
TKL	1	0
Other	81	58
No hits found	227	164
TOTAL	607	421

Two major signal transduction pathways are described in trypanosomatids: one is the cyclic AMP-dependent route and the other is the mitogen-activated protein kinase pathway [57]. The major components of these pathways, including phosphatidylinositol signaling, mTOR and MAPK signaling pathways are identified in *A. deanei* and *S. culicis*. These pathways may regulate cellular activities such as gene expression, mitosis, differentiation, and cell survival/apoptosis (Table 6).

**Table 6:** Representative ORFs involved in the signal transduction pathways in *A. deanei* and *S. culicis*.

Product	*A. deanei*	*S. culicis*
Calmodulin	AGDE02036	STCU01612
Diacylglycerol kinase	AGDE02361	STCU00226
CDP-diacylglycerol-inositol-3-phosphatidyltransferase	AGDE04835	STCU01286
Myo-inositol-1(or 4) monophosphatase	AGDE08470	STCU02993
Phospholipase C	AGDE12052	STCU02439
Phosphatidylinositol 4-phosphate 5-kinase alpha	AGDE09669	STCU03909
Inositol-1,4,5-trisphosphate (IP3) 5-phosphatase	AGDE06690	nd
phosphatidate cytidylyltransferase	AGDE09922	nd
Mitogen-activated protein kinase 5	AGDE00259	STCU00603
Protein kinase A	AGDE06073	STCU01525
TP53 regulating kinase	AGDE08400	nd
Serine/threonine-protein kinase CTR1	AGDE00613	nd
Casein kinase	AGDE11868	STCU01611
Phosphoinositide-specific phospholipase C	nd	STCU09903

nd: not determined.

**Figure 5:**
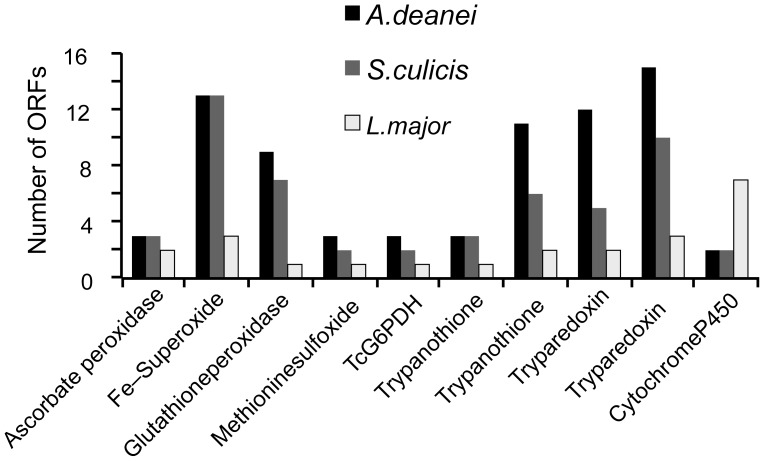
Oxidative stress-related genes in the genomes of *A. deanei*, *S. culicis* and *L. major*. The figure shows the number of ORFs for the indicated enzymes for each species.

Most genes encoding heat shock proteins are present in symbiont-bearing species, as was previously described in other trypanosomatids (Table S15). Genes for redox molecules and antioxidant enzymes, which are part of the oxidative stress response, are also present in the *A. deanei* and *S. culicis* genomes. Both contain slightly more copies of ascorbate peroxidase, methionine sulfoxide reductase, glucose-6-phosphate dehydrogenase, and trypanothione reductase genes than *L. major*. In particular, several genes related to the oxidative stress response are present in higher copy numbers in symbiont-bearing trypanosomatids than in *L. major* (Figure 5).

*A. deanei* sequences codify enzymes involved in RNAi, a mechanism described in various organisms that promotes the specific degradation of mRNA. RNAi is initiated by the recognition of double-stranded RNA through the action of endoribonucleases known as Dicer and Slicer, members of the Argonaut (Ago) protein family (RNase H-type) [58]. The cleavage of double-stranded RNA results in a complex that specifically cleaves mRNA molecules that are homologous to the double-stranded sequence. *A. deanei* contains the gene coding Dicer-like protein II (AGDE14022) and Ago1 (AGDE11548), homologous to enzymes in *T. brucei* and *Leishmania braziliensis* (Ngo *et al.,* 1998; Lye *et al.,* 2010). In addition, *A. deanei* contains the RNA interference factor (RIF) 4 (AGDE09645) with an exonuclease domain of the DnaQ superfamily, as described in *T. brucei.* A fragmented RIF5 sequence was also found in the sequence AGDE15656. These proteins were shown to interact with Ago1 as was recently demonstrated in *T. brucei* [59], suggesting that RNAi might be active in *A. deanei*. None of these sequences were found in the *S. culicis* database.

### The Coordinated Division of the Bacterium during the Host Protozoan Cell Cycle

#### Cell cycle control in host trypanosomes

In eukaryotes, DNA replication is coordinated with cell division by a cyclin-CDK complex that triggers DNA duplication during the S phase of the cell cycle. Multiple copies of the CRK gene (cdc2-related protein kinase) are found in *A. deanei* and four genes coding for two different CRKs are present in *S. culicis*. Both proteins exhibit structural features of the kinase subunits that make up the CDK complex, as they contain the cyclin-binding PSTAIRE motif, an ATP-binding domain and a catalytic domain. These motifs and domains are not the same in different CRKs (Figure S6), strongly suggesting that these CRKs might control different stages of the cell cycle. *A. deanei* contains four genes coding for cyclins. Three of these genes are homologues to mitotic cyclin from *S. cerevisiae* and *T. brucei*. However, none of them contain the typical destruction domain present in *T. brucei* mitotic cyclin [60]. The fourth codes for a *S. cerevisiae* Clb5 homolog, an S-phase cyclin. These data indicate that more than one CRK and more than one cyclin would be involved in the cell cycle control of symbiont-containing trypanosomatids, suggesting that tight regulation must occur to guarantee the precise maintenance of only one symbiont per cell [14].

#### Cell cycle control in the endosymbionts

**Figure 6:**
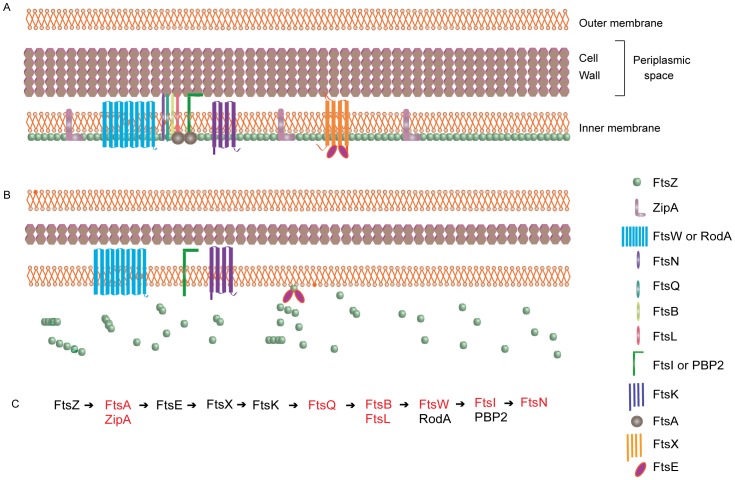
Schematic representation of the cell division machinery found in the endosymbionts. Panel (A) indicates the basic model derived from a gram-negative bacterium with the localization of each component (shown on the right). Panel (B) represents the components found in the endosymbiont of *A. deanei*, and Panel (C) shows the steps in the assembly of the Z-ring. The missing components of the *A. deanei* endosymbiont are drawn in red.

Bacterial cell division is a highly regulated event that mainly depends on two structures, the peptidoglycan layer and the Z ring. The first step in the segregation of the bacterium is the formation of a polymerized Z ring at the middle of the cell. This structure acts as a platform for the recruitment of other essential proteins named Filament Temperature Sensitive (Fts), which are mainly involved in the formation and stabilization of the Z ring [61], [62] and in establishing the peptidoglycan septum formation site in most bacteria [63] (Figure 6A).

Two *fts* sequences were identified in *A. deanei* and *S. culicis* symbionts based on *Bordetella* genes (Table 7). One of them is FtsZ, which requires integral membrane proteins such as Zip A and FtsA for anchoring. However, these sequences are absent in the symbionts. FtsZ should also interact with FtsE, which is absent in both symbionts. This protein is homologous to the ATP-binding cassette of ABC transporters and co-localizes with the division septum [64]. The lack of these proteins could be related to the absence of a classical Z ring in these symbionts. The other sequence is FtsK that docks FtsQ, FtsB and FtsL, which are related to the formation of the peptidoglycan layer in *E. coli* and *B. subtilis* [65], [66], [67], but these proteins are absent in symbionts, as in most bacteria that exhibit reduced peptidoglycan production [64]. RodA, a homologous integral membrane protein involved in bacterial cell growth, is detected in the endosymbionts. RodA could replace FtsW, which is absent in both symbionts. FtsW is essential for the localization of FtsI (PBP3) in the Z ring [68], which is absent in the symbiotic bacteria.

**Table 7:** Members of the Fts family and PBPs that are present in endosymbionts of *A. deanei* and *S. culicis*.

Function	Protein	*A. deanei*	*S. culicis*
Stabilization and attachment of FtsZ polymers to the inner membrane	FtsA	nd	nd
	FtsE	nd	nd
	ZipA	nd	nd
	FtsK	CKCE00084	CKBE00632
Interaction with peptidogycan synthases PBPs	FtsQ	nd	nd
	FtsB	Nd	nd
	FtsL	nd	nd
	FtsN	nd	nd
Lipid II flippase	FtsW(RodA)	CKCE 00486	CKBE00079
Forms a dynamic cytoplasmic ring structure at midcell	FtsZ	CKCE00034	CKBE00683
Penicillin binding proteins (PBPs)	PBP1A	CKCE00524	CKBE00119
	PBP2	CKCE00487	CKBE00080
	FtsI/PBP3	CKCE00487	CKBE00080
	PBP4	nd	nd
	PBP5/dacC	CKCE00510	CKBE00105
	PBP6	nd	nd
	PBP6B	nd	nd
	PBP7	nd	nd
			

nd: not determined.

Endosymbionts have only one bifunctional synthase (PBP1A), while *E. coli* has PBP1A, PBP1B, and PBP1C. Cells require at least one of these synthases for viability. The peptidoglycan layer is functional in trypanosomatid symbionts, as shown by treatment with β-lactam antibiotics affecting the division of the bacterium, generating filamentous structures and culminating in cell lysis. PBP1 and PBP2 have also been detected at the symbiont envelope [6]. PBP1B interacts with the two essential division proteins, FtsN and PBP3/FtsI, which are absent in the symbiont. PBP1B can also interact with PBP2 that is identified in both symbiont databases (see Table 7).

A sequence encoding a minor PBP described in *E. coli* was also identified in the symbionts. This protein is known as a putative PBP precursor (PBP5/dacC). This PBP is involved in the regulation of the peptidoglycan structure, along with 3 other minor PBPs described in *E. coli*, but these are absent from the symbiont (Table 7). On the other hand, all the enzymes involved in the synthesis of activated nucleotide precursors for the assembly of the peptidoglycan layer are present in the symbiont genome, except for Braun’s lipoprotein (Lpp), which forms the lipid-anchored disaccharide-pentapeptide monomer subunit [69]. In *E. coli* strains, mutations in Lpp genes result in a significant reduction of the permeability barrier, although small effects on the maintenance of the cell growth and metabolism were observed in these cells [70], [71].

Taken together, we consider that gene loss in the *dcw* cluster [72] (represented in Figure 6) explains the lack of the FtsZ ring in the endosymbiont during its division process [73]. Moreover, the symbiont envelope contains a reduced peptidoglycan layer and lacks a septum during its division process, which can be related to the facilitation of metabolic exchanges, as well as to the control of division by the host protozoan [6]. These losses could be understood since the host trypanosomatid is controlling the number of symbiotic bacteria per cell. This phenomenon has been described for obligatory intracellular bacteria that co-evolve in eukaryotic cells, as well as for the organelles of prokaryotic origin, the chloroplast and the mitochondrion [74], [75].

### Metabolic Co-evolution of the Bacterium and the Host Trypanosomatid

Symbiosis in trypanosomatids is characterized as a mutual association where both partners benefit. These symbiont-bearing protozoa have low nutritional requirements, as intense metabolic exchanges occur. Our data corroborate previous biochemical and ultrastructural analyses showing that the bacterium has enzymes and metabolic precursors that complete important biosynthetic pathways of the host [76].

#### Oxidative phosphorylation

FoF1-ATP synthase and the entire mitochondrial electron transport chain are present in *A. deanei* and *S. culicis*, although some subunits are missing (Table 8). These species have a rotenone-insensitive NADH:ubiquinone oxidoredutase in complex I, as do other trypanosomatids [77]. Ten complex II (succinate:ubiquinone reductase) subunits of the twelve identified in *T. cruzi* [78] are also present in both trypanosomatids. Many subunits from complex III, composed of cytochrome c reductase, are found in *A. deanei* and *S. culicis*. In addition, these protozoa contain genes for cytochrome c, as previously suggested by biochemical studies in other symbiont-containing trypanosomatids [3], [79].

**Table 8:** Respiratory chain complexes identified in the predicted proteome of *A. deanei*, *S. culicis* and their respective endosymbionts.

	*A. deanei*	*A. deanei* endosymbiont	*S. culicis*	*S. culicis* endosymbiont
Complex I	33	0	33	0
Complex II	10	0	10	0
Complex III	5	0	4	0
Complex IV	10	2*	2	2*
Complex V	10	8	3	8

* The complex IV of the endosymbionts might be a cytochrome *d* ubiquinol oxidase identified in both organisms, instead a classical cytochrome *c* oxidase.

Both symbionts contain sequences with hits for all subunits of complex I, NADH:ubiquinone oxidoredutase, similar to *E. coli* (Table 8). Complexes II and III, including cytochrome *c*, and complex IV (cytochrome *c* oxidase, succinate:ubiquinone reductase and cytochrome c reductase, respectively) are not found in either symbiont. However, we detected the presence of cytochrome *d* as found in *Allochromatium vinosum*, and also a cytochrome *d* oxidase with a sequence close to that of *B. parapertussis*. All portions of the FoF1-ATP synthase were identified in symbionts, although not every subunit of each portion was found.

#### Lipid metabolism

The sphingophospholipid (SPL) content in *A. deanei* and its symbiont has been previously described, with phosphatidylcholine (PC) representing the major SPL in the host, whereas cardiolipin predominates in the symbiotic bacterium [5], [80]. The synthetic pathway of phosphatidylglycerol from glycerol phosphate is present in both host trypanosomatids (Table S16). The biosynthetic pathways of PC and PE from CDP-choline and CDP-ethanolamine (Kennedy pathways), that synthesize PC and PE respectively, are incomplete in *A. deanei* and *S. culicis.* Nevertheless, the methylation pathway (Greenberg pathway), which converts PE in PC, seems to be absent in both trypanosomatids, even though one enzyme sequence was identified in *A. deanei*.

The symbiont of *A. deanei* exhibits two routes for phosphatidylethanolamine (PE) synthesis, starting from CDP-diacylglycerol and producing phosphatidylserine as an intermediate (Table S17). Interestingly, this last step of the pathway is not found in the *S. culicis* endosymbiont. Importantly, both symbionts lack genes that encode proteins of PC biosynthetic pathways, reinforcing the idea that this phospholipid is mainly obtained from the host protozoa [5]. Remarkably, phoshpatidylglycerophosphatase A, which produces the intermediate phosphatidylglycerol necessary for cardiolipin biosynthesis, was not found in either protozoa but is present in both symbionts. As cardiolipin is present in the inner membranes of host mitochondria, the symbionts may complete cardiolipin biosynthesis.

Pathways for sphingolipid production, including the synthesis of ceramide from sphingosine-1P, are present in *A. deanei*, while *S. culicis* lacks enzymes of this pathway (Table S16). Both host trypanosomatids have glycerol kinase and 3-glycerophosphate acyltransferase, enzymes for the synthesis of 1,2-diacyl-sn-glycerol and triacylglycerol from D-glycerate. In endosymbionts, glycerolipid metabolism seems to be reduced to two enzymes: 3-glycerophosphate acyltransferase and 1-acylglycerol-3-phosphate O-acyltransferase (Table S17), suggesting metabolic complementation between partners.

Furthermore, both hosts contain enzymes of the biosynthesis pathway for ergosterol production from zymosterol, as well as the pathway of sterol biosynthesis that produces lanosterol from farnesyl-PP. These pathways are only complete in *A. deanei*. The symbionts do not have enzymes for sterol biosynthesis, in accordance with our previous biochemical analysis [80].

#### Metabolism of amino acids, vitamins, cofactors and hemin

**Figure 7:**
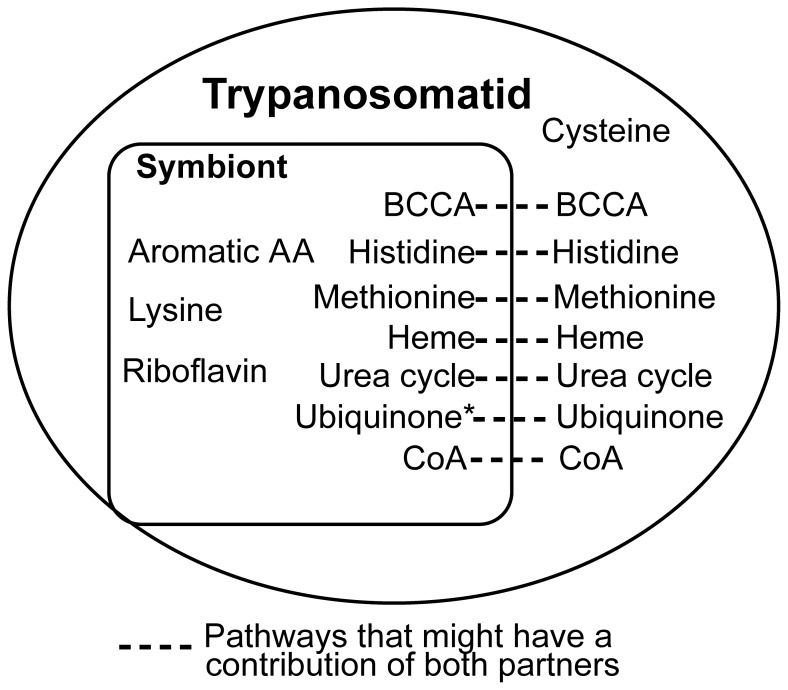
Main metabolic exchanges between host and endosymbionts. Schematic representation of the amino acids, vitamins, and cofactors exchanged between *A. deanei* and *S. culicis* and their respective symbionts. Dotted lines indicate pathways that have or might have contributions from both partners, whereas metabolites inside one of the circles, representing the symbiont or host, indicate that one partner holds candidate genes coding for enzymes of the whole biosynthetic pathway. *Candidate genes were only found for the symbiont of *S. culicis* and not for the symbiont of *A. deanei*. BCAA (branched-chain amino acids) are leucine, isoleucine and valine.

Symbiosis in trypanosomatids is characterized by intensive metabolic exchanges, reducing the nutritional requirements of these trypanosomatids when compared to species without the symbiotic bacterium, or to aposymbiotic strains. Several biochemical studies have been carried out analyzing the biosynthetic pathways involved in this intricate relationship as recently reviewed [76], and our genomic data corroborate these findings. A schematic description of the potential metabolic interactions concerning the metabolism of amino acids, vitamins, cofactors, and hemin is provided in Figure 7.

Both symbiotic bacteria have genes potentially encoding for all necessary enzymes for lysine, phenylalanine, tryptophan and tyrosine synthesis, in agreement with previous experimental data [40]. Tyrosine is required in the growth medium of *A. deanei* [81], but it is not essential for *S. oncolpelti* or *S. culicis* [41], [82], [83]. Here, in the symbiotic bacteria, we found enzymes involved in tyrosine synthesis, as well as indications that phenylalanine and tyrosine can be interconverted. In fact, protozoan growth is very slow in absence of phenylalanine and tryptophan [81], which may indicate that larger amounts of these amino acids are required for rapid cell proliferation.

Our data indicate that branched-chain amino acid (BCAA) synthesis mainly occurs in the symbionts except for the last step, with the branched-chain amino acid aminotransferase found in the host protozoan.

Among the pathways that (might) involve contributions from both partners, two have previously been characterized in detail, the urea cycle and heme synthesis. The urea cycle is complete in both symbiont-harboring trypanosomatids. Symbiotic bacteria contribute with ornithine carbamoyltransferase, which converts ornithine to citrulline, and with ornithine acetyltransferase, which transforms acetylornithine in ornithine. Conversely, aposymbiotic strains and symbiont-free *Crithidia* species need exogenous arginine or citrulline for cell proliferation [8] [68]. Our genomic data corroborate these studies.

Contrary to symbiont-free trypanosomatids, *A. deanei* and *S. culicis* do not require any source of heme for growth because the bacterium contains the required enzymes to produce heme precursors that complete the heme synthesis pathway in the host cell [7], [9], [10], [11], [84]. Our results support the idea that heme biosynthesis is mainly accomplished by the endosymbiont, with the last three steps of this pathway performed by the host trypanosomatid, and in most cases also by the bacterium as described in [11]. Furthermore, this metabolic route may represent the result of extensive gene loss and multiple lateral gene transfer events in trypanosomatids [11].

According to our genomic analyses, the symbiotic bacteria also perform the synthesis of histidine, folate, riboflavin, and coenzyme A, but one step is missing in the middle of each pathway, making them candidates for metabolic interchange with the host. In the case of folate and coenzyme A biosynthesis, one candidate gene was found in the host trypanosomatid. Moreover, none of these four metabolites are required in the growth medium of *A. deanei* and *S. culicis* [85], suggesting that these pathways are fully functional.

Candidate genes for the ubiquinone biosynthetic pathway were found in *S. culicis* but none for *A. deanei* endosymbionts. For the route with chorismate as precursor, only the first out of nine steps is missing in the *S. culicis* endosymbiont; moreover a candidate gene for that step is found in *S. culicis* genome. Only a few steps of these pathways are absent in *A. deanei* and *S. culicis* host organisms. In *L. major*, the ubiquinone ring synthesis has been described as having either acetate (via chorismate as in prokaryotes) or aromatic amino acids (as in mammalian cells) as precursors [45].

Methionine is considered essential for the growth of *A. deanei*, *S. culicis* and *S. oncopelti* [41], [81], [82]. We were not able to identify one enzyme among the four involved in the synthesis of methionine from either pyruvate or serine via cysteine in the genomes of *A. deanei* and *S. culicis*. No candidate to complement this pathway was found in the symbiotic bacteria.

#### Purine and pyrimidine metabolism for nucleotide production

**Figure 8:**
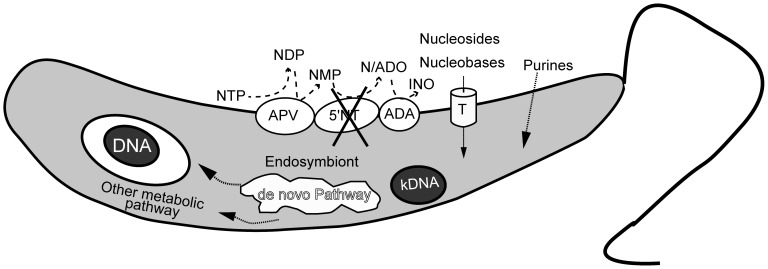
Purine production, acquisition, and utilization in *A. deanei* and *S. culicis.* The figure illustrates the production, acquisition and utilization of purines in the host trypanosomes considering the presence of endosymbiont enzymes. This model suggests that the trypanosomatid acquires purines from the symbiont, which synthesizes them *de novo*. Some ecto-localized proteins, such as apyrase (APY) and adenosine deaminase (ADA), could be responsible for the generation of extracellular nucleosides, nucleobases, and purines. Nucleobases and purines could be acquired by the parasite through membrane transporters (T) or diffusion and could be incorporated into DNA, RNA, and kDNA molecules after “purine salvage pathway” processing. Abbreviations: NTP (nucleoside tri-phosphate), NDP (nucleoside di-phosphate), NMP (nucleoside mono- phosphate), N (nucleobase), ADO (adenosine), INO (inosine).

Trypanosomatids are not able to synthesize the purine ring *de novo* [86], [87], [88]. We observed that endosymbiont-bearing trypanosomatids contain sequences encoding ectonucleotidases from the E-NTPDase family and the adenosine deaminase family (Table S18), which are required for the hydrolysis and deamination of extracellular nucleotides [89], [90]. Interestingly, sequences encoding 5′-nucleotidases are not found in either symbiont-bearing trypanosomatid. The absence of this enzyme can be related to the presence of the endosymbiont, which can supply adenosine to the host cell, as we found all genes involved in the *de novo* pathway in the symbionts, indicating that they are able to complement the purine requirements of the host (Figure 8). However, we cannot discard the possibility that adenosine is transported to the intracellular medium by carriers of monophosphate nucleoside or by the presence of other enzymes that have the same function as 5′-nucleotidase. On the other hand, the lack of 5′-nucleotidase in *A. deanei* and *S. culicis* can be related to the fact that such protozoa are only insect parasites. According to this idea, several studies have shown the importance of ectonucleotidases in the establishment of infection by some trypanosomatid species [91]. The high activity of ectonucleotidases with concomitant production of adenosine, a known immune system inhibitor, lead to high susceptibility to *Leishmania* infection because adenosine can induce anti-inflammatory effects on the host [92], [93].

Nucleoside transporters can take up nucleosides and nucleobases generated by ectonucleotidase activity. Genes encoding nucleoside transporters are present in both trypanosomatid genomes (Table S19), enabling cells to obtain exogenous purines from the medium. Furthermore, *A. deanei* and *S. culicis* contain intracellular enzymes that can convert purines to nucleotides, such as adenine phosphoribosyltransferase, hypoxanthine-guanine phosphoribosyltransferase, adenylate kinase, AMP deaminase, inosine monophosphate dehydrogenase and GMP synthetase. These data indicate that these organisms can interconvert intracellular purines into nucleotides. In contrast, both endosymbionts lack all the genes encoding enzymes related to purine salvage. Nevertheless, the symbiotic bacteria have genes encoding all the enzymes expected to participate in the *de novo* synthesis of purine nucleotides as previously proposed [94], [95]. One interesting possibility is that the symbiotic bacterium is able to supply the host trypanosomatid with purines. According to this idea, the endosymbiont participates in the *de novo* purine nucleotide pathway of *A. deanei*, as the aposymbiotic strain is unable to utilize glycine for the synthesis of purine nucleotides, only for pyrimidine nucleotide production [87].

Protozoa are generally, but not universally considered to be capable of synthesizing pyrimidines from glutamine and aspartic acid, which are used as precursors. Our results indicate that both symbiont-bearing trypanosomatids carry out *de novo* pyrimidine synthesis (Table S19). Interestingly, *in silico* analyses also revealed the presence of all the genes for *de novo* pyrimidine synthesis in both symbiont genomes, but not for the pyrimidine salvage pathway. A previous report indicated that *A. deanei* was able to synthesize purine and pyrimidine nucleotides from glycine (“de novo” pathway) and purine nucleotides from adenine and guanine (“salvage” pathway). Adenine would be incorporated into both adenine and guanine nucleotides, whereas guanine was only incorporated into guanine nucleotides, suggesting a metabolic block at the level of GMP reductase [87].

Deoxyribonucleotides are derived from the corresponding ribonucleotides by reactions in which the 2′-carbon atom of the D-ribose portion of the ribonucleotide is directly reduced to form the 2′-deoxy derivative. This reaction requires a pair of hydrogen atoms that are donated by NADPH via the intermediate-carrying protein thioredoxin. The disulfide thioredoxin is reduced by NADPH in a reaction catalyzed by thioredoxin reductase, providing the reducing equivalents for the ribonucleotide reductase, as observed for the endosymbionts that could provide 2′-deoxy derivatives. In folate metabolism, the formation of thymine nucleotides requires methylation of dUMP to produce dTMP, a reaction catalyzed by thymidilate kinase, which is present in *A. deanei*, *S. culicis*, and their respective endosymbionts. Figure 8 summarizes the purine and pyrimidine metabolisms in *A. deanei* and *S. culicis* considering the metabolic complementarity between the protozoan and the endosymbiont.

In this way, both symbiont-containing protozoa express a unique complement of nutritionally indispensable salvage and interconversion enzymes that enable the acquisition of purines from the medium. The intracellular purines can be acquired through the medium by the action of ectonucleotidases and nucleoside transporters.

### Factors Involved in Protozoa-host Interactions

Monoxenic trypanosomatids only parasitize invertebrates, especially insects belonging to the orders Diptera and Hemiptera [1]. These organisms have been found in Malphigian tubules, in the hemolymph and hemocoel, and in the midgut, which is considered the preferential site for protozoal multiplication and colonization [1], [96], [97]. *S. culicis,* for example, is able to colonize the insect midgut, to invade the hemocoel and to reach the salivary glands [97], [98]. The presence of the symbiotic bacterium has been shown to influence the interactions between trypanosomatid cells and insect cell lines, explanted guts and host insects [4], [20]. This seems to occur because the endosymbiont influences the glycoprotein and polysaccharide composition of the host, the exposure of carbohydrates on the protozoan plasma membrane, and the surface charge [18], [19], [20], [21].

Several glycosyltransferases from the two major families (GT-A and GT-B [99]) and members of the family 25 (glycosyltransferases involved in lipo-oligosaccharide protein biosynthesis) are present in both *A. deanei* and *S. culicis* genomes (Table S20). Other glycosyltransferases with no characteristic domains that are thus not classified as belonging to the GT-A or GT-B families are also found in the *A. deanei* and *S. culicis* genomes. Importantly, 1,2-fucosyltransferase transferase is present in *A. deanei* but not in the *S. culicis* dataset, and fucose residues were found in high amounts on glycoinositolphospholipid (GIPL) molecules of *A. deanei*, different from the observations for other trypanosomatids (data not published). Although the role of fucose is unknown, fucose and arabinose transfer to lipophosphoglycan (LPG) of *Leishmania* is noticed when the culture medium is supplemented with this carbohydrate [100], suggesting that fucose might have a specific role in *A. deanei*-insect interactions.

Another glycosyltransferase found in both *A. deanei* and *S. culicis* genomes and involved in the N-glycosylation of asparagine residues is the dolichyl-diphosphooligosaccharide-protein glycosyltransferase (DDOST), an oligosaccharyltransferase (OST) that is not classified in any of the above-mentioned families. The *A. deanei* and *S. culicis* DDOSTs contain the STT3 domain, a subunit required to establish the activity of the oligosaccharyl transferase (OTase) complex of proteins, and they are orthologous to the human DDOST. These OTase complexes are responsible for transferring lipid-linked oligosaccharides to the asparagine side chain of the acceptor polypeptides in the endoplasmic reticulum [101], suggesting a conserved N-glycosylation among the trypanosomatids.

Five different GalfT sequences are also present in the endosymbiont-bearing trypanosomatids, and all of them contain the proposed catalytic site, indicating genetic redundancy. Redundancy of GalfTs is commonly observed in many different trypanosomatid species, as different transferases are used for each linkage type [102]. As *β*-galactofuranose (*β*-Gal*f*) has been shown to participate in trypanosome-host interactions [103], their presence in *A. deanei* and *S. culicis* might also indicate a role in the interaction with the insect host. However, no enzymes involved in synthesis of *β*-Galf-containing glycoconjugates are detected in our *A. deanei* dataset, despite reports of enzymes involved in *β*-Gal*f* synthesis in *Crithidia* spp. [104], [105], [106].

#### Surface proteins and protease gene families

One remarkable characteristic of trypanosomatid genomes is the large expansion of gene families encoding surface proteins [107]. Experimental data indicated that these genes encode surface proteins involved in interactions with the hosts. We selected eight gene families encoding surface proteins present in *T. cruzi, T. brucei* and *Leishmania* spp. to search for homologous sequences in the genomes of the two symbiont-bearing trypanosomatids. Because the draft assemblies of these genomes are still fragmented, we also used a read-based analysis to search for sequences with homology to these multigene families. It is well known that misassemblies frequently occur for tandemly repeated genes, as most repetitive copies collapse into only one or two copies. A total of 3,624,411 reads (corresponding to 1,595 Mb of sequences) from the *A. deanei* genome and 2,666,239 reads (corresponding to 924 Mb) from the *S. culicis* genome were used in this comparison. In *A. deanei* and *S. culicis*, we identified gene families encoding amastins, gp63, and cysteine peptidases (Table S21). As expected, we could not identify sequences homologous to mucin-like glycoproteins typical of *T. cruzi* [108], variant surface glycoprotein (VSG) characteristic of African trypanosomes, or trans-sialidases present in the genomes of all *Trypanosoma* species.

Calpain-like cysteine peptidases constitute the largest gene family identified in the *A. deanei* (85 members) and *S. culicis* (62 members) genomes, and they are also abundant in trypanosomatids [46]. The presence of the N-terminal fatty acid acylation motif was found in some members of calpain-like cysteine peptidases, indicating that some of these peptidases are associated with membranes, as has also been shown for other members of the family [109], [110]. The relatively large amount of calpain-like peptidases may be related to the presence of the endosymbiont, which would require a more complex regulation of the cell cycle and intracellular organelle distribution [14], as cytosolic calpains were found to regulate cytoskeletal remodeling, signal transduction, and cell differentiation [46].

A second large gene family in the *A. deanei* and *S. culicis* genomes encoding surface proteins with proteolytic activity is gp63. In our genomic analyses, we identified 37 and 9 genes containing sequences homologous to the gp63 of *Leishmania* and *Trypanosoma* spp. in the genomes of *A. deanei* and *S. culicis*, respectively. Proteins belonging to this group of zinc metalloproteases, also known as major surface protease (MSP) or leishmanolysin, have been characterized in various species of *Leishmania* and *Trypanosoma* [111]. Extensive studies on the role of this family in *Leishmania* indicate that they are involved in several aspects of host-parasite interaction including resistance to complement-mediated lysis, cell attachment, entry, and survival in macrophages [112]. Gene deletion studies in *T. brucei* indicated that the TbMSP of bloodstream trypanosomes acts in concert with phospholipase C to remove the variant surface protein from the membrane, required for parasite differentiation into the procyclic insect form [113]. Gp63-like molecules have been observed on the cell surface of symbiont-harboring trypanosomatids [114]. Importantly, the symbiont containing *A. deanei* displays a higher amount (2-fold) of leishmanolysin-like molecules at the surface compared to the aposymbiotic strain, which are unable to colonize insects [4]. As anti-gp63 antibodies decrease protozoan-insect interactions [21], our results reinforce the idea that the presence of such interactions caused the expansion of this gene family in endosymbiont-bearing organisms.

In contrast, only two copies of lysosomal cathepsin-like cysteine peptidases were identified in the *A. deanei* (AGDE05983 and AGDE10254) and *S. culicis* genomes (STCU01417 and STCU06430). The two *A. deanei* sequences encode identical cathepsin-B-like proteins, whereas the two *S. culicis* genes encode proteases of the cathepsin-L-like group. This class of cysteine peptidase is represented by cruzain or cruzipain, major lysosomal proteinases of *T. cruzi* expressed by parasites found in insect and vertebrate hosts, and encoded by a large gene family [115], [116]. In *T. cruzi,* these enzymes have important roles in various aspects of the host/parasite relationship and in intracellular digestion as a nutrient source [115]. Conversely, the low copy number of this class of lysosomal peptidase in symbiont-containing trypanosomatids seems to be related to their low nutritional requirements.

Amastins constitute a third large gene family in the *A. deanei* and *S. culicis* genomes that encodes surface proteins. Initially described in *T. cruzi* [117], amastin genes have also been identified in various *Leishmania* species [118], in *A. deanei* and in another related insect parasite, *Leptomonas seymouri* [119]. In *Leishmania*, amastins constitute the largest gene family with gene expression that is regulated during the parasite life cycle. As amastin has no sequence similarity to any other known protein, its function remains unknown. In this work, we identified 31 genes with sequences belonging to all four sub-families of amastins in the genome of *A. deanei* and 14 copies of amastin genes in *S. culicis*. Similar to *Leishmania*, members of all four amastin subfamilies were identified in symbiont-containing species (see Figure S7).

### Conclusion

The putative proteome of symbiont-bearing trypanosomatids revealed that these microorganisms exhibit unique features when compared to other protozoa of the same family and that they are most closely related to *Leishmania* species. Most relevant are the differences in the genes related to cytoskeleton, paraflagellar and kinetoplast structures, along with a unique pattern of peptidase gene organization that may be related to the presence of the symbiont and of the monoxenic life style. The symbiotic bacteria of *A. deanei* and *S. culicis* are phylogenetically related with a common ancestor, most likely a β-proteobacteria of the Alcaligenaceae family. The genomic content of these symbionts is highly reduced, indicating gene loss and/or transfer to the host cell nucleus. In addition, we confirmed that both bacteria contain genes that encode enzymes that complement several metabolic routes of the host trypanosomatids, supporting the fitness of the symbiotic relationship.

## Supporting Information

Figure S1 **Evolutionary history of endosymbionts obtained through a phylogenomic approach**. The figure indicates analysis using the Neighbor joining (NJ) **(A)** and Maximum parsimony (MP) **(B)** methods. For NJ and MP, the percentage of replicate trees in which the associated taxa clustered together in the bootstrap test (1,500 replicates) is shown next to the branches. The scale bar represents amino acids substitutions per site.
(TIF)

Figure S2 **Amino acid alignment of Kinetoplast Associated Proteins**. Panel (A) shows the KAP4 ClustalW alignment of *A. deanei* (AdKAP-4), *S. culicis* (ScKAP-4) and *C. fasciculata* (CfKAP-4). Panel (B) shows the ClustalW alignment of KAP2 of *S. culicis* and *C. fasciculata* (CfKAP2-2, GenBank Q9TY84 and CfKAP2-1 GeneBank Q9TY83). Black color highlight is 100% similar gray is 80 to 99% similar light gray is 60 to 79% similar white is less than 59% similar.
(TIF)

Figure S3 **Comparison of the histone sequences of** ***A. deanei*** **and** ***S. culicis*** **with other trypanosomes**. Residues indicated in red correspond to lysines that are acetylated and green, methylated in *T. cruzi* and *T. brucei* [121]. Residues indicated in blue are predicted site for phosphorylation upon DNA damage as shown in *T. brucei* [122].
(TIF)

Figure S4 **Phylogenetic tree of sirtuins from Trypanosomatids**. The numbers represent bootstrap values. The proteins from each species are grouped in nuclear and mitochondrial Sir2 based on the sequences of *S. cerevisiae* (nuclear), and the similarity with *S. coelicolor* and *S. enterica.*
(TIF)

Figure S5 **Phylogenetic tree of spliced leader (SL) sequences of** ***A. deanei*** **and** ***S. culicis*** **.** A neighbor-joining tree (1000 bootstraps) obtained by MEGA 5.0 using the SL gene from the *A. deanei* and *S. culicis* genome sequences and sequences retrieved from GenBank (*S. culicis* DQ860203.1, *L. pyrrhocoris* JF950600.1, *H. samuelpessoai* X62331.1, *H. mariadeanei* AY547468.1, *A. deanei* EU099545.1, *T. rangeli* AF083351 and *T. cruzi* AY367127).
(TIF)

Figure S6 **Comparison between the amino acid sequences of** ***S. culicis*** **CRK sequences.** The figure shows a ClustalW alignment with the ATP binding domains boxed in yellow, PSTAIRE motifs boxed in blue, and the catalytic domain boxed in pink. Red residues indicate the observed variations in the amino acids involved in the activity.
(TIF)

Figure S7 **Tree showing the distribution of amastin sub-families in** ***A. deanei***. The amastins are grouped as delta-amastin (red), gamma-amastins (yellow), alpha-amastins (dark blue) and beta-amastins (light blue).
(TIF)

Table S1 **ORFs identified as Kinetoplast-associated protein (KAPs) in** ***A. deanei*** **and** ***S. culicis.***
(DOC)

Table S2 **Histone acetyltransferases of the MYST family present in** ***A. deanei*** **and** ***S. culicis*** **compared to other trypanosomes.**
(DOC)

Table S3 **Distribution of Sirtuins in the protozoan and endosymbiont species.**
(DOC)

Table S4 **Histone deacetylase identified in** ***A. deanei*** **and** ***S. culicis.***
(DOC)

Table S5 **Histone methyltransferase in** ***A. deanei*** **and** ***S. culicis.***
(DOC)

Table S6 Histone chaperones identified in *A. deanei* and *S. culicis.*
(DOC)

Table S7 **Bromodomain proteins found in** ***A. deanei*** **and** ***S. culicis.***
(DOC)

Table S8 Components of replication mechanism of the kDNA identified in *A. deanei* and *S. culicis* and similar endosymbionts ORFs.
(DOC)

Table S9 Identified ORFs related to DNA replication and DNA repair in *A. deanei* and *S. culicis.*
(DOC)

Table S10 DNA replication and repair ORFs found in the *A. deanei* and *S. culicis* endosymbionts.
(DOC)

Table S11 Identified ORFs involved in DNA transcription and RNA splicing in the genome of *A. deanei* and *S. culicis*.
(DOC)

Table S12 Transcription related proteins in the endosymbionts of *A. deanei* and *S. culicis.*
(DOC)

Table S13 Main ORFs detected participating in ribosomal biogenesis and translation *in A. deanei* and *S. culicis.*
(DOC)

Table S14 Identified phosphatases in *A. deanei* and *S. culicis.*
(DOC)

Table S15 Number of heat shock and stress response proteins in *A. deanei* and *S.culicis.*
(DOC)

Table S16 Glycerophospholipids (GPL) enzymes of *A. deanei* and *S. culicis^1^.*
(DOC)

Table S17 **Glycerophospholipids (GPL) enzymes of** ***A. deanei and S. culicis*** **endosymbionts.**
(DOC)

Table S18 **Ectonucleotidases families and identification of ORFs found in** ***A. dean*** **ei and** ***S. culicis*** **.**
(DOC)

Table S19 **ORFs encoding enzymes involved in purine and pyrimidine metabolism of** ***A. deanei*** **,** ***S. culicis*** **and their symbionts.**
(DOC)

Table S20 **Glysosyltransferases found in** ***A. deanei*** **and** ***S. culicis.***
(DOC)

Table S21 Surface proteins of *A. deanei* e *S. culicis.*
(DOC)

Text S1 (DOC)
