# Supplementary figures and images for: Predicting the Proteins of Angomonas deanei, Strigomonas culicis and Their Respective Endosymbionts Reveals New Aspects of the Trypanosomatidae Family
Source: PLoS One. 2013 Apr 3;8(4):e60209. doi: 10.1371/journal.pone.0060209 (PMC3616161; doi:10.1371/journal.pone.0060209)

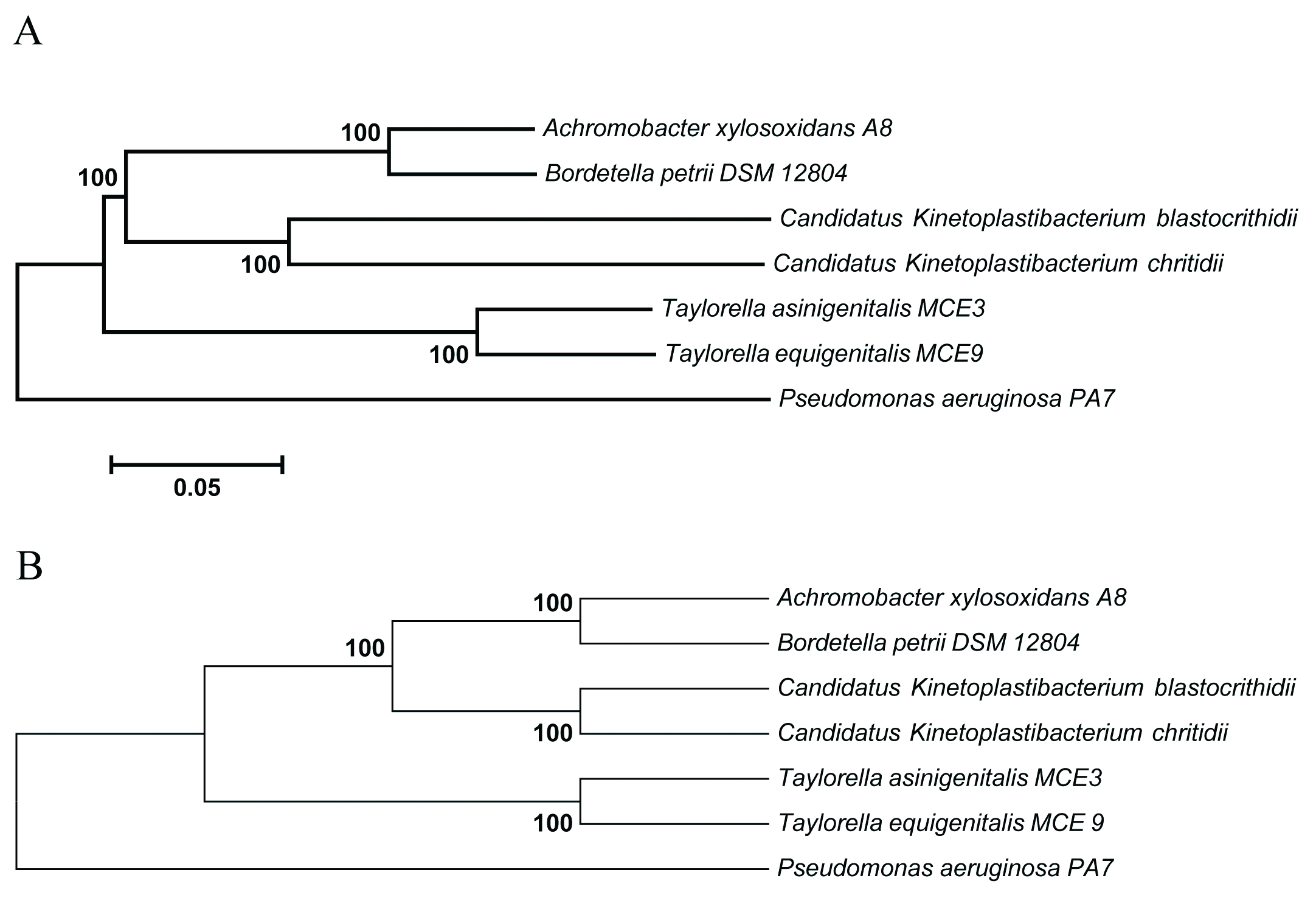

Supplement: Figure S1 — Evolutionary history of endosymbionts obtained through a phylogenomic approach. The figure indicates analysis using the Neighbor joining (NJ) (A) and Maximum parsimony (MP) (B) methods. For NJ and MP, the percentage of replicate trees in which the associated taxa clustered together in the bootstrap test (1,500 replicates) is shown next to the branches. The scale bar represents amino acids substitutions per site. (TIF) [file pone.0060209.s001.tif]

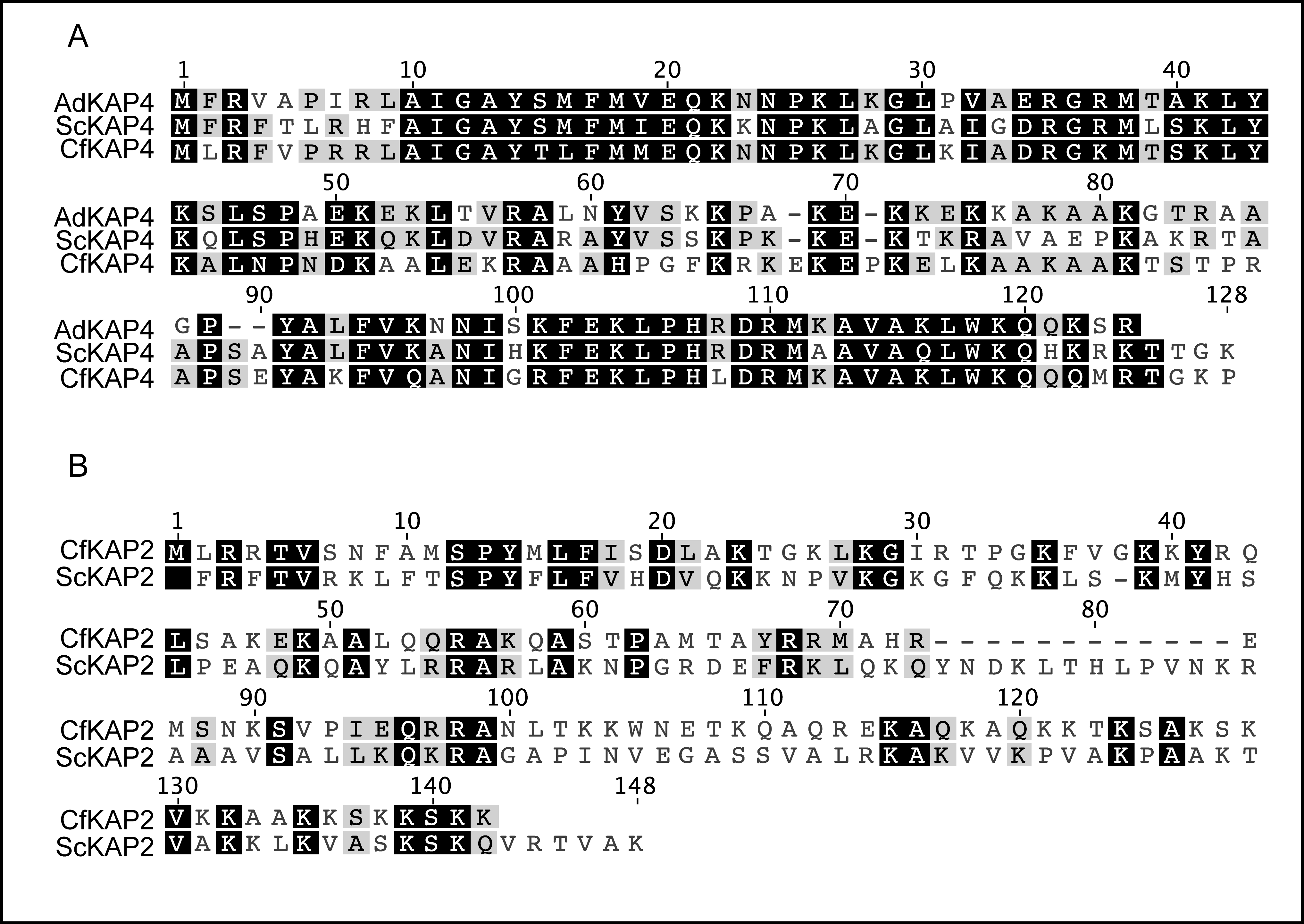

Supplement: Figure S2 — Amino acid alignment of Kinetoplast Associated Proteins. Panel (A) shows the KAP4 ClustalW alignment of A. deanei (AdKAP-4), S. culicis (ScKAP-4) and C. fasciculata (CfKAP-4). Panel (B) shows the ClustalW alignment of KAP2 of S. culicis and C. fasciculata (CfKAP2-2, GenBank Q9TY84 and CfKAP2-1 GeneBank Q9TY83). Black color highlight is 100% similar gray is 80 to 99% similar light gray is 60 to 79% similar white is less than 59% similar. (TIF) [file pone.0060209.s002.tif]

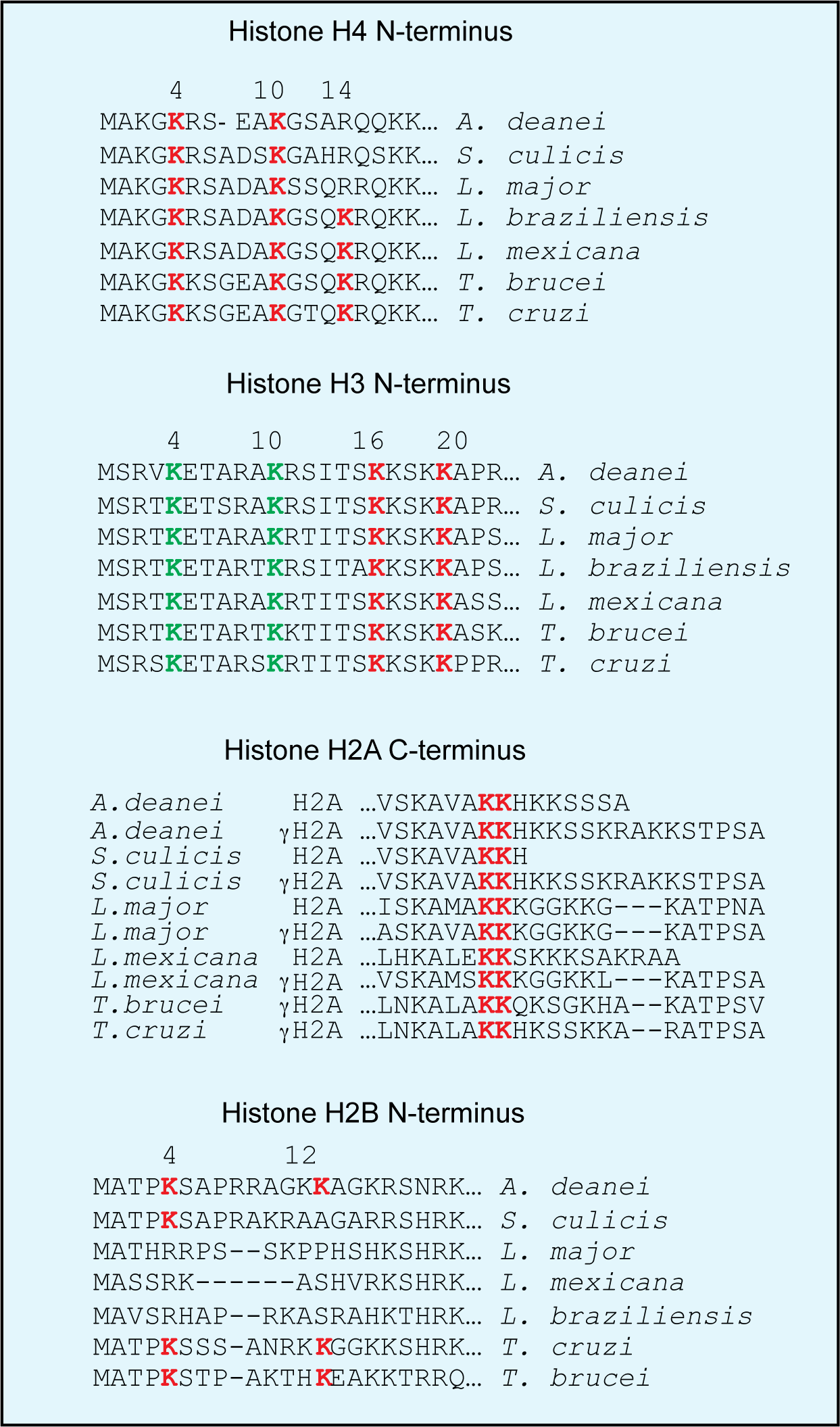

Supplement: Figure S3 — Comparison of the histone sequences of A. deanei and S. culicis with other trypanosomes. Residues indicated in red correspond to lysines that are acetylated and green, methylated in T. cruzi and T. brucei [121]. Residues indicated in blue are predicted site for phosphorylation upon DNA damage as shown in T. brucei [122]. (TIF) [file pone.0060209.s003.tif]

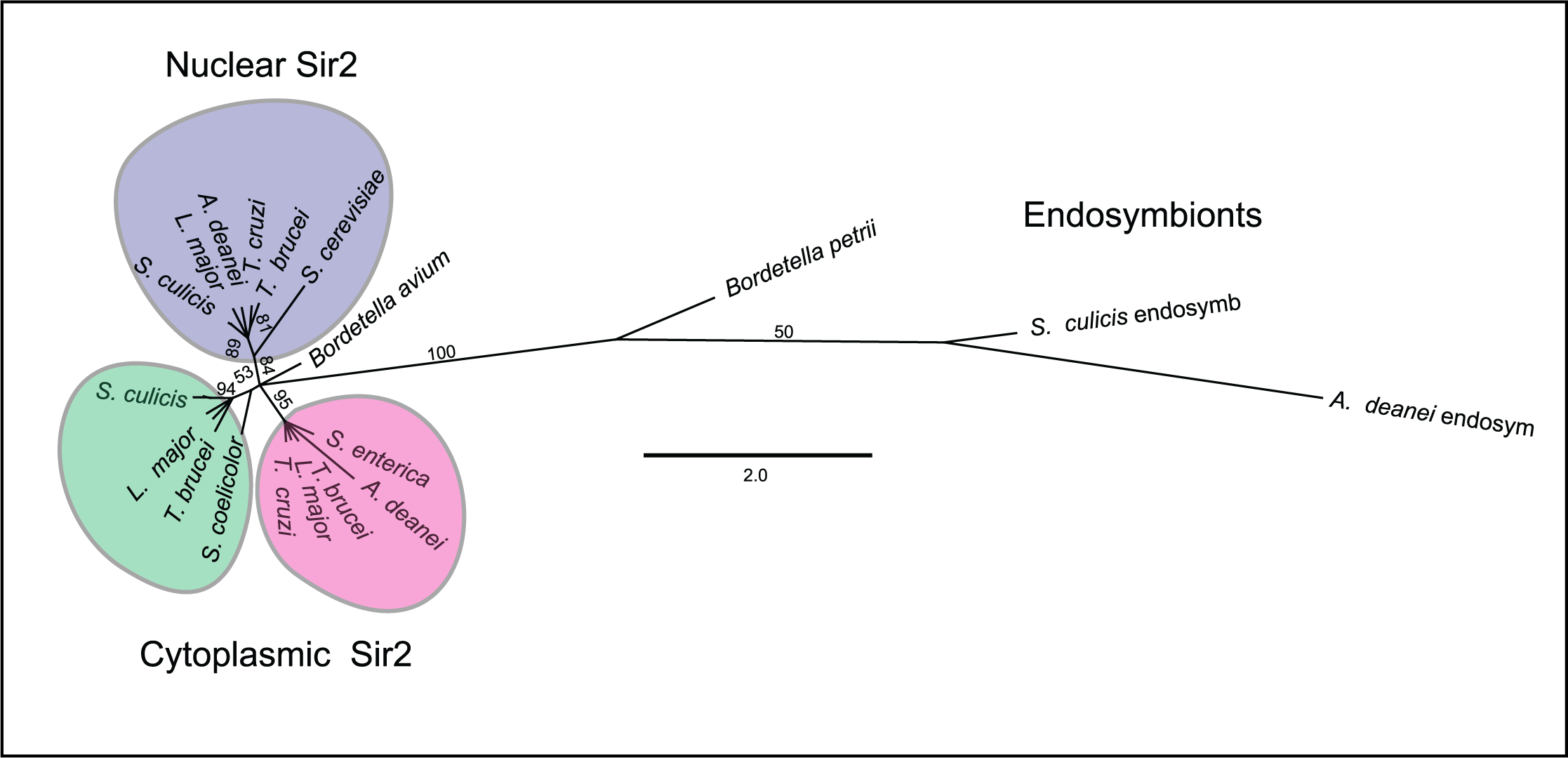

Supplement: Figure S4 — Phylogenetic tree of sirtuins from Trypanosomatids. The numbers represent bootstrap values. The proteins from each species are grouped in nuclear and mitochondrial Sir2 based on the sequences of S. cerevisiae (nuclear), and the similarity with S. coelicolor and S. enterica. (TIF) [file pone.0060209.s004.tif]

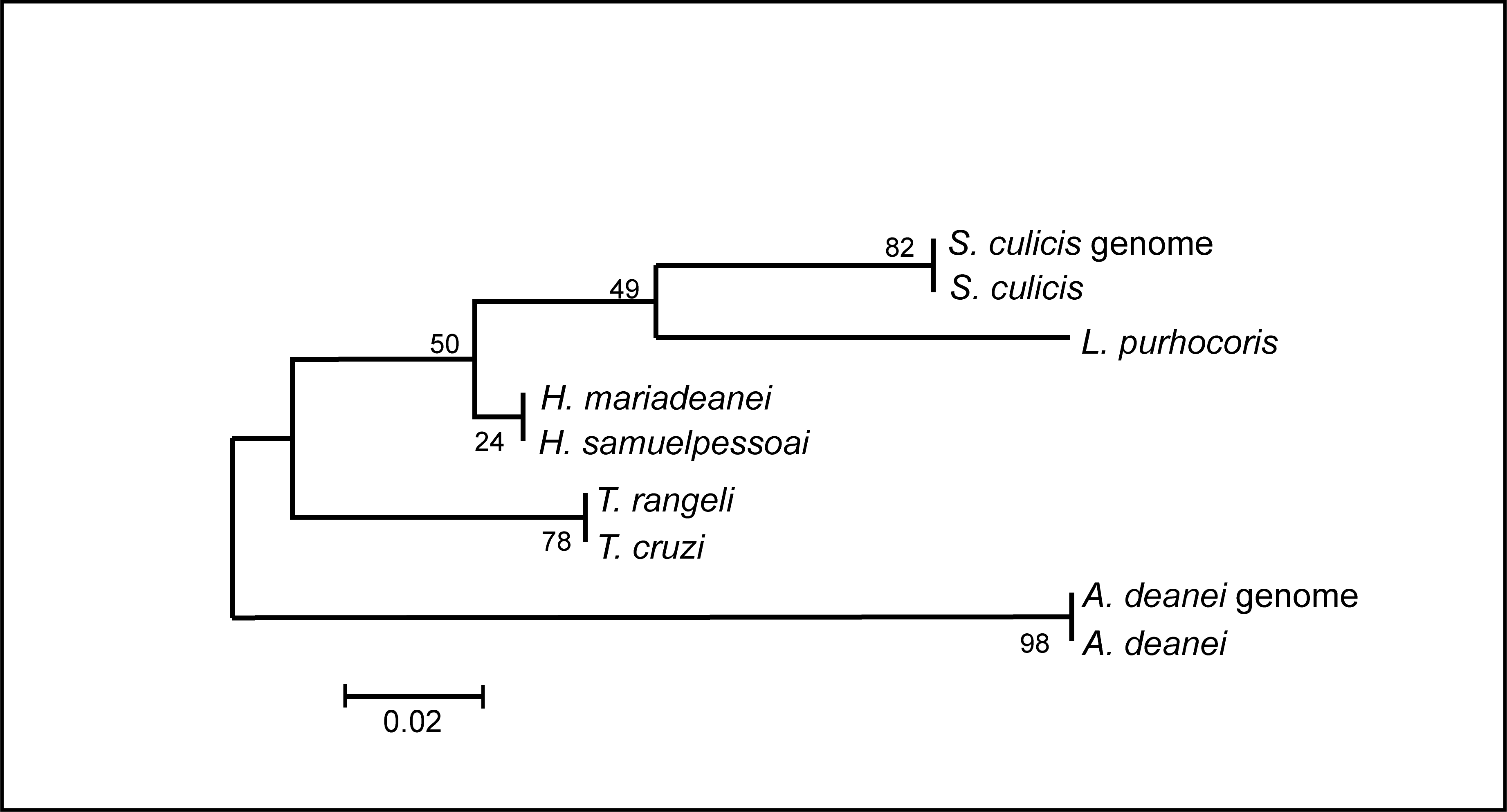

Supplement: Figure S5 — Phylogenetic tree of spliced leader (SL) sequences of A. deanei and S. culicis . A neighbor-joining tree (1000 bootstraps) obtained by MEGA 5.0 using the SL gene from the A. deanei and S. culicis genome sequences and sequences retrieved from GenBank (S. culicis DQ860203.1, L. pyrrhocoris JF950600.1, H. samuelpessoai X62331.1, H. mariadeanei AY547468.1, A. deanei EU099545.1, T. rangeli AF083351 and T. cruzi AY367127). (TIF) [file pone.0060209.s005.tif]

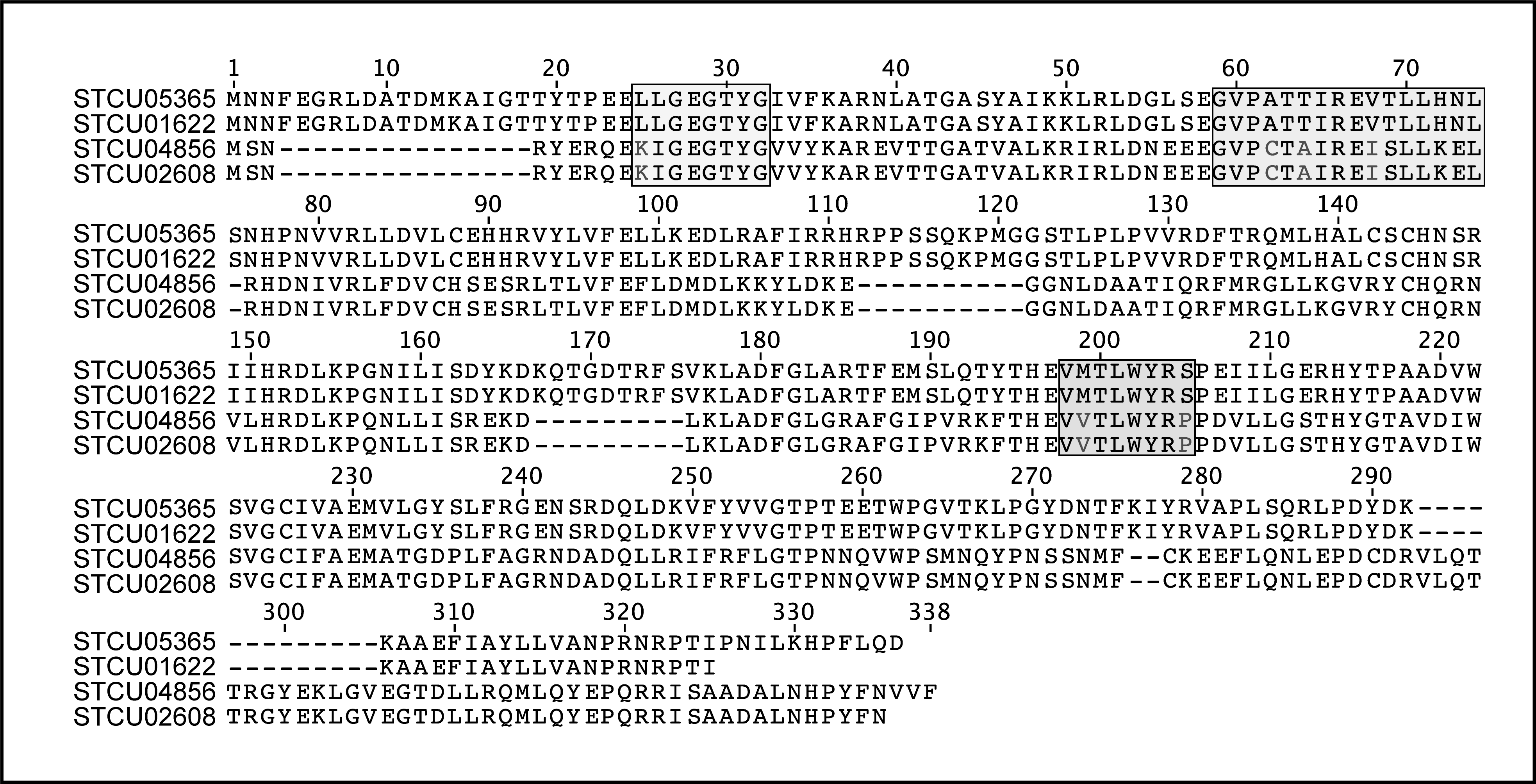

Supplement: Figure S6 — Comparison between the amino acid sequences of S. culicis CRK sequences. The figure shows a ClustalW alignment with the ATP binding domains boxed in yellow, PSTAIRE motifs boxed in blue, and the catalytic domain boxed in pink. Red residues indicate the observed variations in the amino acids involved in the activity. (TIF) [file pone.0060209.s006.tif]

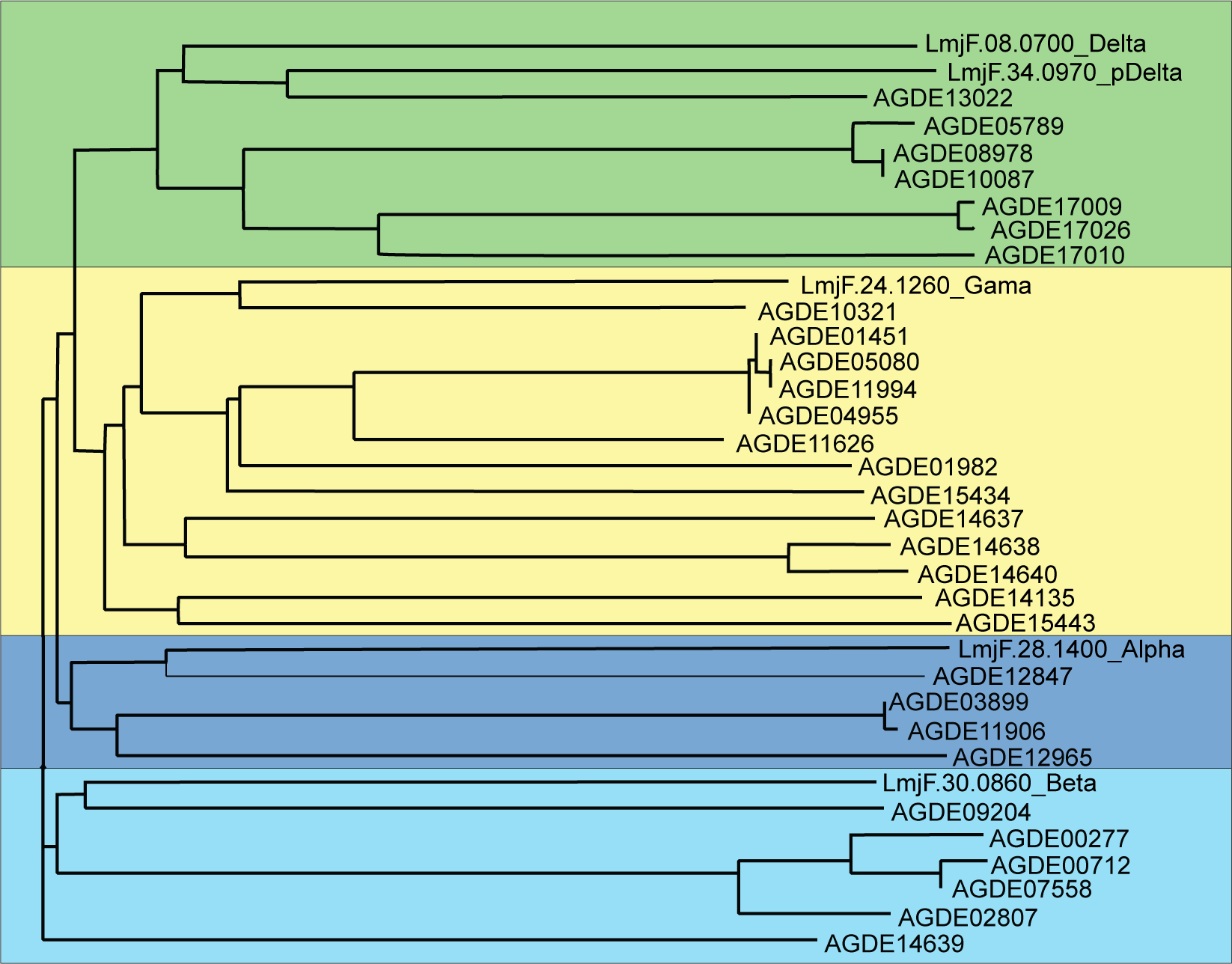

Supplement: Figure S7 — Tree showing the distribution of amastin sub-families in A. deanei. The amastins are grouped as delta-amastin (red), gamma-amastins (yellow), alpha-amastins (dark blue) and beta-amastins (light blue). (TIF) [file pone.0060209.s007.tif]
