# Supplementary material for: Predicting the Proteins of Angomonas deanei, Strigomonas culicis and Their Respective Endosymbionts Reveals New Aspects of the Trypanosomatidae Family
Source: PLoS One. 2013 Apr 3;8(4):e60209. doi: 10.1371/journal.pone.0060209 (PMC3616161; doi:10.1371/journal.pone.0060209)
Supplement: Table S1 — ORFs identified as Kinetoplast-associated protein (KAPs) in A. deanei and S. culicis. (DOC) [file pone.0060209.s008.doc]

***Table S1****. ORFs identified as Kinetoplast-associated protein (KAPs) in A. deanei and S. culicis.*

| **KAPs** | **ORFs ID (nt/aa)** | **kDa** | **Isoeletric point** | **Alanine/Lysine %** | **Score** | **Query coverage** | **Subject coverage** | **Organism** |
| --- | --- | --- | --- | --- | --- | --- | --- | --- |
| *A. deanei* |  |  |  |  |  |  |  |  |
| AdKAP4 | AGDE01956 (363/120) | 13 | 11.41 | 13.3/19.2 | 142 | 100 | 97.58 | *L. major* |
| AdKAP3 | AGDE13071 (444/ 147) | 16 | 12 | 12.3/25.3 | 57.4 | 32 | 52 | *L. braziliensis* |
| *S.culicis* |  |  |  |  |  |  |  |  |
| ScKAP4 | STCU01892 (381/126) | 14 | 11.57 | 13.5/16.7 | 151 | 99.2 | 99.19 | *L. major* |
| ScKAP2 | STCU02484 (444/147) | 16 | 11.88 | 12.9/18.4 | 63 | 92.47 | 93.71 | *L. braziliensis* |
| ScKAP-like | STCU04568 (558/185) | 21 | 11.81 | 9.7/23.2 | 77 | 69.02 | 98.37 | *T. cruzi* |
