# Supplementary material for: Predicting the Proteins of Angomonas deanei, Strigomonas culicis and Their Respective Endosymbionts Reveals New Aspects of the Trypanosomatidae Family
Source: PLoS One. 2013 Apr 3;8(4):e60209. doi: 10.1371/journal.pone.0060209 (PMC3616161; doi:10.1371/journal.pone.0060209)
Supplement: Table S2 — Histone acetyltransferases of the MYST family present in A. deanei and S. culicis compared to other trypanosomes. (DOC) [file pone.0060209.s009.doc]

**Table S2**. Histone acetyltransferases of the MYST family present in *A. deanei* and *S. culicis* compared to other trypanosomes.

| **Organism** | **HAT1** | **HAT2 (H4K10)^a^** | **HAT3 (H4K4)^b^** | **HAT4** |
| --- | --- | --- | --- | --- |
| *A. deanei* | AGDE03440 AGDE07131 AGDE11732 AGDE08858 | AGDE07993 | AGDE06446 | nd |
| *S. culicis* | STCU03841 STCU04835 STCU02005 | nd | nd | nd |
| *L. major* | XM_01687561 | XM_001684459 | XP_001687295 | XM_001681709 |
| *T. cruzi* | XM_098466 | XP_809124 | XM_816074 | XP_802230 |
| *T. brucei* | XP_834196 | XP_829218 | XM_817972 | nd |

^a^ Indicates that HAT2 acetylates preferentially H4K10

^b^ Indicates that HAT3 acetylates preferentially H4K4

nd: not determined
