# Supplementary material for: Predicting the Proteins of Angomonas deanei, Strigomonas culicis and Their Respective Endosymbionts Reveals New Aspects of the Trypanosomatidae Family
Source: PLoS One. 2013 Apr 3;8(4):e60209. doi: 10.1371/journal.pone.0060209 (PMC3616161; doi:10.1371/journal.pone.0060209)
Supplement: Table S3 — Distribution of Sirtuins in the protozoan and endosymbiont species. (DOC) [file pone.0060209.s010.doc]

**Table S3**. Distribution of Sirtuins in the protozoan and endosymbiont species.

|  | ***A. deanei*** | ***S. culicis*** | ***A. deanei* endosymbiont** | ***S. culicis* endosymbiont** |
| --- | --- | --- | --- | --- |
| Sir2p-1 | AGDE09340 | STCU07010 STCU08823 | nd | nd |
| Sir2p-2 | AGDE01242 | STCU09303 STCU03962 STCU05369 | CKCE00177 | CKBE00538 |
| Sir2p-3 | AGDE01423 | nd | nd | nd |

nd: not determined
