# Supplementary material for: Predicting the Proteins of Angomonas deanei, Strigomonas culicis and Their Respective Endosymbionts Reveals New Aspects of the Trypanosomatidae Family
Source: PLoS One. 2013 Apr 3;8(4):e60209. doi: 10.1371/journal.pone.0060209 (PMC3616161; doi:10.1371/journal.pone.0060209)
Supplement: Table S4 — Histone deacetylase identified in A. deanei and S. culicis. (DOC) [file pone.0060209.s011.doc]

**Table S4**. Histone deacetylase identified in *A. deanei* and *S. culicis.*

| **Organism** | **HDAC1** | **HDAC2** | **HDAC4** | **HDAC3** |
| --- | --- | --- | --- | --- |
|  |
| *A. deanei* | AGDE04277 | AGDE11219 | AGDE10819 AGDE11711 | AGDE15138* |
| *S. culicis* | STCU02890 STCU03191 STCU04836 | STCU05799 STCU6139 | STCU05676 | STCU04836* |
| *L. major* | XP_001683001 | nd | XP_001681138 | XP_001683153 |
| *T. brucei* | XP_822424 | XP_829617 | XP_844962 | XP_951529 |
| *T. cruzi* | XP_816761 | XP810218 | EFZ31623 | XP_807406 |

* partial sequences

nd: not determined
