# Supplementary material for: Predicting the Proteins of Angomonas deanei, Strigomonas culicis and Their Respective Endosymbionts Reveals New Aspects of the Trypanosomatidae Family
Source: PLoS One. 2013 Apr 3;8(4):e60209. doi: 10.1371/journal.pone.0060209 (PMC3616161; doi:10.1371/journal.pone.0060209)
Supplement: Table S5 — Histone methyltransferase in A. deanei and S. culicis. (DOC) [file pone.0060209.s012.doc]

**Table S5.** Histone methyltransferase in *A. deanei* and *S. culicis.*

| **Dot1a** |  | |  |
| --- | --- | --- | --- |
| AGDE01030 | | Histone-lysine N-methyltransferase | |
| AGDE04810 | | Histone-lysine N-methyltransferase | |
| AGDE09256 | | Histone-lysine N-methyltransferase | |
| AGDE11398 | | Histone-lysine N-methyltransferase | |
| AGDE07089 | | Histone-lysine N-methyltransferase | |
| STCU07419 | | Histone-lysine N-methyltransferase | |
| STCU03859 | | Histone-lysine N-methyltransferase | |
| STCU04149 | | Histone-lysine N-methyltransferase | |
| STCU05004 | | Histone-lysine N-methyltransferase | |
| **Dot1b** |  | |  |
| STCU03830 | | Histone-lysine N-methyltransferase | |
| STCU04696 | | Histone-lysine N-methyltransferase | |
| AGDE07380 | | Histone-lysine N-methyltransferase | |
