# Supplementary material for: Predicting the Proteins of Angomonas deanei, Strigomonas culicis and Their Respective Endosymbionts Reveals New Aspects of the Trypanosomatidae Family
Source: PLoS One. 2013 Apr 3;8(4):e60209. doi: 10.1371/journal.pone.0060209 (PMC3616161; doi:10.1371/journal.pone.0060209)
Supplement: Table S6 — Histone chaperones identified in A. deanei and S. culicis. (DOC) [file pone.0060209.s013.doc]

***Table S6****. Histone chaperones identified in A. deanei and S. culicis.*

| **Histone chaperones** | ***T. brucei*** | ***T. cruzi*** | ***A. deanei*** | ***S. culicis*** |
| --- | --- | --- | --- | --- |
| Asf1A | XP_847419 | XP_804312 | AGDE02025 AGDE02390 AGDE03410 | STCU03930 |
| Asf1B | XP_001218766 | XP_811097 | AGDE01577 AGDE12216 | STCU00677 STCU07248 |
| CAF-1A | XP_847229 | XP_813006 | nd | STCU07187 |
| CAF-1B | XP_822940 | XP_812435 | AGDE11894 AGDE07023 AGDE04339 | STCU06597 |
| CAF-1 C (RPB4) | XP_828751 | XP_816613 | AGDE06495 AGDE15488 | nd |
| FACT complex superfamily Rtt106 | XP_803896 | XP_803896 | AGDE10534 | nd |
| Superfamily Rtt106 | XP_827919 | XP_820949 | nd | nd |
| Superfamily Rtt106 | XP_803501 | XP_820787 | nd | nd |
| Nap1a | XP_826968 | XP_806215 | AGDE00105 AGDE03189 | STCU00093 |
| Nap1b | XP_809789 | XP_827991 | AGDE03794 AGDE10804 AGDE11137 | STCU00560 |
| Chz1 | nd | nd | nd | nd |
| Vps75 | nd | nd | nd | nd |

nd: not determined
