# Supplementary material for: Predicting the Proteins of Angomonas deanei, Strigomonas culicis and Their Respective Endosymbionts Reveals New Aspects of the Trypanosomatidae Family
Source: PLoS One. 2013 Apr 3;8(4):e60209. doi: 10.1371/journal.pone.0060209 (PMC3616161; doi:10.1371/journal.pone.0060209)
Supplement: Table S7 — Bromodomain proteins found in A. deanei and S. culicis. (DOC) [file pone.0060209.s014.doc]

**Table S7**. Bromodomain proteins found in *A. deanei* and *S. culicis.*

| **Proteins** | ***T. brucei*** | ***T. cruzi*** | ***A. deanei*** | ***S. culicis*** |
| --- | --- | --- | --- | --- |
| Bromodomain 1 | XP_823049 | XP_820322 | AGDE04649 AGDE08056 | nd |
| Bromodomain 2 | XP_822977 | XP_804291 | AGDE01925 AGDE04282 | STCU01519 |
| Bromodomain 3 | XP_829071 | XP_819186 | AGDE06532 AGDE00452 AGDE11752 | nd |
| Bromodomain 4 | XP_846061 | XP_815279 | AGDE15741 | nd |

nd: not determined
