# Supplementary material for: Predicting the Proteins of Angomonas deanei, Strigomonas culicis and Their Respective Endosymbionts Reveals New Aspects of the Trypanosomatidae Family
Source: PLoS One. 2013 Apr 3;8(4):e60209. doi: 10.1371/journal.pone.0060209 (PMC3616161; doi:10.1371/journal.pone.0060209)
Supplement: Table S8 — Components of replication mechanism of the kDNA identified in A. deanei and S. culicis and similar endosymbionts ORFs. (DOC) [file pone.0060209.s015.doc]

**Table S8.** Components of replication mechanism of the kDNA identified in *A. deanei* and *S. culicis* and similar endosymbionts ORFs.

| **Replication protein** | ***A. deanei*** | ***A. deanei* endosymbiont** | ***S. culicis*** | ***S. culicis* endosymbiont** |
| --- | --- | --- | --- | --- |
| POLβ | AGDE09058 | CKCE00574 | STCU00334 | CKBE00168 |
| POL IC (mitochondrial DNA polymerase I protein C) | AGDE11399 | nd | STCU08997 | nd |
| POL IB (mitochondrial DNA polymerase I protein B) | AGDE07691 | nd | STCU07812 | nd |
| POL III gamma (DNA polymerase III gamma) | nd | CKCE00702 | nd | nd |
| POL III alpha (DNA polymerase III alpha) | nd | CKCE00022 | nd | CKBE00125 |
| POL III epsilon (DNA Polymerase III epsilon) | AGDE12104 | CKCE00417 | STCU06882 | CKBE00012 |
| SSE1 (mitochondrial structure specific endonuclease I) | AGDE00590 | nd | STCU00666 | nd |
| Polβ-PAK | AGDE06217 | nd | STCU09890 | nd |
| PRIMASE | AGDE02301 | CKCE00220 | STCU05882 | CKBE00499 |
| LIGASE K α | AGDE03121 | nd | STCU06213 | nd |
| LIGASE | AGDE02430 | CKCE00118 | STCU01088 | CKBE00597 |
| HELICASE | AGDE06708 | CKCE00231 | STCU07762 | CKBE00488 |
| HELICASE II | nd | CKCE00367 | nd | CKBE00361 |
| UMSBP | AGDE15517 | nd | STCU09737 | nd |
| TOPOI (DNA topoisomerase IA) | AGDE12723 | nd | STCU04943 | nd |
| TOPO II (DNA topoisomerase II) | AGDE08128 | nd | STCU05816 | nd |
| TOPO III (DNA topoisomerase III) | AGDE11015 | CKCEOO577 | STCU06411 | CKBE00171 |

nd: not determined
