# Supplementary material for: Predicting the Proteins of Angomonas deanei, Strigomonas culicis and Their Respective Endosymbionts Reveals New Aspects of the Trypanosomatidae Family
Source: PLoS One. 2013 Apr 3;8(4):e60209. doi: 10.1371/journal.pone.0060209 (PMC3616161; doi:10.1371/journal.pone.0060209)
Supplement: Table S9 — Identified ORFs related to DNA replication and DNA repair in A. deanei and S. culicis. (DOC) [file pone.0060209.s016.doc]

**Table S9.** Identified ORFs related to DNA replication and DNA repair in *A. deanei* and *S. culicis.*

| **DNA Replication** | ***S. culicis*** | ***A. deanei*** |
| --- | --- | --- |
| DNA ligase 1 | STCU05790 | AGDE06621 |
| DNA ligase 1 | nd | AGDE07592 |
| DNA ligase 1 | nd | AGDE08329 |
| DNA ligase I | STCU00307 | AGDE01463 |
| DNA polymerase alpha subunit A | STCU04691 | AGDE08456 |
| DNA polymerase alpha subunit A | STCU05095 | nd |
| DNA polymerase alpha subunit B | nd | AGDE06815 |
| DNA polymerase alpha subunit B | nd | AGDE08096 |
| DNA polymerase alpha subunit B | nd | AGDE09843 |
| DNA polymerase delta subunit 1 | STCU00262 | AGDE00323 |
| DNA polymerase delta subunit 1 | STCU06184 | nd |
| DNA polymerase delta subunit 2 | STCU00158 | AGDE07520 |
| DNA polymerase delta subunit 2 | STCU06556 | nd |
| DNA polymerase delta subunit 2 | STCU08379 | nd |
| DNA polymerase epsilon catalytic subunit | STCU07806 | AGDE12104 |
| DNA polymerase epsilon subunit 2 | STCU06882 | AGDE05407 |
| DNA polymerase epsilon subunit B | nd | AGDE06617 |
| DNA polymerase I | STCU09553 | nd |
| DNA polymerase I alpha catalytic subunit | nd | AGDE05749 |
| DNA primase large subunit | STCU04939 | AGDE02301 |
| DNA primase large subunit | STCU05882 | AGDE10375 |
| DNA primase large subunit | nd | AGDE11007 |
| minichromosome maintenance (MCM) complex subunit | nd | AGDE12816 |
| minichromosome maintenance protein 3 | STCU05271 | AGDE10438 |
| minichromosome maintenance protein 3 | nd | AGDE11220 |
| minichromosome maintenance protein 4 (cell division control protein 54) | nd | AGDE07645 |
| minichromosome maintenance protein 4 (cell division control protein 54) | nd | AGDE08789 |
| minichromosome maintenance protein 5 (cell division control protein 46) | nd | AGDE05202 |
| minichromosome maintenance protein 5 (cell division control protein 46) | nd | AGDE06098 |
| minichromosome maintenance protein 7 (cell division control protein 47) | STCU09383 | AGDE01609 |
| minichromosome maintenance protein 7 (cell division control protein 47) | nd | AGDE01710 |
| minichromosome maintenance protein 7 (cell division control protein 47) | nd | AGDE10890 |
| minichromosome maintenance protein 7 (cell division control protein 47) | nd | AGDE12461 |
| mitochondrial DNA polymerase I protein A | nd | AGDE06910 |
| mitochondrial DNA polymerase I protein B | nd | AGDE03953 |
| proliferating cell nuclear antigen | STCU01681 | AGDE01481 |
| proliferating cell nuclear antigen | STCU07524 | AGDE00291 |
| proliferating cell nuclear antigen | STCU07930 | AGDE02121 |
| proliferating cell nuclear antigen | nd | AGDE08212 |
| replication factor A1 | STCU01732 | AGDE05315 |
| replication factor A1 | STCU03955 | AGDE06254 |
| replication factor A1 | STCU05268 | nd |
| replication factor C subunit 1 | nd | AGDE10103 |
| replication factor C subunit 2/4 | STCU00023 | AGDE00031 |
| replication factor C subunit 2/4 | STCU00039 | AGDE03270 |
| replication factor C subunit 2/4 | STCU02169 | AGDE04229 |
| replication factor C subunit 2/4 | STCU05947 | AGDE08344 |
| replication factor C subunit 2/4 | STCU07830 | nd |
| replication factor C subunit 2/4 | STCU08154 | nd |
| replication factor C subunit 3/5 | STCU07061 | AGDE03539 |
| replication factor C subunit 3/5 | STCU09811 | AGDE06019 |
| replication factor C subunit 3/5 | STCU09869 | nd |
| ribonuclease H2 subunit A | STCU00397 | AGDE00968 |
| ribonuclease H2 subunit A | nd | AGDE03666 |
| ribonuclease H2 subunit A | nd | AGDE04237 |
| ribonuclease H2 subunit A | nd | AGDE05820 |
| ribonuclease H2 subunit B | nd | AGDE08161 |
| ribonuclease HI | nd | AGDE02070 |
| ribonuclease HI | nd | AGDE07148 |
| ribonuclease HI | nd | AGDE07685 |
| ribonuclease HI | nd | AGDE11535 |
| ribonuclease HII | STCU07647 | AGDE00106 |
| ribonuclease HII | nd | AGDE08062 |
| ribonuclease HII | nd | AGDE10651 |
| single-strand DNA-binding protein | nd | AGDE17219 |
| **Base excision repair** | | |
| AP endonuclease 1 | nd | AGDE02118 |
| AP endonuclease 1 | nd | AGDE09062 |
| AP endonuclease 1 | nd | AGDE10637 |
| DNA ligase | STCU01088 | AGDE00030 |
| DNA ligase | nd | AGDE07027 |
| DNA ligase | nd | AGDE07405 |
| DNA ligase | nd | AGDE10322 |
| DNA ligase (ATP) | STCU00688 | AGDE02430 |
| DNA ligase (ATP) | STCU01920 | AGDE03121 |
| DNA ligase (ATP) | STCU05865 | AGDE05962 |
| DNA ligase (ATP) | STCU06213 | nd |
| DNA ligase (ATP) | STCU09744 | nd |
| DNA ligase 1 | STCU05790 | AGDE01463 |
| DNA ligase 1 | nd | AGDE06621 |
| DNA ligase 1 | nd | AGDE07592 |
| DNA ligase 1 | nd | AGDE08329 |
| DNA ligase I | STCU00307 | nd |
| DNA ligase K alpha | STCU00838 | nd |
| DNA polymerase beta subunit | STCU00334 | AGDE00408 |
| DNA polymerase beta subunit | nd | AGDE02662 |
| DNA polymerase beta subunit | nd | AGDE09058 |
| DNA polymerase delta subunit 1 | STCU00262 | AGDE00323 |
| DNA polymerase delta subunit 1 | STCU06184 | nd |
| DNA polymerase delta subunit 2 | STCU00158 | AGDE07520 |
| DNA polymerase delta subunit 2 | STCU06556 | nd |
| DNA polymerase delta subunit 2 | STCU08379 | nd |
| DNA polymerase epsilon catalytic subunit | STCU07806 | AGDE12104 |
| DNA polymerase epsilon subunit 2 | STCU06882 | AGDE05407 |
| DNA polymerase epsilon subunit B | nd | AGDE06617 |
| DNA polymerase I | STCU09553 | nd |
| endonuclease III | STCU00196 | nd |
| endonuclease III | STCU02039 | nd |
| endonuclease III | STCU02395 | nd |
| mitochondrial DNA polymerase beta | STCU02113 | nd |
| mitochondrial DNA polymerase beta-PAK | nd | AGDE06217 |
| mitochondrial DNA polymerase I protein A | nd | AGDE06910 |
| mitochondrial DNA polymerase I protein B | nd | AGDE03953 |
| N-glycosylase/DNA lyase | STCU04585 | nd |
| N-glycosylase/DNA lyase | STCU07684 | nd |
| N-glycosylase/DNA lyase | STCU08910 | nd |
| poly [ADP-ribose] polymerase | STCU05173 | AGDE11319 |
| poly [ADP-ribose] polymerase | STCU07850 | nd |
| poly [ADP-ribose] polymerase | STCU09095 | nd |
| proliferating cell nuclear antigen | STCU01681 | AGDE00291 |
| proliferating cell nuclear antigen | STCU07524 | AGDE01481 |
| proliferating cell nuclear antigen | STCU07930 | AGDE02121 |
| proliferating cell nuclear antigen | nd | AGDE08212 |
| uracil-DNA glycosylase | STCU01879 | AGDE02589 |
| uracil-DNA glycosylase | STCU02056 | AGDE04999 |
| uracil-DNA glycosylase | STCU02066 | AGDE11425 |
| uracil-DNA glycosylase | STCU04032 | AGDE11866 |
| uracil-DNA glycosylase | STCU06722 | AGDE17237 |
| **Mismatch repair** | | |
| DNA ligase | STCU01088 | AGDE00030 |
| DNA ligase | nd | AGDE07027 |
| DNA ligase | nd | AGDE07405 |
| DNA ligase | nd | AGDE10322 |
| DNA ligase (ATP) | STCU00688 | AGDE02430 |
| DNA ligase (ATP) | STCU01920 | AGDE03121 |
| DNA ligase (ATP) | STCU05865 | nd |
| DNA ligase (ATP) | STCU06213 | AGDE05962 |
| DNA ligase (ATP) | STCU09744 | nd |
| DNA ligase 1 | STCU05790 | AGDE06621 |
| DNA ligase 1 | nd | AGDE07592 |
| DNA ligase 1 | nd | AGDE08329 |
| DNA ligase I | STCU00307 | AGDE01463 |
| DNA ligase K alpha | STCU00838 | nd |
| DNA mismatch repair protein MLH1 | nd | AGDE14398 |
| DNA mismatch repair protein MSH2 | nd | AGDE02048 |
| DNA mismatch repair protein MutS | STCU07631 | nd |
| DNA mismatch repair protein MutS | STCU09735 | nd |
| DNA polymerase delta subunit 1 | STCU00262 | AGDE00323 |
| DNA polymerase delta subunit 1 | STCU06184 | nd |
| DNA polymerase delta subunit 2 | STCU00158 | AGDE07520 |
| DNA polymerase delta subunit 2 | STCU06556 | nd |
| DNA polymerase delta subunit 2 | STCU08379 | nd |
| proliferating cell nuclear antigen | STCU01681 | AGDE00291 |
| proliferating cell nuclear antigen | STCU07524 | AGDE01481 |
| proliferating cell nuclear antigen | STCU07930 | AGDE02121 |
| proliferating cell nuclear antigen | nd | AGDE08212 |
| replication factor A1 | STCU01732 | AGDE05315 |
| replication factor A1 | STCU03955 | AGDE06254 |
| replication factor A1 | STCU05268 | nd |
| replication factor C subunit 1 | nd | AGDE10103 |
| replication factor C subunit 2/4 | STCU00023 | AGDE00031 |
| replication factor C subunit 2/4 | STCU00039 | nd |
| replication factor C subunit 2/4 | STCU02169 | AGDE03270 |
| replication factor C subunit 2/4 | STCU05947 | AGDE04229 |
| replication factor C subunit 2/4 | STCU07830 | AGDE08344 |
| replication factor C subunit 2/4 | STCU08154 | nd |
| replication factor C subunit 3/5 | STCU07061 | AGDE03539 |
| replication factor C subunit 3/5 | STCU09811 | AGDE06019 |
| replication factor C subunit 3/5 | STCU09869 | nd |
| single-strand DNA-binding protein | nd | AGDE17219 |
| **Nucleotide excision repair** | | |
| DNA excision repair protein ERCC-1 | nd | AGDE06448 |
| DNA excision repair protein ERCC-2 | nd | AGDE07535 |
| DNA excision repair protein ERCC-2 | nd | AGDE09511 |
| DNA excision repair protein ERCC-5 | nd | AGDE07570 |
| DNA ligase | STCU01088 | AGDE00030 |
| DNA ligase | nd | AGDE07027 |
| DNA ligase | nd | AGDE07405 |
| DNA ligase | nd | AGDE10322 |
| DNA ligase (ATP) | STCU00688 | AGDE02430 |
| DNA ligase (ATP) | STCU01920 | AGDE03121 |
| DNA ligase (ATP) | STCU05865 | AGDE05962 |
| DNA ligase (ATP) | STCU06213 | nd |
| DNA ligase (ATP) | STCU09744 | nd |
| DNA ligase 1 | STCU05790 | AGDE06621 |
| DNA ligase I | STCU00307 | AGDE01463 |
| DNA ligase I | nd | AGDE07592 |
| DNA ligase I | nd | AGDE08329 |
| DNA ligase K alpha | STCU00838 | nd |
| DNA polymerase delta subunit 1 | STCU00262 | AGDE00323 |
| DNA polymerase delta subunit 1 | STCU06184 | nd |
| DNA polymerase delta subunit 2 | STCU00158 | AGDE07520 |
| DNA polymerase delta subunit 2 | STCU06556 | nd |
| DNA polymerase delta subunit 2 | STCU08379 | nd |
| DNA polymerase epsilon catalytic subunit | STCU07806 | AGDE12104 |
| DNA polymerase epsilon subunit 2 | STCU06882 | AGDE05407 |
| DNA polymerase epsilon subunit B | nd | AGDE06617 |
| DNA polymerase I | STCU09553 | nd |
| mitochondrial DNA polymerase I protein A | nd | AGDE06910 |
| mitochondrial DNA polymerase I protein B | nd | AGDE03953 |
| proliferating cell nuclear antigen | STCU01681 | AGDE00291 |
| proliferating cell nuclear antigen | STCU07524 | AGDE01481 |
| proliferating cell nuclear antigen | STCU07930 | AGDE02121 |
| proliferating cell nuclear antigen | nd | AGDE08212 |
| replication factor A1 | STCU01732 | AGDE05315 |
| replication factor A1 | STCU03955 | AGDE06254 |
| replication factor A1 | STCU05268 | nd |
| replication factor C subunit 1 | nd | AGDE10103 |
| replication factor C subunit 2/4 | STCU00023 | AGDE00031 |
| replication factor C subunit 2/4 | STCU00039 | AGDE03270 |
| replication factor C subunit 2/4 | STCU02169 | AGDE04229 |
| replication factor C subunit 2/4 | STCU05947 | AGDE08344 |
| replication factor C subunit 2/4 | STCU07830 | nd |
| replication factor C subunit 2/4 | STCU08154 | nd |
| replication factor C subunit 3/5 | STCU07061 | AGDE03539 |
| replication factor C subunit 3/5 | STCU09811 | AGDE06019 |
| replication factor C subunit 3/5 | STCU09869 | nd |
| RING-box protein 1 | nd | AGDE00024 |
| RING-box protein 1 | nd | AGDE00195 |
| RING-box protein 1 | nd | AGDE02183 |
| RING-box protein 1 | nd | AGDE05462 |
| transcription initiation factor TFIIH subunit 2 | nd | AGDE02465 |
| transcription initiation factor TFIIH subunit 2 | nd | AGDE03515 |
| transcription initiation factor TFIIH subunit 2 | nd | AGDE06208 |
| transcription initiation factor TFIIH subunit 4 | STCU06059 | AGDE11158 |
| UV excision repair protein RAD23 | STCU06711 | AGDE08421 |
| xeroderma pigmentosum group C-complementing protein | STCU04597 | nd |
| xeroderma pigmentosum group C-complementing protein | STCU07170 | nd |
| **Non-homologous end joining** | | |
| DNA ligase (ATP) | STCU00688 | AGDE02430 |
| DNA ligase K alpha | STCU00838 | nd |
| DNA ligase | STCU01088 | AGDE00030 |
| DNA ligase | nd | AGDE07027 |
| DNA ligase | nd | AGDE07405 |
| DNA ligase | nd | AGDE10322 |
| ATP-dependent DNA helicase 2 subunit 2 | STCU01330 | AGDE01690 |
| DNA ligase (ATP) | STCU01920 | AGDE03121 |
| DNA ligase (ATP) | STCU05865 | AGDE05962 |
| ATP-dependent DNA helicase 2 subunit 1 | STCU05895 | nd |
| DNA ligase (ATP) | STCU06213 | nd |
| DNA ligase (ATP) | STCU09744 | nd |
| DNA repair protein RAD50 | nd | AGDE12830 |
| DNA ligase 4 | nd | AGDE13074 |

nd: not determined
