# Supplementary material for: Predicting the Proteins of Angomonas deanei, Strigomonas culicis and Their Respective Endosymbionts Reveals New Aspects of the Trypanosomatidae Family
Source: PLoS One. 2013 Apr 3;8(4):e60209. doi: 10.1371/journal.pone.0060209 (PMC3616161; doi:10.1371/journal.pone.0060209)
Supplement: Table S10 — DNA replication and repair ORFs found in the A. deanei and S. culicis endosymbionts. (DOC) [file pone.0060209.s017.doc]

**Table S10**. DNA replication and repair ORFs found in the *A. deanei* and *S. culicis* endosymbionts.

|  | ***A. deanei* endosymbiont** | ***S. culicis* endosymbiont** |
| --- | --- | --- |
| ATP-dependent DNA helicase RecG | CKCE00253 | CKBE00469 |
| Crossover junction endodeoxyribonuclease RuvC | CKCE00062 | CKBE00652 |
| Chromosomal replication initiation protein | CKCE00573 | CKBE00167 |
| DNA helicase II / ATP-dependent DNA helicase PcrA | CKCE00367 | CKBE00361 |
| DNA ligase | CKCE00118 | CKBE00597 |
| DNA mismatch repair protein MutL | CKCE00047 | CKBE00670 |
| DNA mismatch repair protein MutS | CKCE00718 | CKBE00312 |
| DNA polymerase I | CKCE00192 | CKBE00523 |
| DNA polymerase III subunit alpha 1 | CKCE00022 | CKBE00696 |
| DNA polymerase III subunit beta | CKCE00574 | CKBE00168 |
| DNA polymerase III subunit epsilon | CKCE00417 | CKBE00012 |
| DNA polymerase III subunits gamma and tau | CKCE00702 | CKBE00299 |
| DNA primase 1 | CKCE00220 | CKBE00499 |
| DNA repair protein RecO (recombination protein O) | CKCE00146 | CKBE00569 |
| DNA-3-methyladenine glycosylase | CKCE00728 | CKBE00321 |
| Endonuclease III | CKCE00122 | CKBE00593 |
| Excinuclease A subunit A | CKCE00054 | CKBE00116 |
| Excinuclease ASTCU subunit B | CKCE00356 | CKBE00372 |
| Excinuclease ASTCU subunit C | CKCE00006 | CKBE00709 |
| Exodeoxyribonuclease III | CKCE00322 | CKBE00405/ CKBE00088 |
| Exodeoxyribonuclease VII large subunit | CKCE00316 | CKBE00411 |
| Exodeoxyribonuclease VII small subunit | CKCE00223 | CKBE00496 |
| Formamidopyrimidine-DNA glycosylase | CKCE00663 | CKBE00258 |
| Holliday junction DNA helicase RuvA | CKCE00061 | CKBE00653 |
| Holliday junction DNA helicase RuvB | CKCE00060 | CKBE00654 |
| Primosomal protein N' (replication factor Y) (superfamily II helicase) | CKCE00629 | CKBE00224 |
| Primosomal replication protein N | CKCE00228 | CKBE00491 |
| Recombination protein RecA | CKCE00396 | CKBE00334 |
| Recombination protein RecR | CKCE00704 | nd |
| Replicative DNA helicase | CKCE00231 | CKBE00488 |
| Ribonuclease HII | CKCE00272 | CKBE00453 |
| RNAse H | CKCE00418 | CKBE00013 |
| Single-strand DNA-binding protein | CKCE00519 | CKBE00115 |
| Single-stranded-DNA-specific exonuclease | CKCE00301 | CKBE00427 |
| Transcription-repair coupling factor (superfamily II helicase) | CKCE00112 | CKBE00604 |
| Uracil-DNA glycosylase | CKCE00497 | CKBE00090 |

nd: not determined
