# Supplementary material for: Predicting the Proteins of Angomonas deanei, Strigomonas culicis and Their Respective Endosymbionts Reveals New Aspects of the Trypanosomatidae Family
Source: PLoS One. 2013 Apr 3;8(4):e60209. doi: 10.1371/journal.pone.0060209 (PMC3616161; doi:10.1371/journal.pone.0060209)
Supplement: Table S11 — Identified ORFs involved in DNA transcription and RNA splicing in the genome of A. deanei and S. culicis. (DOC) [file pone.0060209.s018.doc]

**Table S11**. Identified ORFs involved in DNA transcription and RNA splicing in the genome of *A. deanei* and *S. culicis*.

|  | ***S. culicis*** | | ***A. deanei*** | |
| --- | --- | --- | --- | --- |
| **RNA polymerase** |  | |  | |
| DNA-directed RNA polymerases I, II, and III subunit RPASTCU3 | STCU00022 | | AGDE00199 | |
| DNA-directed RNA polymerases I, II, and III subunit RPASTCU3 | nd | | AGDE01243 | |
| DNA-directed RNA polymerases I, II, and III subunit RPASTCU3 | nd | | AGDE12197 | |
| DNA-directed RNA polymerase II subunit RPB7 | STCU00279 | | AGDE00989 | |
| DNA-directed RNA polymerase II subunit RPB7 | nd | | AGDE04642 | |
| DNA-directed RNA polymerase III subunit RPC10 | STCU00892 | | nd | |
| DNA-directed RNA polymerase III subunit RPC2 | STCU01149 | | nd | |
| DNA-directed RNA polymerase II subunit RPB11 | STCU01629 | | AGDE02057 | |
| DNA-directed RNA polymerase II subunit RPB11 | nd | | AGDE02253 | |
| DNA-directed RNA polymerase II subunit RPB3 | STCU01909 | | AGDE02421 | |
| DNA-directed RNA polymerase II subunit RPB3 | STCU02861 | | AGDE03600 | |
| DNA-directed RNA polymerase II subunit RPB3 | STCU07686 | | AGDE07466 | |
| DNA-directed RNA polymerase II subunit RPB3 | nd | | AGDE08432 | |
| DNA-directed RNA polymerase II subunit RPB3 | nd | | AGDE10051 | |
| DNA-directed RNA polymerase II subunit RPB3 | nd | | AGDE09142 | |
| DNA-directed RNA polymerase subunit beta | STCU03718 | | nd | |
| DNA-directed RNA polymerase I subunit RPA2 | STCU05720 | | AGDE08290 | |
| DNA-directed RNA polymerase I subunit RPA2 | nd | | AGDE10855 | |
| DNA-directed RNA polymerases I, II, and III subunit RPASTCU1 | STCU05766 | | nd | |
| DNA-directed RNA polymerases I, II, and III subunit RPASTCU2 | STCU06345 | | AGDE00547 | |
| DNA-directed RNA polymerases I, II, and III subunit RPASTCU2 | nd | | AGDE06614 | |
| DNA-directed RNA polymerase III subunit RPC1 | nd | | AGDE10786 | |
| DNA-directed RNA polymerase I largest subunit | STCU08378 | | AGDE08968 | |
| DNA-directed RNA polymerase I largest subunit | STCU09252 | | AGDE09100 | |
| DNA-directed RNA polymerase I largest subunit | STCU11667 | | nd | |
| DNA-directed RNA polymerases I, II, and III subunit RPASTCU5 | nd | | AGDE00225 | |
| DNA-directed RNA polymerase II subunit RPB9 | nd | | AGDE03846 | |
| DNA-directed RNA polymerase II subunit RPB9 | nd | | AGDE05198 | |
| DNA-directed RNA polymerase II subunit RPB9 | nd | | AGDE10646 | |
| DNA-directed RNA polymerase II subunit RPB2 | nd | | AGDE11096 | |
| DNA-directed RNA polymerase II subunit RPB2 | nd | | AGDE11125 | |
| DNA-directed RNA polymerase II subunit RPB2 | nd | | AGDE11981 | |
| DNA-directed RNA polymerase II subunit 2 | nd | | AGDE11930 | |
| **Basal transcription factors** | | | | |
| bifunctional inhibitor/lipid-transfer protein/seed storage 2S albumin-like protein | nd | | AGDE14813 | |
| DNA excision repair protein ERCC-2 | nd | | AGDE07535 | |
| DNA excision repair protein ERCC-2 | nd | | AGDE09511 | |
| transcription initiation factor TFIID subunit 9 | STCU01942 | | AGDE01839 | |
| transcription initiation factor TFIID subunit 9 | STCU05462 | | AGDE02459 | |
| transcription initiation factor TFIID subunit 9 | nd | | AGDE03095 | |
| transcription initiation factor TFIID subunit 9 | nd | | AGDE08323 | |
| transcription initiation factor TFIID subunit 9 | nd | | AGDE10604 | |
| transcription initiation factor TFIID TATA-box-binding protein | STCU04624 | | AGDE09842 | |
| transcription initiation factor TFIID TATA-box-binding protein | nd | | AGDE11763 | |
| transcription initiation factor TFIIH subunit 2 | nd | | AGDE02465 | |
| transcription initiation factor TFIIH subunit 2 | nd | | AGDE03515 | |
| transcription initiation factor TFIIH subunit 2 | nd | | AGDE06208 | |
| transcription initiation factor TFIIH subunit 4 | STCU06059 | | AGDE11158 | |
| **Spliceosome** | | | | |
| 70 kDa heat shock protein | STCU05132 | | nd | |
| ATP-dependent RNA helicase | STCU00320 | | AGDE07644 | |
| ATP-dependent RNA helicase DDX5/DBP2 | nd | | AGDE03490 | |
| ATP-dependent RNA helicase DDX5/DBP2 | nd | | AGDE06467 | |
| ATP-dependent RNA helicase DHX8/PRP22 | STCU08539 | | AGDE07489 | |
| ATP-dependent RNA helicase DHX8/PRP22 | STCU08890 | | nd | |
| ATP-dependent RNA helicase UAP56/SUB2 | STCU03230 | | AGDE04031 | |
| ATP-dependent RNA helicase UAP56/SUB2 | STCU05381 | | AGDE09579 | |
| ATP-dependent RNA helicase UAP56/SUB2 | STCU07885 | | AGDE10545 | |
| crooked neck | STCU07822 | | nd | |
| heat shock 70kDa protein 1/8 | STCU00047 | | AGDE02530 | |
| heat shock 70kDa protein 1/8 | STCU01506 | | AGDE02582 | |
| heat shock 70kDa protein 1/8 | STCU02819 | | AGDE02644 | |
| heat shock 70kDa protein 1/8 | STCU04931 | | AGDE05796 | |
| heat shock 70kDa protein 1/8 | nd | | AGDE07279 | |
| heat shock 70kDa protein 1/8 | nd | | AGDE11013 | |
| heat shock protein 70 | STCU02051 | | AGDE07263 | |
| heat shock protein 70 | STCU04560 | | AGDE07701 | |
| heat-shock protein hsp70 | nd | | AGDE03499 | |
| heat-shock protein hsp70 | nd | | AGDE06512 | |
| heat-shock protein hsp70 | nd | | AGDE12033 | |
| nuclear cap-binding protein subunit 2 | STCU00056 | | AGDE08910 | |
| PHD finger-like domain-containing protein 5A | STCU03221 | | nd | |
| pleiotropic regulator 1 | STCU00658 | | AGDE04512 | |
| pleiotropic regulator 1 | STCU08691 | | AGDE09141 | |
| pre-mRNA splicing factor ATP-dependent RNA helicase | nd | | AGDE01291 | |
| pre-mRNA-processing factor 19 | STCU05327 | | nd | |
| pre-mRNA-processing factor SLU7 | nd | | AGDE06365 | |
| pre-mRNA-processing factor SLU7 | nd | | AGDE06791 | |
| pre-mRNA-processing factor SLU7 | nd | | AGDE09519 | |
| pre-mRNA-splicing factor ATP-dependent RNA helicase DHX15/PRP43 | nd | | AGDE10527 | |
| pre-mRNA-splicing factor ISY1 | STCU05174 | | nd | |
| pre-mRNA-splicing factor SYF1 | nd | | AGDE08192 | |
| protein mago nashi | STCU00988 | | AGDE02128 | |
| protein mago nashi | STCU03193 | | AGDE09185 | |
| protein mago nashi | STCU07376 | | AGDE09335 | |
| small nuclear ribonucleoprotein B and B' | STCU03899 | | AGDE03001 | |
| small nuclear ribonucleoprotein B and B' | nd | | AGDE06187 | |
| small nuclear ribonucleoprotein D2 | nd | | AGDE01085 | |
| small nuclear ribonucleoprotein D3 | STCU00316 | | AGDE01478 | |
| small nuclear ribonucleoprotein D3 | STCU01161 | | AGDE05701 | |
| small nuclear ribonucleoprotein E | STCU04296 | | AGDE02466 | |
| small nuclear ribonucleoprotein E | nd | | AGDE01024 | |
| small nuclear ribonucleoprotein E | nd | | AGDE07068 | |
| small nuclear ribonucleoprotein F | STCU04441 | | nd | |
| small nuclear ribonucleoprotein F | STCU06017 | | nd | |
| splicing factor 3A subunit 3 | STCU04996 | | AGDE00263 | |
| splicing factor 3A subunit 3 | nd | | AGDE07857 | |
| splicing factor 3B subunit 1 | STCU02764 | | AGDE03439 | |
| splicing factor 3B subunit 1 | STCU08663 | | AGDE06218 | |
| splicing factor 3B subunit 1 | nd | | AGDE10611 | |
| splicing factor 3B subunit 1 | nd | | AGDE11407 | |
| splicing factor 3B subunit 3 | nd | | AGDE09938 | |
| splicing factor 3B subunit 5 | STCU03053 | | nd | |
| splicing factor U2AF 35 kDa subunit | STCU00086 | | nd | |
| U4/U6 small nuclear ribonucleoprotein PRP31 | STCU01626 | | AGDE06516 | |
| U4/U6 small nuclear ribonucleoprotein PRP31 | STCU02459 | | AGDE08582 | |
| U4/U6 small nuclear ribonucleoprotein PRP31 | STCU04699 | | AGDE10845 | |
| U4/U6 small nuclear ribonucleoprotein SNU13 | nd | | AGDE00088 | |
| U4/U6 small nuclear ribonucleoprotein SNU13 | nd | | AGDE02207 | |
| U4/U6 small nuclear ribonucleoprotein SNU13 | nd | | AGDE05870 | |
| U5 snRNP protein, DIM1 family | STCU05417 | | nd | |
| U5 snRNP protein, DIM1 family | STCU08640 | | nd | |
| U6 snRNA-associated Sm-like protein LSm7 | nd | | AGDE00270 | |
| U6 snRNA-associated Sm-like protein LSm7 | nd | | AGDE06067 | |
|  | |  | |  |

nd: not determined
