# Supplementary material for: Predicting the Proteins of Angomonas deanei, Strigomonas culicis and Their Respective Endosymbionts Reveals New Aspects of the Trypanosomatidae Family
Source: PLoS One. 2013 Apr 3;8(4):e60209. doi: 10.1371/journal.pone.0060209 (PMC3616161; doi:10.1371/journal.pone.0060209)
Supplement: Table S12 — Transcription related proteins in the endosymbionts of A. deanei and S. culicis. (DOC) [file pone.0060209.s019.doc]

***Table S12****. Transcription related proteins in the endosymbionts of A. deanei and S. culicis.*

| **Endosymbiont ORF** | **Functional Classification** | **Score** | **E-value** | **Identity** | **Positivity** | **Organism** |
| --- | --- | --- | --- | --- | --- | --- |
| CKCE00530 | DNA-directed RNA polymerase subunit alpha | 524.00 | 1,00E-147 | 98.47% | 97.87% | *B. petrii* |
| CKCE00560 | DNA-directed RNA polymerase subunit beta' | 2472.00 | 0 | 98.49% | 96.96% | *B. bronchiseptica* |
| CKCE00561 | DNA-directed RNA polymerase subunit beta | 2317.00 | 0 | 100% | 99.42% | *B. avium* |
| CKCE00222 | RNA polymerase sigma factor RpoD | 950.00 | 0 | 87.23% | 85.79% | *B. parapertussis* |
| CKCE00605 | RNA polymerase sigma-32 factor | 373.00 | 1,00E-101 | 91.61% | 88.60% | *B. pertussis* |
| CKCE00112 | transcription-repair coupling factor | 1310.00 | 0 | 96.62% | 97.38% | *B. avium* |
| CKBE00125 | DNA-directed RNA polymerase subunit alpha | 528.00 | 1,00E-148 | 100% | 99.39% | *B. avium* |
| CKBE00154 | DNA-directed RNA polymerase subunit beta | 2475.00 | 0 | 99.78% | 98.30% | *B. bronchiseptica* |
| CKBE00155 | DNA-directed RNA polymerase subunit beta | 2261.00 | 0 | 100.00% | 96.57% | *B. petrii* |
| CKBE00498 | RNA polymerase sigma factor RpoD | 980.00 | 0 | 88.62% | 86.05% | *B. parapertussis* |
| CKBE00199 | RNA polymerase sigma-32 factor | 390.00 | 1,00E-107 | 95.92% | 96.26% | *A. xylosoxidans* |
| CKBE00604 | transcription-repair coupling factor | 1277.00 | 0 | 98.70% | 98.27% | *A. xylosoxidans* |
