# Supplementary material for: Predicting the Proteins of Angomonas deanei, Strigomonas culicis and Their Respective Endosymbionts Reveals New Aspects of the Trypanosomatidae Family
Source: PLoS One. 2013 Apr 3;8(4):e60209. doi: 10.1371/journal.pone.0060209 (PMC3616161; doi:10.1371/journal.pone.0060209)
Supplement: Table S13 — Main ORFs detected participating in ribosomal biogenesis and translation in A. deanei and S. culicis. (DOC) [file pone.0060209.s020.doc]

**Table S13**. Main ORFs detected participating in ribosomal biogenesis and translation *in A. deanei* and *S. culicis.*

|  | ***S. culicis*** | ***A. deanei*** |
| --- | --- | --- |
| **Ribosome biogenesis in eukaryotes** |  |  |
| 5'-3' exoribonuclease 1 | STCU08047 | AGDE00610 |
| 5'-3' exoribonuclease 2 | STCU03318 | AGDE07010 |
| 5'-3' exoribonuclease 2 | nd | AGDE09501 |
| 5'-3' exoribonuclease 2 | nd | AGDE11884 |
| casein kinase II subunit alpha | STCU03897 | AGDE00510 |
| casein kinase II subunit alpha | STCU11089 | AGDE01769 |
| casein kinase II subunit alpha | nd | AGDE02504 |
| casein kinase II subunit alpha | nd | AGDE04853 |
| casein kinase II subunit alpha | nd | AGDE07653 |
| casein kinase II subunit beta | STCU03185 | AGDE00186 |
| casein kinase II subunit beta | nd | AGDE03974 |
| casein kinase II subunit beta | nd | AGDE09877 |
| essential for mitotic growth 1 | STCU01476 | AGDE02412 |
| essential for mitotic growth 1 | STCU07340 | AGDE09051 |
| essential for mitotic growth 1 | STCU08457 | AGDE10502 |
| Eukaryotic translation initiation factor 6 | nd | AGDE04461 |
| exportin-1 | STCU00690 | AGDE06290 |
| exportin-1 | STCU05215 | AGDE08626 |
| exportin-1 | STCU08796 | nd |
| exportin-1 | STCU09148 | nd |
| GTP-binding protein nuclear protein Ran | STCU00791 | AGDE01866 |
| GTP-binding protein nuclear protein Ran | STCU05338 | AGDE03668 |
| GTP-binding protein nuclear protein Ran | STCU06744 | nd |
| H/ACA ribonucleoprotein complex subunit 1 | nd | AGDE12753 |
| H/ACA ribonucleoprotein complex subunit 2 | STCU00492 | AGDE00589 |
| H/ACA ribonucleoprotein complex subunit 2 | nd | AGDE03912 |
| H/ACA ribonucleoprotein complex subunit 2 | STCU03138 | AGDE03024 |
| H/ACA ribonucleoprotein complex subunit 2 | nd | AGDE08785 |
| H/ACA ribonucleoprotein complex subunit 3 | STCU04070 | nd |
| H/ACA ribonucleoprotein complex subunit 4 | STCU03418 | AGDE04288 |
| H/ACA ribonucleoprotein complex subunit 4 | STCU09429 | AGDE06076 |
| H/ACA ribonucleoprotein complex subunit 4 | STCU09809 | AGDE10039 |
| large subunit GTPase 1 | nd | AGDE05009 |
| Midasin | STCU09891 | AGDE13625 |
| N-acetyltransferase 10 | STCU08651 | AGDE09464 |
| N-acetyltransferase 10 | nd | AGDE11714 |
| N-acetyltransferase 10 | nd | AGDE12405 |
| NET1-associated nuclear protein 1 (U3 small nucleolar RNA-associated protein 17) | nd | AGDE06369 |
| NET1-associated nuclear protein 1 (U3 small nucleolar RNA-associated protein 17) | nd | AGDE09714 |
| NTF2-related export protein 1/2 | nd | AGDE04637 |
| nuclear GTP-binding protein | STCU00366 | AGDE00477 |
| nuclear GTP-binding protein | STCU08706 | AGDE02218 |
| nuclear GTP-binding protein | STCU09398 | AGDE05910 |
| nuclear GTP-binding protein | STCU09677 | AGDE06814 |
| nuclear RNA export factor 1/2 | nd | AGDE11481 |
| nuclear RNA export factor 1/2 | nd | AGDE12004 |
| nuclear RNA export factor ½ | nd | AGDE00055 |
| nucleolar GTP-binding protein | STCU00392 | AGDE06623 |
| nucleolar GTP-binding protein | STCU06504 | AGDE08730 |
| nucleolar GTP-binding protein | STCU06646 | nd |
| nucleolar protein 4 | STCU02486 | AGDE07102 |
| nucleolar protein 4 | STCU06637 | AGDE07651 |
| nucleolar protein 4 | STCU06638 | nd |
| nucleolar protein 56 | STCU09196 | AGDE06554 |
| nucleolar protein 58 | STCU01635 | AGDE07236 |
| nucleolar protein 58 | STCU02882 | AGDE08884 |
| nucleolar protein 58 | STCU08172 | nd |
| periodic tryptophan protein 2 | nd | AGDE11975 |
| periodic tryptophan protein 2 | nd | AGDE12329 |
| ribosome biogenesis ATPase | STCU09525 | nd |
| ribosome biogenesis ATPase | STCU09612 | nd |
| ribosome biogenesis protein BMS1 | nd | AGDE11435 |
| ribosome maturation protein SDO1 | nd | AGDE13061 |
| RIO kinase 1 | STCU07672 | AGDE02583 |
| RIO kinase 1 | STCU08853 | nd |
| RIO kinase 1 | nd | AGDE09598 |
| RNA 3'-terminal phosphate cyclase-like protein | nd | AGDE02158 |
| RNA-binding protein NOB1 | STCU01329 | AGDE08586 |
| RNA-binding protein NOB1 | STCU05828 | nd |
| RNA-binding protein NOB1 | STCU08747 | nd |
| rRNA 2'-O-methyltransferase fibrillarin | nd | AGDE01351 |
| rRNA 2'-O-methyltransferase fibrillarin | nd | AGDE04531 |
| rRNA 2'-O-methyltransferase fibrillarin | nd | AGDE06562 |
| rRNA 2'-O-methyltransferase fibrillarin | nd | AGDE07113 |
| rRNA 2'-O-methyltransferase fibrillarin | nd | AGDE10620 |
| transcription initiation factor TFIID subunit 9 | STCU01942 | AGDE01839 |
| transcription initiation factor TFIID subunit 9 | STCU05462 | AGDE02459 |
| transcription initiation factor TFIID subunit 9 | nd | AGDE03095 |
| transcription initiation factor TFIID subunit 9 | nd | AGDE08323 |
| transcription initiation factor TFIID subunit 9 | nd | AGDE10604 |
| translation initiation factor 6 | STCU01209 | nd |
| U3 small nucleolar ribonucleoprotein IMP3 | STCU01452 | AGDE00167 |
| U3 small nucleolar ribonucleoprotein IMP3 | STCU05491 | AGDE00345 |
| U3 small nucleolar ribonucleoprotein IMP3 | nd | AGDE03351 |
| U3 small nucleolar ribonucleoprotein IMP3 | nd | AGDE05675 |
| U3 small nucleolar ribonucleoprotein IMP3 | nd | AGDE06578 |
| U3 small nucleolar ribonucleoprotein IMP4 | STCU03490 | AGDE03002 |
| U3 small nucleolar ribonucleoprotein IMP4 | STCU05075 | AGDE09871 |
| U3 small nucleolar RNA-associated protein 12 | STCU07880 | AGDE16052 |
| U3 small nucleolar RNA-associated protein 13 | STCU08005 | AGDE04805 |
| U3 small nucleolar RNA-associated protein 13 | STCU09027 | nd |
| U3 small nucleolar RNA-associated protein 14 | STCU06948 | nd |
| U3 small nucleolar RNA-associated protein 14 | STCU09302 | nd |
| U3 small nucleolar RNA-associated protein 15 | STCU05511 | AGDE05292 |
| U3 small nucleolar RNA-associated protein 15 | STCU08812 | AGDE06337 |
| U3 small nucleolar RNA-associated protein 15 | STCU09238 | AGDE07840 |
| U3 small nucleolar RNA-associated protein 15 | nd | AGDE10099 |
| U3 small nucleolar RNA-associated protein 18 | nd | AGDE06801 |
| U3 small nucleolar RNA-associated protein 21 | nd | AGDE06464 |
| U3 small nucleolar RNA-associated protein 21 | nd | AGDE11468 |
| U3 small nucleolar RNA-associated protein 24 | STCU00288 | AGDE00357 |
| U3 small nucleolar RNA-associated protein 24 | STCU00993 | AGDE05228 |
| U3 small nucleolar RNA-associated protein 24 | STCU01187 | AGDE09826 |
| U3 small nucleolar RNA-associated protein 24 | STCU04141 | AGDE11364 |
| U3 small nucleolar RNA-associated protein MPP10 | STCU04013 | nd |
| U3 small nucleolar RNA-associated protein MPP10 | STCU09113 | nd |
| U4/U6 small nuclear ribonucleoprotein SNU13 | nd | AGDE00088 |
| U4/U6 small nuclear ribonucleoprotein SNU13 | nd | AGDE02207 |
| U4/U6 small nuclear ribonucleoprotein SNU13 | nd | AGDE05870 |
| **Ribosome** | | |
| 60S ribosomal protein L4 | nd | AGDE05706 |
| 60S ribosomal protein L6 | nd | AGDE06825 |
| large subunit ribosomal protein L10 | nd | AGDE05496 |
| large subunit ribosomal protein L10Ae | STCU00001 | AGDE00184 |
| large subunit ribosomal protein L10Ae | nd | AGDE03211 |
| large subunit ribosomal protein L10Ae | nd | AGDE04638 |
| large subunit ribosomal protein L10Ae | nd | AGDE05418 |
| large subunit ribosomal protein L10Ae | nd | AGDE05724 |
| large subunit ribosomal protein L10Ae | nd | AGDE06108 |
| large subunit ribosomal protein L10Ae | nd | AGDE06703 |
| large subunit ribosomal protein L10Ae | nd | AGDE07515 |
| large subunit ribosomal protein L10e | nd | AGDE02523 |
| large subunit ribosomal protein L10e | nd | AGDE03165 |
| large subunit ribosomal protein L10e | nd | AGDE03542 |
| large subunit ribosomal protein L10e | nd | AGDE10961 |
| large subunit ribosomal protein L11e | nd | AGDE01298 |
| large subunit ribosomal protein L11e | nd | AGDE07979 |
| large subunit ribosomal protein L11e | nd | AGDE09866 |
| large subunit ribosomal protein L11e | nd | AGDE10571 |
| large subunit ribosomal protein L12e | STCU01738 | AGDE05178 |
| large subunit ribosomal protein L12e | STCU04144 | AGDE07812 |
| large subunit ribosomal protein L12e | nd | AGDE11697 |
| large subunit ribosomal protein L13 | STCU04217 | AGDE05288 |
| large subunit ribosomal protein L13 | STCU05310 | AGDE05610 |
| large subunit ribosomal protein L13 | nd | AGDE06099 |
| large subunit ribosomal protein L13 | nd | AGDE11887 |
| large subunit ribosomal protein L13Ae | STCU00349 | AGDE00426 |
| large subunit ribosomal protein L13Ae | STCU00608 | AGDE03217 |
| large subunit ribosomal protein L13Ae | STCU02367 | AGDE03266 |
| large subunit ribosomal protein L13Ae | STCU05290 | AGDE04610 |
| large subunit ribosomal protein L13Ae | STCU06952 | AGDE07381 |
| large subunit ribosomal protein L13Ae | STCU08442 | AGDE11907 |
| large subunit ribosomal protein L13e | STCU02181 | AGDE00302 |
| large subunit ribosomal protein L13e | STCU03468 | AGDE02750 |
| large subunit ribosomal protein L13e | STCU09651 | AGDE02946 |
| large subunit ribosomal protein L13e | nd | AGDE04905 |
| large subunit ribosomal protein L13e | nd | AGDE06935 |
| large subunit ribosomal protein L13e | nd | AGDE07038 |
| large subunit ribosomal protein L13e | nd | AGDE12256 |
| large subunit ribosomal protein L14 | STCU02136 | AGDE02698 |
| large subunit ribosomal protein L14 | STCU02934 | nd |
| large subunit ribosomal protein L14 | STCU03852 | nd |
| large subunit ribosomal protein L14e | STCU08478 | AGDE03102 |
| large subunit ribosomal protein L14e | STCU08670 | AGDE03247 |
| large subunit ribosomal protein L14e | nd | AGDE04156 |
| large subunit ribosomal protein L14e | nd | AGDE05698 |
| large subunit ribosomal protein L14e | nd | AGDE10946 |
| large subunit ribosomal protein L15e | nd | AGDE03715 |
| large subunit ribosomal protein L15e | nd | AGDE05365 |
| large subunit ribosomal protein L15e | nd | AGDE11132 |
| large subunit ribosomal protein L17 | nd | AGDE00935 |
| large subunit ribosomal protein L17 | nd | AGDE02654 |
| large subunit ribosomal protein L17e | STCU01239 | AGDE01946 |
| large subunit ribosomal protein L17e | STCU01537 | AGDE02977 |
| large subunit ribosomal protein L17e | STCU06395 | nd |
| large subunit ribosomal protein L18Ae | STCU00569 | AGDE01537 |
| large subunit ribosomal protein L18Ae | STCU01716 | AGDE02170 |
| large subunit ribosomal protein L18Ae | STCU02952 | AGDE03452 |
| large subunit ribosomal protein L18Ae | nd | AGDE03670 |
| large subunit ribosomal protein L18Ae | nd | AGDE07738 |
| large subunit ribosomal protein L18e | STCU00481 | AGDE00579 |
| large subunit ribosomal protein L18e | STCU06125 | AGDE00668 |
| large subunit ribosomal protein L18e | STCU06475 | AGDE02274 |
| large subunit ribosomal protein L18e | nd | AGDE11477 |
| large subunit ribosomal protein L19e | STCU01657 | AGDE05973 |
| large subunit ribosomal protein L19e | STCU02046 | nd |
| large subunit ribosomal protein L2 | STCU05308 | nd |
| large subunit ribosomal protein L21e | nd | AGDE05285 |
| large subunit ribosomal protein L21e | nd | AGDE06342 |
| large subunit ribosomal protein L22e | nd | AGDE01837 |
| large subunit ribosomal protein L22e | nd | AGDE03173 |
| large subunit ribosomal protein L22e | nd | AGDE06092 |
| large subunit ribosomal protein L23Ae | STCU06707 | AGDE03119 |
| large subunit ribosomal protein L23Ae | STCU09558 | AGDE09085 |
| large subunit ribosomal protein L23e | STCU02361 | AGDE07372 |
| large subunit ribosomal protein L23e | STCU02629 | AGDE07918 |
| large subunit ribosomal protein L24e | STCU01128 | AGDE01796 |
| large subunit ribosomal protein L24e | STCU03222 | AGDE02271 |
| large subunit ribosomal protein L24e | STCU07130 | AGDE02659 |
| large subunit ribosomal protein L24e | nd | AGDE07109 |
| large subunit ribosomal protein L24e | nd | AGDE09493 |
| large subunit ribosomal protein L26e | STCU03617 | AGDE03484 |
| large subunit ribosomal protein L26e | STCU06434 | AGDE04500 |
| large subunit ribosomal protein L26e | nd | AGDE12179 |
| large subunit ribosomal protein L27Ae | STCU03834 | AGDE02059 |
| large subunit ribosomal protein L27Ae | STCU07036 | AGDE06789 |
| large subunit ribosomal protein L27Ae | STCU07439 | AGDE06944 |
| large subunit ribosomal protein L27Ae | STCU08072 | AGDE08533 |
| large subunit ribosomal protein L27e | STCU01771 | AGDE04671 |
| large subunit ribosomal protein L27e | STCU03749 | nd |
| large subunit ribosomal protein L27e | STCU07386 | nd |
| large subunit ribosomal protein L28e | STCU02713 | nd |
| large subunit ribosomal protein L29e | STCU01638 | nd |
| large subunit ribosomal protein L29e | STCU02875 | nd |
| large subunit ribosomal protein L29e | STCU03108 | nd |
| large subunit ribosomal protein L30e | STCU01633 | AGDE06282 |
| large subunit ribosomal protein L30e | STCU01663 | AGDE10858 |
| large subunit ribosomal protein L30e | STCU01814 | AGDE10912 |
| large subunit ribosomal protein L30e | nd | AGDE11370 |
| large subunit ribosomal protein L32e | STCU00613 | AGDE00738 |
| large subunit ribosomal protein L32e | STCU01539 | AGDE07018 |
| large subunit ribosomal protein L32e | STCU01822 | AGDE11278 |
| large subunit ribosomal protein L32e | STCU07311 | nd |
| large subunit ribosomal protein L34e | nd | AGDE06023 |
| large subunit ribosomal protein L34e | nd | AGDE06779 |
| large subunit ribosomal protein L34e | nd | AGDE06819 |
| large subunit ribosomal protein L34e | nd | AGDE08445 |
| large subunit ribosomal protein L34e | nd | AGDE11056 |
| large subunit ribosomal protein L34e | nd | AGDE11221 |
| large subunit ribosomal protein L35Ae | STCU03075 | AGDE03645 |
| large subunit ribosomal protein L35Ae | STCU04018 | AGDE06236 |
| large subunit ribosomal protein L35Ae | STCU06122 | AGDE11059 |
| large subunit ribosomal protein L35e | nd | AGDE01227 |
| large subunit ribosomal protein L35e | nd | AGDE02815 |
| large subunit ribosomal protein L35e | nd | AGDE03172 |
| large subunit ribosomal protein L35e | nd | AGDE06335 |
| large subunit ribosomal protein L36e | STCU03626 | nd |
| large subunit ribosomal protein L36e | STCU04336 | nd |
| large subunit ribosomal protein L38e | nd | AGDE01543 |
| large subunit ribosomal protein L38e | nd | AGDE05997 |
| large subunit ribosomal protein L38e | nd | AGDE06341 |
| large subunit ribosomal protein L38e | nd | AGDE03451 |
| large subunit ribosomal protein L3e | STCU00869 | AGDE01097 |
| large subunit ribosomal protein L3e | nd | AGDE02329 |
| large subunit ribosomal protein L3e | nd | AGDE02776 |
| large subunit ribosomal protein L3e | nd | AGDE04131 |
| large subunit ribosomal protein L3e | nd | AGDE06727 |
| large subunit ribosomal protein L3e | nd | AGDE10368 |
| large subunit ribosomal protein L3e | nd | AGDE11953 |
| large subunit ribosomal protein L3e | nd | AGDE12412 |
| large subunit ribosomal protein L40e | nd | AGDE04499 |
| large subunit ribosomal protein L44e | STCU01658 | AGDE02092 |
| large subunit ribosomal protein L44e | STCU07118 | AGDE06802 |
| large subunit ribosomal protein L44e | nd | AGDE07632 |
| large subunit ribosomal protein L4e | STCU07430 | AGDE00229 |
| large subunit ribosomal protein L4e | STCU08807 | AGDE06596 |
| large subunit ribosomal protein L4e | STCU09044 | AGDE06829 |
| large subunit ribosomal protein L4e | nd | AGDE07859 |
| large subunit ribosomal protein L5e | STCU00924 | AGDE01051 |
| large subunit ribosomal protein L5e | STCU00965 | AGDE01163 |
| large subunit ribosomal protein L5e | STCU02905 | AGDE01216 |
| large subunit ribosomal protein L5e | STCU09240 | AGDE03406 |
| large subunit ribosomal protein L5e | nd | AGDE03599 |
| large subunit ribosomal protein L5e | nd | AGDE09147 |
| large subunit ribosomal protein L5e | nd | AGDE10807 |
| large subunit ribosomal protein L6e | STCU01112 | AGDE00355 |
| large subunit ribosomal protein L6e | STCU02293 | AGDE00888 |
| large subunit ribosomal protein L6e | STCU03208 | AGDE01402 |
| large subunit ribosomal protein L6e | STCU04902 | AGDE02904 |
| large subunit ribosomal protein L6e | STCU05159 | AGDE06770 |
| large subunit ribosomal protein L6e | nd | AGDE08709 |
| large subunit ribosomal protein L7e | STCU01253 | AGDE03813 |
| large subunit ribosomal protein L7e | STCU01695 | AGDE01022 |
| large subunit ribosomal protein L7e | STCU06296 | AGDE11886 |
| large subunit ribosomal protein L9e | STCU01975 | nd |
| large subunit ribosomal protein LP0 | nd | AGDE08485 |
| large subunit ribosomal protein LP1 | STCU02692 | AGDE06127 |
| large subunit ribosomal protein LP1 | STCU04732 | AGDE06656 |
| large subunit ribosomal protein LP1 | STCU04955 | AGDE08054 |
| large subunit ribosomal protein LP1 | STCU05571 | nd |
| large subunit ribosomal protein LP2 | nd | AGDE01508 |
| large subunit ribosomal protein LP2 | nd | AGDE04196 |
| large subunit ribosomal protein LP2 | nd | AGDE06336 |
| large subunit ribosomal protein LP2 | nd | AGDE09585 |
| small subunit ribosomal protein S10 | STCU00290 | nd |
| small subunit ribosomal protein S10 | STCU04171 | nd |
| small subunit ribosomal protein S10e | nd | AGDE11094 |
| small subunit ribosomal protein S11 | STCU02187 | nd |
| small subunit ribosomal protein S11e | STCU00574 | AGDE00684 |
| small subunit ribosomal protein S11e | STCU02001 | AGDE02533 |
| small subunit ribosomal protein S11e | STCU03262 | AGDE04616 |
| small subunit ribosomal protein S11e | STCU05126 | nd |
| small subunit ribosomal protein S12 | STCU12405 | nd |
| small subunit ribosomal protein S12 | STCU12411 | nd |
| small subunit ribosomal protein S12e | STCU00095 | AGDE00554 |
| small subunit ribosomal protein S12e | STCU00399 | AGDE08102 |
| small subunit ribosomal protein S13e | STCU12237 | AGDE02314 |
| small subunit ribosomal protein S13e | nd | AGDE02357 |
| small subunit ribosomal protein S14e | STCU00888 | AGDE12420 |
| small subunit ribosomal protein S14e | nd | AGDE01255 |
| small subunit ribosomal protein S14e | nd | AGDE01511 |
| small subunit ribosomal protein S14e | nd | AGDE02761 |
| small subunit ribosomal protein S14e | nd | AGDE05982 |
| small subunit ribosomal protein S14e | nd | AGDE11430 |
| small subunit ribosomal protein S15e | STCU02035 | AGDE02567 |
| small subunit ribosomal protein S15e | STCU02410 | AGDE03052 |
| small subunit ribosomal protein S15e | STCU05539 | AGDE03376 |
| small subunit ribosomal protein S15e | nd | AGDE09018 |
| small subunit ribosomal protein S16e | STCU00816 | AGDE01655 |
| small subunit ribosomal protein S16e | STCU03301 | AGDE04120 |
| small subunit ribosomal protein S16e | STCU05939 | AGDE05955 |
| small subunit ribosomal protein S16e | nd | AGDE08467 |
| small subunit ribosomal protein S16e | nd | AGDE10060 |
| small subunit ribosomal protein S17e | STCU03900 | AGDE05681 |
| small subunit ribosomal protein S17e | STCU09160 | AGDE10805 |
| small subunit ribosomal protein S19e | STCU04222 | AGDE05906 |
| small subunit ribosomal protein S19e | nd | AGDE06622 |
| small subunit ribosomal protein S19e | nd | AGDE09512 |
| small subunit ribosomal protein S19e | nd | AGDE09676 |
| small subunit ribosomal protein S19e | nd | AGDE09813 |
| small subunit ribosomal protein S19e | nd | AGDE11601 |
| small subunit ribosomal protein S20e | STCU01668 | AGDE05221 |
| small subunit ribosomal protein S20e | STCU04380 | AGDE05482 |
| small subunit ribosomal protein S23e | STCU01576 | AGDE01995 |
| small subunit ribosomal protein S23e | STCU04739 | AGDE09976 |
| small subunit ribosomal protein S23e | nd | AGDE12439 |
| small subunit ribosomal protein S24e | STCU01702 | nd |
| small subunit ribosomal protein S24e | STCU02295 | nd |
| small subunit ribosomal protein S24e | STCU02707 | nd |
| small subunit ribosomal protein S24e | STCU09611 | nd |
| small subunit ribosomal protein S25e | STCU01490 | AGDE02851 |
| small subunit ribosomal protein S25e | STCU04111 | AGDE05107 |
| small subunit ribosomal protein S25e | nd | AGDE10551 |
| small subunit ribosomal protein S26e | STCU00456 | AGDE00560 |
| small subunit ribosomal protein S26e | STCU00702 | AGDE04377 |
| small subunit ribosomal protein S26e | nd | AGDE06121 |
| small subunit ribosomal protein S26e | nd | AGDE08507 |
| small subunit ribosomal protein S26e | nd | AGDE08675 |
| small subunit ribosomal protein S27e | nd | AGDE02152 |
| small subunit ribosomal protein S27e | nd | AGDE03822 |
| small subunit ribosomal protein S28e | nd | AGDE12685 |
| small subunit ribosomal protein S2e | STCU02277 | AGDE01808 |
| small subunit ribosomal protein S2e | STCU05578 | AGDE05835 |
| small subunit ribosomal protein S2e | STCU05900 | nd |
| small subunit ribosomal protein S2e | STCU07216 | nd |
| small subunit ribosomal protein S2e | STCU08367 | nd |
| small subunit ribosomal protein S30e | STCU00215 | nd |
| small subunit ribosomal protein S30e | STCU00373 | nd |
| small subunit ribosomal protein S30e | STCU01220 | nd |
| small subunit ribosomal protein S3Ae | STCU00914 | nd |
| small subunit ribosomal protein S3Ae | STCU04005 | nd |
| small subunit ribosomal protein S3Ae | STCU05122 | nd |
| small subunit ribosomal protein S3Ae | STCU08767 | nd |
| small subunit ribosomal protein S3e | STCU04041 | AGDE05013 |
| small subunit ribosomal protein S3e | STCU08844 | AGDE00654 |
| small subunit ribosomal protein S3e | nd | AGDE06493 |
| small subunit ribosomal protein S3e | nd | AGDE07261 |
| small subunit ribosomal protein S3e | nd | AGDE12324 |
| small subunit ribosomal protein S4e | STCU01876 | AGDE02594 |
| small subunit ribosomal protein S4e | STCU03000 | AGDE08782 |
| small subunit ribosomal protein S4e | nd | AGDE10211 |
| small subunit ribosomal protein S4e | nd | AGDE11110 |
| small subunit ribosomal protein S5e | STCU00501 | AGDE00981 |
| small subunit ribosomal protein S5e | STCU03088 | AGDE03857 |
| small subunit ribosomal protein S5e | STCU04980 | nd |
| small subunit ribosomal protein S5e | STCU07571 | nd |
| small subunit ribosomal protein S5e | STCU09491 | nd |
| small subunit ribosomal protein S6e | nd | AGDE03144 |
| small subunit ribosomal protein S6e | nd | AGDE05316 |
| small subunit ribosomal protein S6e | nd | AGDE12468 |
| small subunit ribosomal protein S7e | STCU02892 | AGDE00455 |
| small subunit ribosomal protein S7e | nd | AGDE02496 |
| small subunit ribosomal protein S7e | nd | AGDE03583 |
| small subunit ribosomal protein S7e | nd | AGDE03773 |
| small subunit ribosomal protein S7e | nd | AGDE04740 |
| small subunit ribosomal protein S7e | nd | AGDE05516 |
| small subunit ribosomal protein S7e | nd | AGDE10340 |
| small subunit ribosomal protein S7e | nd | AGDE12189 |
| small subunit ribosomal protein S8e | STCU00798 | AGDE00022 |
| small subunit ribosomal protein S8e | STCU01020 | AGDE00993 |
| small subunit ribosomal protein S8e | STCU03046 | AGDE01268 |
| small subunit ribosomal protein S8e | STCU04584 | AGDE03038 |
| small subunit ribosomal protein S8e | STCU04829 | AGDE08631 |
| small subunit ribosomal protein S9e | STCU02123 | AGDE02676 |
| small subunit ribosomal protein S9e | STCU02392 | AGDE03025 |
| small subunit ribosomal protein S9e | STCU06856 | AGDE05224 |
| small subunit ribosomal protein S9e | STCU07012 | AGDE06373 |
| small subunit ribosomal protein SAe | STCU00127 | AGDE00012 |
| small subunit ribosomal protein SAe | STCU01223 | AGDE00159 |
| small subunit ribosomal protein SAe | STCU02131 | AGDE01558 |
| small subunit ribosomal protein SAe | STCU04565 | AGDE03364 |
| small subunit ribosomal protein SAe | STCU08361 | AGDE03785 |
| small subunit ribosomal protein SAe | nd | AGDE05436 |
| small subunit ribosomal protein SAe | nd | AGDE08628 |
| small subunit ribosomal protein SAe | nd | AGDE09388 |
| small subunit ribosomal protein SAe | nd | AGDE09874 |
| **Aminoacyl-tRNA biosynthesis** | | |
| alanyl-tRNA synthetase | STCU08782 | AGDE08761 |
| alanyl-tRNA synthetase | STCU09829 | nd |
| arginyl-tRNA synthetase | STCU01561 | AGDE02758 |
| arginyl-tRNA synthetase | STCU09277 | AGDE05998 |
| asparaginyl-tRNA synthetase | STCU05260 | nd |
| asparaginyl-tRNA synthetase | STCU07870 | nd |
| aspartyl-tRNA synthetase | STCU01918 | AGDE01336 |
| aspartyl-tRNA synthetase | STCU04263 | AGDE05347 |
| aspartyl-tRNA synthetase | STCU05456 | AGDE06193 |
| aspartyl-tRNA synthetase | STCU08322 | AGDE07981 |
| aspartyl-tRNA synthetase | nd | AGDE11520 |
| cysteinyl-tRNA synthetase | STCU08342 | AGDE11415 |
| glutaminyl-tRNA synthetase | STCU01842 | AGDE03887 |
| glutaminyl-tRNA synthetase | STCU04846 | AGDE04654 |
| glutaminyl-tRNA synthetase | STCU08199 | AGDE08529 |
| glutaminyl-tRNA synthetase | STCU08447 | nd |
| glutaminyl-tRNA synthetase | STCU09662 | nd |
| glutamyl-tRNA synthetase | STCU07079 | AGDE04557 |
| glutamyl-tRNA synthetase | nd | AGDE07362 |
| glutamyl-tRNA synthetase | nd | AGDE08970 |
| glutamyl-tRNA synthetase | nd | AGDE09973 |
| glycyl tRNA synthetase | STCU08082 | AGDE06298 |
| glycyl tRNA synthetase | nd | AGDE10248 |
| glycyl-tRNA synthetase | STCU01869 | AGDE08065 |
| histidyl-tRNA synthetase | STCU01471 | AGDE01867 |
| histidyl-tRNA synthetase | STCU06698 | AGDE03920 |
| histidyl-tRNA synthetase | nd | AGDE06120 |
| histidyl-tRNA synthetase | nd | AGDE07418 |
| isoleucyl-tRNA synthetase | STCU04733 | AGDE01578 |
| isoleucyl-tRNA synthetase | STCU08045 | AGDE08231 |
| isoleucyl-tRNA synthetase | STCU09165 | nd |
| leucyl-tRNA synthetase | STCU00149 | AGDE02510 |
| leucyl-tRNA synthetase | STCU06674 | AGDE12326 |
| lysyl-tRNA synthetase | STCU02547 | AGDE03248 |
| lysyl-tRNA synthetase | nd | AGDE07763 |
| lysyl-tRNA synthetase, class II | STCU04491 | AGDE06156 |
| lysyl-tRNA synthetase, class II | STCU05152 | AGDE07180 |
| lysyl-tRNA synthetase, class II | STCU08976 | AGDE08255 |
| lysyl-tRNA synthetase, class II | STCU09597 | AGDE09225 |
| lysyl-tRNA synthetase, class II | nd | AGDE10613 |
| lysyl-tRNA synthetase, class II | nd | AGDE11725 |
| methionyl-tRNA synthetase | nd | AGDE07310 |
| methionyl-tRNA synthetase | nd | AGDE07896 |
| O-phospho-L-seryl-tRNASec:L-selenocysteinyl-tRNA synthase | nd | AGDE11808 |
| phenylalanyl-tRNA synthetase alpha chain | STCU07565 | AGDE00639 |
| phenylalanyl-tRNA synthetase alpha chain | nd | AGDE08604 |
| phenylalanyl-tRNA synthetase alpha chain | nd | AGDE11585 |
| phenylalanyl-tRNA synthetase beta chain | STCU07879 | AGDE06220 |
| phenylalanyl-tRNA synthetase beta chain | nd | AGDE06521 |
| prolyl-tRNA synthetase | STCU05837 | AGDE07474 |
| prolyl-tRNA synthetase | nd | AGDE09697 |
| seryl-tRNA synthetase | STCU00182 | AGDE02342 |
| seryl-tRNA synthetase | STCU06295 | AGDE03224 |
| seryl-tRNA synthetase | STCU07349 | AGDE07576 |
| seryl-tRNA synthetase | STCU07399 | AGDE12258 |
| seryl-tRNA synthetase | STCU07881 | AGDE12320 |
| seryl-tRNA synthetase | STCU08958 | nd |
| seryl-tRNA synthetase | STCU09690 | nd |
| threonyl-tRNA synthetase | STCU06282 | AGDE03934 |
| threonyl-tRNA synthetase | nd | AGDE04067 |
| tryptophanyl-tRNA synthetase | STCU00544 | AGDE00109 |
| tryptophanyl-tRNA synthetase | STCU04742 | AGDE00653 |
| tryptophanyl-tRNA synthetase | STCU05010 | AGDE12309 |
| tryptophanyl-tRNA synthetase | STCU07481 | AGDE12427 |
| tyrosyl-tRNA synthetase | STCU06786 | AGDE09847 |
| tyrosyl-tRNA synthetase | nd | AGDE06757 |
| tyrosyl-tRNA synthetase | nd | AGDE10139 |
| tyrosyl-tRNA synthetase | nd | AGDE11918 |
| valyl-tRNA synthetase | STCU01881 | AGDE02384 |
| valyl-tRNA synthetase | STCU04310 | AGDE11666 |
| valyl-tRNA synthetase | STCU07749 | AGDE11838 |
| valyl-tRNA synthetase | STCU08795 | nd |
| valyl-tRNA synthetase | STCU09227 | nd |

nd: not determined
