# Supplementary material for: Predicting the Proteins of Angomonas deanei, Strigomonas culicis and Their Respective Endosymbionts Reveals New Aspects of the Trypanosomatidae Family
Source: PLoS One. 2013 Apr 3;8(4):e60209. doi: 10.1371/journal.pone.0060209 (PMC3616161; doi:10.1371/journal.pone.0060209)
Supplement: Table S14 — Identified phosphatases in A. deanei and S. culicis. (DOC) [file pone.0060209.s021.doc]

**Table S14.** Identified phosphatases in *A. deanei* and *S. culicis.*

|  | ***A. deanei*** | **Family** |
| --- | --- | --- |
| dual specificity protein phosphatase or MAP kinase phosphatase | AGDE01195 | PTPc |
| polynucleotide kinase 3'-phosphatase Partial | AGDE03035 | crotonase-like |
| polynucleotide kinase 3'-phosphatase | AGDE03519 | P-loop NTPase/HAD-like |
| polynucleotide kinase 3'-phosphatase | AGDE03724 | P-loop NTPase/HAD-like |
| polynucleotide kinase 3'-phosphatase | AGDE11414 | P-loop NTPase/HAD-like |
| putative 6-phosphofructo-2-kinase/fructose-2,6-biphosphatase | AGDE15923 | Phosphoglycerate mutase-like |
| polynucleotide kinase 3'-phosphatase | AGDE06333 | P-loop NTPase |
| 6-phosphofructo-2-kinase/fructose-2,6-biphosphatase | AGDE15922 | ANK |
| dual specificity protein phosphatase or MAP kinase phosphatase | AGDE05008 | PTPc |
|  |  |  |
|  | ***S. culicis*** | **Family** |
| 6-phosphofructo-2-kinase/fructose-2,6-biphosphatase-1-like protein | STCU01968 | P-loop NTPase/HP |
| polynucleotide kinase 3'-phosphatase | STCU02835 | P-loop NTPase/HAD-like |
| 6-phosphofructo-2-kinase/fructose-2,6-biphosphatase-1-like protein | STCU05284 | P-loop NTPase/HP/ANK |
| 6-phosphofructo-2-kinase/fructose-2,6-biphosphatase-1-like protein | STCU07002 | P-loop NTPase/HP/ANK |
| 6-phosphofructo-2-kinase/fructose-2,6-biphosphatase-1-like protein | STCU08258 | P-loop NTPase/HP/ANK |
| polynucleotide kinase 3'-phosphatase | STCU09243 | P-loop NTPase/HAD-like |
| dual specificity protein phosphatase or MAP kinase phosphatase | STCU12164 | PTPc |
