# Supplementary material for: Predicting the Proteins of Angomonas deanei, Strigomonas culicis and Their Respective Endosymbionts Reveals New Aspects of the Trypanosomatidae Family
Source: PLoS One. 2013 Apr 3;8(4):e60209. doi: 10.1371/journal.pone.0060209 (PMC3616161; doi:10.1371/journal.pone.0060209)
Supplement: Table S15 — Number of heat shock and stress response proteins in A. deanei and S.culicis. (DOC) [file pone.0060209.s022.doc]

**Table S15.** Number of heat shock and stress response proteins in *A. deanei* and S. *culicis*

|  | ***A. deanei*** | ***S. culicis*** |
| --- | --- | --- |
| **Heat stress** |  |  |
| HSP10 | 4 | 3 |
| HSP100 | 2 | 1 |
| HSP20 | 1 | 1 |
| HSP40 | 100 | 67 |
| HSP60 | 14 | 12 |
| HSP70 | 15 | 20 |
| HSP78 | 0 | 1 |
| HSP85 | 3 | 4 |
| **Nutritional stress** |  |  |
| Autophagin | 0 | 0 |
| DEAD box helicase DHH1 | 37 | 24 |
| STI1 | 6 | 8 |
| **Osmotic stress** |  |  |
| Acidocalcisomal exopolyphosphatase | 5 | 4 |
| Phosphatidylinositol 3-kinase | 10 | 11 |
| **Oxidative stress** |  |  |
| orbate peroxidase | 3 | 3 |
| Fe–Superoxide dismutase | 13 | 13 |
| Glutathione peroxidase | 9 | 7 |
| Methionine sulfoxide reductase | 3 | 2 |
| TcG6PDH | 3 | 2 |
| Trypanothione reductase | 3 | 3 |
| Trypanothione synthetase | 11 | 6 |
| Tryparedoxin | 12 | 5 |
| Tryparedoxin peroxidase | 15 | 10 |
| Cytochrome P450 reductases | 2 | 2 |
