# Supplementary material for: Predicting the Proteins of Angomonas deanei, Strigomonas culicis and Their Respective Endosymbionts Reveals New Aspects of the Trypanosomatidae Family
Source: PLoS One. 2013 Apr 3;8(4):e60209. doi: 10.1371/journal.pone.0060209 (PMC3616161; doi:10.1371/journal.pone.0060209)
Supplement: Table S16 — Glycerophospholipids (GPL) enzymes of A. deanei and S. culicis1. (DOC) [file pone.0060209.s023.doc]

**Table S16**. Glycerophospholipids (GPs) enzymes of *A. deanei* and *S. culicis^1^.*

| **Enzyme/subunit** | **Entry name** | **E.C.** | ***A. deanei*** | ***S. culicis*** |  |
| --- | --- | --- | --- | --- | --- |
| Glycerol-3-phosphate dehydrogenase [NAD+], glycosomal/mitochondrial | LmjF10.0510 | 1.1.1.8 | AGDE01125 | STCU06258 | |
| Glycerol-3-phosphate dehydrogenase-like protein | LmjF20.0430 | 1.1.5.3 | AGDE04238 | STCU09594 | |
| Glycerol-3-phosphate acyltransferase | LmjF03.0080 | 2.3.1.15 | AGDE06732 | STCU03095 | |
| 1-acyl-sn-glycerol-3-phosphateacyltransferase-like protein | LbrM32_V2.2150 | 2.3.1.51 | AGDE03085 | nd | |
| Actyltransferase-like protein | LINJ_13_0500 | 2.3.1.- | AGDE00543 | nd | |
| CDP-diacylglycerol--inositol 3-phosphatidyltransferase | LmjF26.2480 | 2.7.8.11 | AGDE03561 | STCU03768 | |
| Phosphatidate cytidylyltransferase | LmjF26.1620 | 2.7.7.41 | AGDE10077 AGDE09922 | STCU01286 | |
| Phosphatidylglycerophosphate synthase | LmjF07.0200 | 2.7.8.5 | AGDE04934 | STCU09094 | |
| Cardiolipin synthetase | LINJ_34_1860 | 2.7.8.- | AGDE07340 | nd | |
| Phosphatase methylesterase 1 | LINJ_32_0960 | 3.1.1.- | AGDE05167 | STCU00964 | |
| Phosphatidylserine synthase | LINJ_14_1280 | 2.7.8.8 | AGDE01754 | nd | |
| Phosphatidylserine decarboxylase | LmjF35.4590 | 4.1.1.65 | AGDE02302 | nd | |
| Phosphatidylserine synthase 2 | XP_001687720.1 | 2.7.8.29 | AGDE08045 AGDE01754 | nd | |
| Diacylglycerol kinase | LmjF35.5370 | 2.7.1.107 | AGDE02361 | nd | |
| Phosphatidate phosphatase | LmjF06.0830 | 3.1.3.4 | AGDE12093 | nd | |
| Phosphatidylethanolamine n-methyltransferase | LmjF31.2290 | 2.1.1.17 | AGDE07839 | STCU03869 | |
| ethanolaminephosphotransferase | LmjF36.5900 | 2.7.8.1 | AGDE09394 | STCU03637 | |
| Lysophospholipase | LmjF24.1840 | 3.1.1.5 | AGDE00627 | nd | |
| Ethanolamine-phosphate cytidylyltransferase | LmjF32.0890 | 2.7.7.14 | AGDE00483 | nd | |
| Glycerophosphoryl diester phosphodiesterase | LmjF36.5960 | 3.1.4.46 | AGDE05793 | STCU01003 | |

^1^ GPs are important membrane components and its metabolism is interconnected in a way that the depletion or absence of one enzyme or even one pathway may be compensated by the activity of an enzyme of a different pathway, as the synthesis of PC by Kennedy pathway or Greenberg pathway. The Greenberg pathway was not found in the proteome of both protozoa, despite one of the two enzymes are present in *A. deanei* proteome. None of the proteomes presents Kennedy pathway enzymes; although *A. deanei* proteome presented some sequences of low coverage (choline kinase, cholinephosphate cytidylyltransferase and choline/ethanolamine phosphate transferase).

nd: not determined
