# Supplementary material for: Predicting the Proteins of Angomonas deanei, Strigomonas culicis and Their Respective Endosymbionts Reveals New Aspects of the Trypanosomatidae Family
Source: PLoS One. 2013 Apr 3;8(4):e60209. doi: 10.1371/journal.pone.0060209 (PMC3616161; doi:10.1371/journal.pone.0060209)
Supplement: Table S17 — Glycerophospholipids (GPL) enzymes of A. deanei and S. culicis endosymbionts. (DOC) [file pone.0060209.s024.doc]

**Table S17**. Glycerophospholipids (GPs) enzymes of *A. deanei and* *S. culicis* endosymbionts.

| **Enzyme/subunit** | **Entry name** | **E.C.** | ***A. deanei* endosymbiont** | ***S. culicis* endosymbiont** |
| --- | --- | --- | --- | --- |
| NAD(P)H-dependent glycerol-3-phosphate dehydrogenase | BP0603 | 1.1.1.94 | CKCE00461 | CKBE00054 |
| glycerol-3-phosphate acyltransferase PlsX | BP1718 | 2.3.1.15 | CKCE00135 | CKBE00580 |
| glycerol-3-phosphate acyltransferase PlsY |  | 2.3.1.15 | nd | CKBE00389 |
| 1-acyl-sn-glycerol-3-phosphate acyltransferase | BP0036 | 2.3.1.51 | CKCE00038 | CKBE00679 |
| phosphatidate cytidylyltransferase | BP1424 | 2.7.7.41 | nd | CKBE00445 |
| CDP-diacylglycerol--glycerol-3-phosphate 3-phosphatidyltransferase | BPP1766 | 2.7.8.5 | CKCE00007 | CKBE00708 |
| phosphatidylglycerophosphatase | BPP0861 | 3.1.3.27 | CKCE00326 | CKBE00400 |
| CDP-diacylglycerol--serine O-phosphatidyltransferase | BPP3434 | 2.7.8.8 | CKCE00091 | nd |
| phosphatidylserine decarboxylase | BPP2924 | 4.1.1.65 | CKCE00255 | nd |

nd: not determined
