# Supplementary material for: Predicting the Proteins of Angomonas deanei, Strigomonas culicis and Their Respective Endosymbionts Reveals New Aspects of the Trypanosomatidae Family
Source: PLoS One. 2013 Apr 3;8(4):e60209. doi: 10.1371/journal.pone.0060209 (PMC3616161; doi:10.1371/journal.pone.0060209)
Supplement: Table S18 — Ectonucleotidases families and identification of ORFs found in A. dean ei and S. culicis . (DOC) [file pone.0060209.s025.doc]

**Table S18.** Ectonucleotidases families and identification of ORFs found in *A. dean*ei and *S. culicis*.

| **FAMILY** | **ID Pfam** | **EC NUMBER** | **CONSERVED PATTERN/ID PROSITE** | **OBSERVED PATTERN IN *A. deanei/S. culicis*** | **ORF *A. deanei*** | **ORF *S. culicis*** |
| --- | --- | --- | --- | --- | --- | --- |
|  |  |  | **ACR1** (D-CAIG-G-S-STN-GHRGSAK-STN-RSTQK)* |  | AGDE08613 AGDE10787 |  |
|  |  |  | **ACR2** (PGASFL-LVTIMAF-YFHVMIETALNR-ILVMF-LGKFYCNRQA-CASG-T-AG-G-MLVI-R)* |  |  |  |
| ENTPDases | PF06079 | 3.6.1.5 | **ACR3** (GASF-KQSTVYWRCHADNEF-QEADVYT-EG-GA-VAILP-YFLSM-AGDL-WF-IVL-GTSAH-IVATL-NQ)* | GRDEGMFSWLTLN/GDAEGLYSWVALN |  | STCU09614 |
|  |  |  | **ACR4** (DE-MLIFV-G-GR-VAGS-S-TLFVAMS-Q)* | DMGGASSQ/DMGGASSQ |  |  |
|  |  |  | **ACR5** (W-TASCQHP-LIVDHR-G)* | WPLG/WPLG |  |  |
|  |  |  |  |  |  |  |
| CD73 | PF05761 | 3.1.3.5 | **Pattern 1** [LIVM]-x-[LIVM](2)-[HEA]-[TI]-x-D-x-H-[GSA]-x-[LIVMF) / (PS00785) |  |  |  |
|  |  |  | **Pattern 2** [FYPH]-x(4)-[LIVM]-G-N-H-E-F-[DN] / (PS00786) |  |  |  |
|  |  |  |  |  |  |  |
| ADA | PF00962 | 3.5.4.4 | [SA]-[LIVM]-[NGS]-[STA]-D-D-P (PS00485) | SLSTDDP/SLSTDDP | AGDE12975 | STCU09343 |

* The sequence of “apyrase conserved regions” (ACR1 to ACR5) was extracted from Sansom et al., 2008.
