# Supplementary material for: Predicting the Proteins of Angomonas deanei, Strigomonas culicis and Their Respective Endosymbionts Reveals New Aspects of the Trypanosomatidae Family
Source: PLoS One. 2013 Apr 3;8(4):e60209. doi: 10.1371/journal.pone.0060209 (PMC3616161; doi:10.1371/journal.pone.0060209)
Supplement: Table S19 — ORFs encoding enzymes involved in purine and pyrimidine metabolism of A. deanei , S. culicis and their symbionts. (DOC) [file pone.0060209.s026.doc]

**Table S19.** ORFs encoding enzymes involved in purine and pyrimidine metabolism of *A. deanei*, *S. culicis* and their symbionts.

| **Trypanosomatid** | | **Endosymbiont** | | **EC** | **Gene name** |
| --- | --- | --- | --- | --- | --- |
| ***A. deanei*** | ***S. culicis*** | ***A. deanei*** | ***S. culicis*** |
| AGDE00095 AGDE08376 AGDE10264 AGDE06487 AGDE08651 AGDE10906 AGDE06738 AGDE09095 AGDE01941 | STCU05852 STCU08865 STCU08443 STCU08479 STCU06180 |  |  |  | Solute carrier Family 29 (equilibrative nucleoside transporte), member 1/2/3 |
| AGDE00665 AGDE03693 AGDE16707 | STCU00351 STCU03926 STCU00553 STCU02967 |  |  | EC:2.4.2.7 | adenine phosphoribosyltransferase |
| AGDE01909 AGDE10661 | [STCU01505](http://ligeirinha.lncc.br/bc-bin/annotation/annotation.cgi?id=0&gene=BC01505) [STCU04670](http://ligeirinha.lncc.br/bc-bin/annotation/annotation.cgi?id=0&gene=BC04670) [STCU07055](http://ligeirinha.lncc.br/bc-bin/annotation/annotation.cgi?id=0&gene=BC07055) |  |  | EC 2.4.2.8 | hypoxanthine-guanine phosphoribosyltransferase |
| [AGDE00150](http://ligeirinha.lncc.br/cd-bin/annotation/annotation.cgi?id=0&gene=CD00150) [AGDE00188](http://ligeirinha.lncc.br/cd-bin/annotation/annotation.cgi?id=0&gene=CD00188) [AGDE00276](http://ligeirinha.lncc.br/cd-bin/annotation/annotation.cgi?id=0&gene=CD00276) [AGDE00548](http://ligeirinha.lncc.br/cd-bin/annotation/annotation.cgi?id=0&gene=CD00548) [AGDE00662](http://ligeirinha.lncc.br/cd-bin/annotation/annotation.cgi?id=0&gene=CD00662) [AGDE01070](http://ligeirinha.lncc.br/cd-bin/annotation/annotation.cgi?id=0&gene=CD01070) [AGDE01214](http://ligeirinha.lncc.br/cd-bin/annotation/annotation.cgi?id=0&gene=CD01214) [AGDE01765](http://ligeirinha.lncc.br/cd-bin/annotation/annotation.cgi?id=0&gene=CD01765) [AGDE01839](http://ligeirinha.lncc.br/cd-bin/annotation/annotation.cgi?id=0&gene=CD01839) [AGDE02148](http://ligeirinha.lncc.br/cd-bin/annotation/annotation.cgi?id=0&gene=CD02148) [AGDE02459](http://ligeirinha.lncc.br/cd-bin/annotation/annotation.cgi?id=0&gene=CD02459) [AGDE03095](http://ligeirinha.lncc.br/cd-bin/annotation/annotation.cgi?id=0&gene=CD03095) [AGDE03555](http://ligeirinha.lncc.br/cd-bin/annotation/annotation.cgi?id=0&gene=CD03555) [AGDE04049](http://ligeirinha.lncc.br/cd-bin/annotation/annotation.cgi?id=0&gene=CD04049) [AGDE04539](http://ligeirinha.lncc.br/cd-bin/annotation/annotation.cgi?id=0&gene=CD04539) [AGDE05032](http://ligeirinha.lncc.br/cd-bin/annotation/annotation.cgi?id=0&gene=CD05032) [AGDE05329](http://ligeirinha.lncc.br/cd-bin/annotation/annotation.cgi?id=0&gene=CD05329) [AGDE05718](http://ligeirinha.lncc.br/cd-bin/annotation/annotation.cgi?id=0&gene=CD05718) [AGDE06631](http://ligeirinha.lncc.br/cd-bin/annotation/annotation.cgi?id=0&gene=CD06631)[AGDE06856](http://ligeirinha.lncc.br/cd-bin/annotation/annotation.cgi?id=0&gene=CD06856) [AGDE07226](http://ligeirinha.lncc.br/cd-bin/annotation/annotation.cgi?id=0&gene=CD07226) [AGDE08323](http://ligeirinha.lncc.br/cd-bin/annotation/annotation.cgi?id=0&gene=CD08323) [AGDE08640](http://ligeirinha.lncc.br/cd-bin/annotation/annotation.cgi?id=0&gene=CD08640)[AGDE10604](http://ligeirinha.lncc.br/cd-bin/annotation/annotation.cgi?id=0&gene=CD10604) [AGDE10950](http://ligeirinha.lncc.br/cd-bin/annotation/annotation.cgi?id=0&gene=CD10950) [AGDE11837](http://ligeirinha.lncc.br/cd-bin/annotation/annotation.cgi?id=0&gene=CD11837) [AGDE12385](http://ligeirinha.lncc.br/cd-bin/annotation/annotation.cgi?id=0&gene=CD12385) | [STCU00013](http://ligeirinha.lncc.br/bc-bin/annotation/annotation.cgi?id=0&gene=BC00013) [STCU00221](http://ligeirinha.lncc.br/bc-bin/annotation/annotation.cgi?id=0&gene=BC00221) [STCU00863](http://ligeirinha.lncc.br/bc-bin/annotation/annotation.cgi?id=0&gene=BC00863) [STCU00962](http://ligeirinha.lncc.br/bc-bin/annotation/annotation.cgi?id=0&gene=BC00962) [STCU01390](http://ligeirinha.lncc.br/bc-bin/annotation/annotation.cgi?id=0&gene=BC01390) [STCU01455](http://ligeirinha.lncc.br/bc-bin/annotation/annotation.cgi?id=0&gene=BC01455) [STCU01942](http://ligeirinha.lncc.br/bc-bin/annotation/annotation.cgi?id=0&gene=BC01942) [STCU02334](http://ligeirinha.lncc.br/bc-bin/annotation/annotation.cgi?id=0&gene=BC02334) [STCU02821](http://ligeirinha.lncc.br/bc-bin/annotation/annotation.cgi?id=0&gene=BC02821) [STCU03885](http://ligeirinha.lncc.br/bc-bin/annotation/annotation.cgi?id=0&gene=BC03885) [STCU04195](http://ligeirinha.lncc.br/bc-bin/annotation/annotation.cgi?id=0&gene=BC04195) [STCU04514](http://ligeirinha.lncc.br/bc-bin/annotation/annotation.cgi?id=0&gene=BC04514) [STCU04544](http://ligeirinha.lncc.br/bc-bin/annotation/annotation.cgi?id=0&gene=BC04544) [STCU04571](http://ligeirinha.lncc.br/bc-bin/annotation/annotation.cgi?id=0&gene=BC04571) [STCU04804](http://ligeirinha.lncc.br/bc-bin/annotation/annotation.cgi?id=0&gene=BC04804) [STCU04875](http://ligeirinha.lncc.br/bc-bin/annotation/annotation.cgi?id=0&gene=BC04875) [STCU05462](http://ligeirinha.lncc.br/bc-bin/annotation/annotation.cgi?id=0&gene=BC05462) [STCU05760](http://ligeirinha.lncc.br/bc-bin/annotation/annotation.cgi?id=0&gene=BC05760) [STCU05818](http://ligeirinha.lncc.br/bc-bin/annotation/annotation.cgi?id=0&gene=BC05818) [STCU07100](http://ligeirinha.lncc.br/bc-bin/annotation/annotation.cgi?id=0&gene=BC07100) [STCU08743](http://ligeirinha.lncc.br/bc-bin/annotation/annotation.cgi?id=0&gene=BC08743) |  |  | EC 2.7.4.3 | adenylate kinase |
|  | [STCU08089](http://ligeirinha.lncc.br/bc-bin/annotation/annotation.cgi?id=0&gene=BC08089) [STCU09343](http://ligeirinha.lncc.br/bc-bin/annotation/annotation.cgi?id=0&gene=BC09343) [STCU09710](http://ligeirinha.lncc.br/bc-bin/annotation/annotation.cgi?id=0&gene=BC09710)A |  |  | EC:3.5.4.6 | AMP deaminase |
| [AGDE00788](http://ligeirinha.lncc.br/cd-bin/annotation/annotation.cgi?id=0&gene=CD00788) [AGDE01071](http://ligeirinha.lncc.br/cd-bin/annotation/annotation.cgi?id=0&gene=CD01071) [AGDE01420](http://ligeirinha.lncc.br/cd-bin/annotation/annotation.cgi?id=0&gene=CD01420)I [AGDE03823](http://ligeirinha.lncc.br/cd-bin/annotation/annotation.cgi?id=0&gene=CD03823) [AGDE06263](http://ligeirinha.lncc.br/cd-bin/annotation/annotation.cgi?id=0&gene=CD06263) [AGDE07467](http://ligeirinha.lncc.br/cd-bin/annotation/annotation.cgi?id=0&gene=CD07467) [AGDE09651](http://ligeirinha.lncc.br/cd-bin/annotation/annotation.cgi?id=0&gene=CD09651) [AGDE10078](http://ligeirinha.lncc.br/cd-bin/annotation/annotation.cgi?id=0&gene=CD10078) [AGDE11858](http://ligeirinha.lncc.br/cd-bin/annotation/annotation.cgi?id=0&gene=CD11858) | [STCU01125](http://ligeirinha.lncc.br/bc-bin/annotation/annotation.cgi?id=0&gene=BC01125) [STCU03590](http://ligeirinha.lncc.br/bc-bin/annotation/annotation.cgi?id=0&gene=BC03590) [STCU06009](http://ligeirinha.lncc.br/bc-bin/annotation/annotation.cgi?id=0&gene=BC06009) [STCU06071](http://ligeirinha.lncc.br/bc-bin/annotation/annotation.cgi?id=0&gene=BC06071) [STCU06494](http://ligeirinha.lncc.br/bc-bin/annotation/annotation.cgi?id=0&gene=BC06494) [STCU08551](http://ligeirinha.lncc.br/bc-bin/annotation/annotation.cgi?id=0&gene=BC08551) [STCU09493](http://ligeirinha.lncc.br/bc-bin/annotation/annotation.cgi?id=0&gene=BC09493) |  |  | EC 1.1.1.205 | inosine monophosphate dehydrogenase |
| [AGDE00910](http://ligeirinha.lncc.br/cd-bin/annotation/annotation.cgi?id=0&gene=CD00910) [AGDE01485](http://ligeirinha.lncc.br/cd-bin/annotation/annotation.cgi?id=0&gene=CD01485) [AGDE03390](http://ligeirinha.lncc.br/cd-bin/annotation/annotation.cgi?id=0&gene=CD03390) [AGDE03427](http://ligeirinha.lncc.br/cd-bin/annotation/annotation.cgi?id=0&gene=CD03427) [AGDE06014](http://ligeirinha.lncc.br/cd-bin/annotation/annotation.cgi?id=0&gene=CD06014) [AGDE06581](http://ligeirinha.lncc.br/cd-bin/annotation/annotation.cgi?id=0&gene=CD06581) | [STCU02750](http://ligeirinha.lncc.br/bc-bin/annotation/annotation.cgi?id=0&gene=BC02750) [STCU07068](http://ligeirinha.lncc.br/bc-bin/annotation/annotation.cgi?id=0&gene=BC07068) [STCU08293](http://ligeirinha.lncc.br/bc-bin/annotation/annotation.cgi?id=0&gene=BC08293) STCU[09036](http://ligeirinha.lncc.br/bc-bin/annotation/annotation.cgi?id=0&gene=BC09036) [STCU09716](http://ligeirinha.lncc.br/bc-bin/annotation/annotation.cgi?id=0&gene=BC09716) [STCU09856](http://ligeirinha.lncc.br/bc-bin/annotation/annotation.cgi?id=0&gene=BC09856) [STCU09898](http://ligeirinha.lncc.br/bc-bin/annotation/annotation.cgi?id=0&gene=BC09898) |  |  | EC 6.3.5.2 | GMP synthetase |
|  |  | CKCE00290 | CKBE00436 | 2.4.2.14 | PRPP amidotransferase |
|  |  | CKCE00399 | CKBE00331 | 6.3.4.13 | GAR synthetase |
|  |  | CKCE00369 | CKBE00360 | 2.1.2.2 | GAR transformylase |
|  |  | CKCE00295 | CKBE00432 | 6.3.5.3 | formylgycinamidineribonucleotide (FGAM) synthetase |
|  |  | CKCE00045 | CKBE00672 | 6.3.3.1 | AIR-synthetase |
|  |  | CKCE00707 | CKBE00304 | 6.3.5.5 | glutamine-dependent carbamoylphosphatesynthetase |
|  |  | CKCE00397 | CKBE00333 | 2.1.3.2 | aspartate transcarbamoylase |
|  |  | CKCE00025 | CKBE00692 | 3.5.2.3 | dihydroorotase |
|  |  | CKCE00069 | CKBE00645 | 1.3.98.1 | dihydroorotate dehydrogenase |
|  |  | CKCE00494 | CKBE00087 | 2.4.2.10 | orotatephosphoribosyltransferase |
|  |  | CKCE00325 | CKBE00401 | 4.1.23 | orotidylate decarboxylase |
|  |  | CKCE00155 | CKBE00560 | 6.3.4.2 | CTP synthase |
| [AGDE02276](http://ligeirinha.lncc.br/cd-bin/annotation/annotation.cgi?id=0&gene=CD02276) [AGDE07383](http://ligeirinha.lncc.br/cd-bin/annotation/annotation.cgi?id=0&gene=CD07383) | [STCU01796](http://ligeirinha.lncc.br/bc-bin/annotation/annotation.cgi?id=0&gene=BC01796) [STCU02368](http://ligeirinha.lncc.br/bc-bin/annotation/annotation.cgi?id=0&gene=BC02368) [STCU03361](http://ligeirinha.lncc.br/bc-bin/annotation/annotation.cgi?id=0&gene=BC03361) [STCU06002](http://ligeirinha.lncc.br/bc-bin/annotation/annotation.cgi?id=0&gene=BC06002) |  |  | 2.4.2.9 | Phosphoribosyltransferase |
|  |  | CKCE00083 | CKBE00633 | 1.8.1.9 | Thioredoxireductase |
| [AGDE01524](http://ligeirinha.lncc.br/cd-bin/annotation/annotation.cgi?id=0&gene=CD01524) [AGDE01961](http://ligeirinha.lncc.br/cd-bin/annotation/annotation.cgi?id=0&gene=CD01961) [AGDE02725](http://ligeirinha.lncc.br/cd-bin/annotation/annotation.cgi?id=0&gene=CD02725) [AGDE02862](http://ligeirinha.lncc.br/cd-bin/annotation/annotation.cgi?id=0&gene=CD02862) [AGDE02959](http://ligeirinha.lncc.br/cd-bin/annotation/annotation.cgi?id=0&gene=CD02959) [AGDE06771](http://ligeirinha.lncc.br/cd-bin/annotation/annotation.cgi?id=0&gene=CD06771) [AGDE07134](http://ligeirinha.lncc.br/cd-bin/annotation/annotation.cgi?id=0&gene=CD07134) [AGDE07327](http://ligeirinha.lncc.br/cd-bin/annotation/annotation.cgi?id=0&gene=CD07327) [AGDE07858](http://ligeirinha.lncc.br/cd-bin/annotation/annotation.cgi?id=0&gene=CD07858) [AGDE07925](http://ligeirinha.lncc.br/cd-bin/annotation/annotation.cgi?id=0&gene=CD07925) [AGDE09473](http://ligeirinha.lncc.br/cd-bin/annotation/annotation.cgi?id=0&gene=CD09473) [AGDE10415](http://ligeirinha.lncc.br/cd-bin/annotation/annotation.cgi?id=0&gene=CD10415) | [STCU02156](http://ligeirinha.lncc.br/bc-bin/annotation/annotation.cgi?id=0&gene=BC02156) [STCU02261](http://ligeirinha.lncc.br/bc-bin/annotation/annotation.cgi?id=0&gene=BC02261) [STCU02337](http://ligeirinha.lncc.br/bc-bin/annotation/annotation.cgi?id=0&gene=BC02337) [STCU05286](http://ligeirinha.lncc.br/bc-bin/annotation/annotation.cgi?id=0&gene=BC05286) [STCU05536](http://ligeirinha.lncc.br/bc-bin/annotation/annotation.cgi?id=0&gene=BC05536) [STCU05725](http://ligeirinha.lncc.br/bc-bin/annotation/annotation.cgi?id=0&gene=BC05725) [STCU05779](http://ligeirinha.lncc.br/bc-bin/annotation/annotation.cgi?id=0&gene=BC05779) [STCU06878](http://ligeirinha.lncc.br/bc-bin/annotation/annotation.cgi?id=0&gene=BC06878) [STCU06883](http://ligeirinha.lncc.br/bc-bin/annotation/annotation.cgi?id=0&gene=BC06883) [STCU07142](http://ligeirinha.lncc.br/bc-bin/annotation/annotation.cgi?id=0&gene=BC07142) [STCU07588](http://ligeirinha.lncc.br/bc-bin/annotation/annotation.cgi?id=0&gene=BC07588) [STCU07875](http://ligeirinha.lncc.br/bc-bin/annotation/annotation.cgi?id=0&gene=BC07875) [STCU08084](http://ligeirinha.lncc.br/bc-bin/annotation/annotation.cgi?id=0&gene=BC08084) [STCU08268](http://ligeirinha.lncc.br/bc-bin/annotation/annotation.cgi?id=0&gene=BC08268) [STCU09151](http://ligeirinha.lncc.br/bc-bin/annotation/annotation.cgi?id=0&gene=BC09151) | CKCE00439 | CKBE00031 | 1.17.4.1 | ribonucleotidereductase |
| [AGDE02269](http://ligeirinha.lncc.br/cd-bin/annotation/annotation.cgi?id=0&gene=CD02269) [AGDE02882](http://ligeirinha.lncc.br/cd-bin/annotation/annotation.cgi?id=0&gene=CD02882) [AGDE03361](http://ligeirinha.lncc.br/cd-bin/annotation/annotation.cgi?id=0&gene=CD03361)[AGDE03889](http://ligeirinha.lncc.br/cd-bin/annotation/annotation.cgi?id=0&gene=CD03889) [AGDE05911](http://ligeirinha.lncc.br/cd-bin/annotation/annotation.cgi?id=0&gene=CD05911) | [STCU00393](http://ligeirinha.lncc.br/bc-bin/annotation/annotation.cgi?id=0&gene=BC00393) [STCU00716](http://ligeirinha.lncc.br/bc-bin/annotation/annotation.cgi?id=0&gene=BC00716) [STCU02683](http://ligeirinha.lncc.br/bc-bin/annotation/annotation.cgi?id=0&gene=BC02683) [STCU06110](http://ligeirinha.lncc.br/bc-bin/annotation/annotation.cgi?id=0&gene=BC06110) | CKCE00661 | CKBE00255 | 2.1.1.45 | thymidilate kinase |
