# Supplementary material for: Predicting the Proteins of Angomonas deanei, Strigomonas culicis and Their Respective Endosymbionts Reveals New Aspects of the Trypanosomatidae Family
Source: PLoS One. 2013 Apr 3;8(4):e60209. doi: 10.1371/journal.pone.0060209 (PMC3616161; doi:10.1371/journal.pone.0060209)
Supplement: Table S20 — Glysosyltransferases found in A. deanei and S. culicis. (DOC) [file pone.0060209.s027.doc]

**Table 20**. Glysosyltransferases found in *A. deanei* and *S. culicis.*

| **Classification** | **Product** | **ID** | |
| --- | --- | --- | --- |
|  |  | ***S. culicis*** | ***A. deanei*** |
| GT-A family | mannose-1-phosphate guanylyltransferase | STCU09488 STCU07665 |  |
|  | galactofuranosyltransferase | STCU09574 STCU09836 STCU05969 STCU10651 STCU07793 STCU04365 STCU10401 STCU06524 STCU01250 |  |
|  | UDP-glucose:glycoprotein glucosyltransferase | STCU08753 |  |
|  | dolichol-phosphate mannosyltransferase | STCU03097 | AGDE09920 AGDE03781 AGDE00191 |
| GT-B family | beta-1,4-N-acetylglucosaminyltransferase | STCU05058 | AGDE02303 AGDE11937 |
|  | beta-1,4-mannosyltransferase | STCU08259 STCU09566 STCU09312 STCU07312 |  |
|  | alpha-1,2 mannosyltransferase | STCU01246 STCU02612 STCU06719 STCU08255 STCU09226 |  |
|  | alpha-1,3/alpha-1,6-mannosyltransferase | STCU04757 STCU07898 | AGDE03004 AGDE00414 |
|  | sucrose-phosphate synthase-like protein | STCU00661 STCU09767 STCU05179 STCU09139 | AGDE05845 AGDE05050 AGDE08553 AGDE01786 |
|  | UDP-glucoronosyl and UDP-glucosyl transferase |  | AGDE09000 |
| glycosyltransferase family 25 | glycosyltransferase family-like protein | STCU00348 STCU05905 STCU08720 | AGDE11122 |
| Others | UDP-GlcNAc:polypeptide alpha-N-acetylglucosaminyltransferase | STCU06221 STCU10173 | AGDE08689 AGDE11058 |
|  | UDP-Gal or UDP-GlcNAc-dependent glycosyltransferase |  | AGDE16963 |
|  | UDP-N-acetylglucosamine-dolichyl-phosphate N-acetylglucosaminephosphotransferase |  | AGDE11861 AGDE10096 AGDE01438 |
|  | dolichyl-diphosphooligosaccharide-protein glycosyltransferase | STCU09395 | AGDE05788 |
|  | alpha-1,3 mannosyltransferase | STCU05108 STCU05637 STCU07624 | AGDE06391 AGDE07689 |
|  | UDP-GlcNAc:PI A1-6 GlcNAc-transferase |  | AGDE05272 AGDE00487 |
|  | phosphatidylinositol glycan - class A | STCU05963 | AGDE10398 AGDE02195 |
|  | phosphatidylinositol glycan, class M | STCU07300 STCU09061 STCU03394 | AGDE01044 AGDE04190 AGDE04253 AGDE10705 |
|  | phosphatidylinositol glycan - class B | STCU07568 STCU02554 STCU07380 | AGDE15893 |
|  |  |  |  |
