# Supplementary material for: Predicting the Proteins of Angomonas deanei, Strigomonas culicis and Their Respective Endosymbionts Reveals New Aspects of the Trypanosomatidae Family
Source: PLoS One. 2013 Apr 3;8(4):e60209. doi: 10.1371/journal.pone.0060209 (PMC3616161; doi:10.1371/journal.pone.0060209)
Supplement: Table S21 — Surface proteins of A. deanei e S. culicis. (DOC) [file pone.0060209.s028.doc]

**Table S21.** Surface proteins of *A. deanei* and *S. culicis.*

| **Amastin** | | **GP63** | | **Cysteine peptidases** | | | | |
| --- | --- | --- | --- | --- | --- | --- | --- | --- |
| ***A. deanei*** | ***S. culicis*** | ***A. deanei*** | ***S. culicis*** | ***A. deanei*** | | | ***S. culicis*** | |
| AGDE00319 | STCU00596 | AGDE11365 | STCU00027 | | AGDE00043 | | STCU00038 | |
| AGDE01982 | STCU01302 | AGDE06764 | STCU00614 | | AGDE00044 | | STCU00359 | |
| AGDE2634 | STCU01560 | AGDE00152 | STCU02880 | | AGDE00119 | | STCU00385 | |
| AGDE03200 | STCU02220 | AGDE12426 | STCU07592 | | AGDE00130 | | STCU00385 | |
| AGDE05080 | STCU02798 | AGDE10677 | STCU07902 | | AGDE00469 | | STCU00407 | |
| AGDE05783 | STCU03154 | AGDE09650 | STCU08786 | | AGDE00500 | | STCU00533 | |
| AGDE05979 | STCU03229 | AGDE06859 | STCU09360 | | AGDE00645 | | STCU00853 | |
| AGDE06849 | STCU03602 | AGDE04528 | STCU09413 | | AGDE00853 | | STCU00853 | |
| AGDE07707 | STCU03623 | AGDE15581 | STCU10437 | | AGDE00928 | | STCU01414 | |
| AGDE08158 | STCU05149 | AGDE14174 |  | AGDE01905 | | | STCU01414 | |
| AGDE08978 | STCU05495 | AGDE12294 |  | AGDE02024 | | | STCU01651 | |
| AGDE10087 | STCU06626 | AGDE09038 |  | AGDE02055 | | | STCU01676 | |
| AGDE10304 | STCU06954 | AGDE08018 |  | AGDE02450 | | | STCU01935 | |
| AGDE10321 | STCU11252 | AGDE07823 |  | AGDE02784 | | | STCU01935 | |
| AGDE11600 |  | AGDE02931 |  | AGDE02856 | | | STCU02207 | |
| AGDE11994 |  | AGDE15507 |  | AGDE03013 | | | STCU02207 | |
| AGDE12317 |  | AGDE01404 |  | AGDE03099 | | | STCU02258 | |
| AGDE12812 |  | AGDE12059 |  | AGDE03307 | | | STCU02258 | |
| AGDE13103 |  | AGDE08697 |  | AGDE03391 | | | STCU02891 | |
| AGDE13336 |  | AGDE06992 |  | AGDE03560 | | | STCU03217 | |
| AGDE14031 |  | AGDE14753 |  | AGDE03582 | | | STCU03217 | |
| AGDE14637 |  | AGDE11898 |  | AGDE03833 | | | STCU03543 | |
| AGDE15097 |  | AGDE09429 |  | AGDE04132 | | | STCU03543 | |
| AGDE15140 |  | AGDE07566 |  | AGDE04151 | | | STCU03561 | |
| AGDE15153 |  | AGDE04363 |  | AGDE04520 | | | STCU03633 | |
| AGDE15186 |  | AGDE02834 |  | AGDE04600 | | | STCU03688 | |
| AGDE15443 |  | AGDE16584 |  | AGDE05069 | | | STCU03688 | |
| AGDE15899 |  | AGDE15543 |  | AGDE06003 | | | STCU03850 | |
| AGDE16942 |  | AGDE16538 |  | AGDE06234 | | | STCU04067 | |
| AGDE17184 |  | AGDE16537 |  | AGDE06479 | | | STCU04200 | |
|  |  | AGDE12855 |  | AGDE06753 | | | STCU04638 | |
|  |  | AGDE16238 |  | AGDE07283 | | | STCU04638 | |
|  |  | AGDE13503 |  | AGDE07311 | | | STCU04669 | |
|  |  | AGDE04020 |  | AGDE07560 | | | STCU04669 | |
|  |  | AGDE11968 |  | AGDE07879 | | | STCU04678 | |
|  |  | AGDE10297 |  | AGDE07884 | | | STCU04678 | |
|  |  | AGDE10404 |  | AGDE08152 | | | STCU04722 | |
|  |  |  |  | AGDE08196 | | | STCU04770 | |
|  |  |  |  | AGDE08358 | | | STCU04903 | |
|  |  |  |  | AGDE08384 | | | STCU04910 | |
|  |  |  |  | AGDE08559 | | | STCU04937 | |
|  |  |  |  | AGDE08846 | | | STCU04937 | |
|  |  |  |  | AGDE09079 | | | STCU05644 | |
|  |  |  |  | AGDE09241 | | | STCU05644 | |
|  |  |  |  | AGDE09273 | | | STCU06053 | |
|  |  |  |  | AGDE09409 | | | STCU06053 | |
|  |  |  |  | AGDE09770 | | | STCU06065 | |
|  |  |  |  | AGDE10008 | | | STCU06065 | |
|  |  |  |  | AGDE10359 | | | STCU06749 | |
|  |  |  |  | AGDE10514 | | | STCU06749 | |
|  |  |  |  | AGDE10521 | | | STCU07146 | |
|  |  |  |  | AGDE10681 | | STCU07219 | |  |
|  |  |  |  | AGDE10767 | | STCU07355 | |  |
|  |  |  |  | AGDE11054 | | STCU07655 | |  |
|  |  |  |  | AGDE11187 | | STCU07865 | |  |
|  |  |  |  | AGDE11307 | | STCU08161 | |  |
|  |  |  |  | AGDE11358 | | STCU08270 | |  |
|  |  |  |  | AGDE11372 | | STCU08270 | |  |
|  |  |  |  | AGDE11622 | | STCU08300 | |  |
|  |  |  |  | AGDE11654 | | STCU08303 | |  |
|  |  |  |  | AGDE11712 | | STCU08303 | |  |
|  |  |  |  | AGDE12231 | | STCU08771 | |  |
|  |  |  |  | AGDE12917 | | STCU08771 | |  |
|  |  |  |  | AGDE13128 | | STCU08777 | |  |
|  |  |  |  | AGDE13503 | | STCU09194 | |  |
|  |  |  |  | AGDE13506 | | STCU09282 | |  |
|  |  |  |  | AGDE14238 | | STCU09282 | |  |
|  |  |  |  | AGDE14294 | | STCU09462 | |  |
|  |  |  |  | AGDE14323 | | STCU09557 | |  |
|  |  |  |  | AGDE14383 | | STCU09557 | |  |
|  |  |  |  | AGDE14696 | | STCU09587 | |  |
|  |  |  |  | AGDE14698 | | | STCU09596 | |
|  |  |  |  | AGDE14781 | | | STCU09714 | |
|  |  |  |  | AGDE14782 | | | STCU09775 | |
|  |  |  |  | AGDE14783 | | | STCU09775 | |
|  |  |  |  | AGDE15042 | | | STCU09807 | |
|  |  |  |  | AGDE15106 | | | STCU09807 | |
|  |  |  |  | AGDE15107 | | | STCU09819 | |
|  |  |  |  | AGDE15382 | | | STCU09824 | |
|  |  |  |  | AGDE15788 | | | STCU09838 | |
|  |  |  |  | AGDE16059 | | | STCU10352 | |
|  |  |  |  | AGDE16060 | | | STCU10743 | |
|  |  |  |  | AGDE16203 | | | STCU10784 | |
|  |  |  |  |  | | | STCU10784 | |
|  |  |  |  |  | | | STCU11546 | |
