# Supplementary material for: Predicting the Proteins of Angomonas deanei, Strigomonas culicis and Their Respective Endosymbionts Reveals New Aspects of the Trypanosomatidae Family
Source: PLoS One. 2013 Apr 3;8(4):e60209. doi: 10.1371/journal.pone.0060209 (PMC3616161; doi:10.1371/journal.pone.0060209)
Supplement: Text S1 — (DOC) [file pone.0060209.s029.doc]

# Detailed analysis of housekeeping genes of *A. deanei* and *S. culicis*

# *Histones and histone-modifying enzymes*

The N-terminal sequences of histones are sites of post-translational modifications and are quite divergent in trypanosomatids compared to other eukaryotes. For the histone H4, the lysine K4 and K10 acetylation sites are present in *A. deanei* and *S. culicis* (Figure S3). In contrast, the K14 site is absent in both, as in most *Leishmania* species . In histone H3, *A. deanei* and *S. culicis* have conserved K4, K10, K36, and K76 methylation sites. The putative acetylation sites on K19 and K16 of histone H3 are also conserved. Interestingly, both symbiont-bearing protozoa have a lysine in position 47, like most *Leishmania* species, but this is absent in *Trypanosoma*. In addition, only *S. culicis* has a lysine at position 46, and this lysine could be uniquely modified. Concerning histone H2A and the variant H2AZ, *A. deanei*, *S. culicis*, and other trypanosomes have lysines at the C-terminus that were shown to undergo extensive acetylation in *T. brucei*. Some histone H2A genes of *A. deanei* and *S. culicis* also contain putative phosphorylation sites of the newly identified γH2A in response to DNA damage . The two species also have histone H2B and H2B variants, but both display the conserved acetylation site in the K4 residue that is present in *T. brucei* but is not found in *Leishmania* species. Moreover, another acetylated site, lysine K12, is conserved in *Trypanosoma,* and while it is present in *A. deanei*, it is absent in *S. culicis*. Intriguingly, four genes of *S. culicis* entirely lack the N-terminal sequence of H2B.

Several acetyltransferases similar to histone acetyltransferases are detected in *A.* *deanei* and S*. culicis.* Both species have genes encoding HAT1 orthologs, but only *A. deanei* has genes for HAT2 and HAT3 belonging to the MYST family (Table S2). However, these species lack genes for HAT4, also absent in *T. brucei* but not in *T. cruzi* or *Leishmania*. Other putative acetyltransferases are present in both families, particularly genes of the eIP3B group. Another set of HAT genes, the ELP family with a unique radical SAM containing a 4Fe-4S center, is present in both species and in all trypanosomes.

In trypanosomatids, three types of Silent regulator information 2 proteins (Sir2p) have been described . These are conserved NAD-dependent deacetylases , originally described to deacetylate histones, and have been shown to promote silencing of gene expression in yeast. Trypanosome Sir2p-1 is localized in the nucleus and Sir2p-2 and 3 in the mitochondria. These three types of Sir2p are detected in *A. deanei* and *S. culicis* (Table S3). Similar sequences were also found in both symbiont species, but these genes are more closely related to those identified in *B. petri* (Figure S4), indicating that no transfers between the host and symbiont have occurred for these genes. Five and seven ORFs for other putative histone deacetylases are present in *A. deanei* and *S. culicis*, respectively (Table S4). Of these sequences, one in *A. deanei* and three in *S. culicis* are similar to HDAC1, which is essential in *T. brucei* . These two species also have sequences of HDAC2, a non-essential enzyme in *T. brucei*, which is absent in *Leishmania* species. Another histone deacetylase present in both symbiont-bearing species that is not essential in *T. brucei* is HDAC4. However, no orthologs of HDAC3 were found in *A. deanei*, or *S. culicis*, although some ORFs display similarity to the HDAC3 genes of other trypanosomes. Importantly, each endosymbiont has one deacetylase that does not match the sequences predicted in the host genome based on typical prokaryotic histone deacetylases.

The *A. deanei* and *S. culicis* sequences listed in Table S5 mainly match the histone methyltransferases DOT1a and DOT1b, also described in other trypanosomatids. These enzymes methylate Lys-76 of histone H3 , and their presence is consistent with the conservation of Lys 76 of histone H3 in *A. deanei* and *B. culicis*. K76 methylation is related to cell cycle control in trypanosomes. In other eukaryotes, this is achieved by interactions with proteins such as Rad9 in yeast and 53BP1 in mammals . However, no orthologs of these proteins are detected in Kinetoplastidae, including *A. deanei* and *S. culicis*. Additionally, three SET (**S**u(var)3-9, **E**nhancer of Zeste, **T**rithorax) proteins have been identified in each symbiont bearing species, comprising two different enzymes. These enzymes are known to methylate histone H3 at lysines K4 and K36 present in *A. deanei* and *S. culicis*. One of the transferases contains a glutathione synthase ATP-binding domain (AGDE 07479/AGDE08618 and STCU 04838) at the C-terminus, and the others have no other conserved domain (AGDE07752 and STCU04925/STCU05938).

These observations suggest that the chromatin of *A. deanei* and *S. culicis* will exhibit the same patterns of modification as other trypanosomatids with small differences, most likely associated with the origins of these species rather than the presence of endosymbionts.

Assembly and remodeling of chromatin during new DNA synthesis, repair and transcription depends on the presence of histone chaperones . *A. deanei* and *S. culicis* display two forms of Asf1 (anti-silencing function), the histone chaperone for histones H4 and H3, similarly to other Trypanosomatids (Table S6). These two species also contain two similar copies of NAP-1, a protein involved in the transport of H2A and H2B to the nucleus. The other protein involved in chromatin assembly in eukaryotes is called chromatin assembly factor 1 (CAF1). This factor is formed by three subunits. Two are detected in all trypanosomatids (the larger ones, A and B). Subunit C is found in *A. deanei*, but not in *S. culicis*. Only in *A. deanei*, one subunit of the “facilitates chromatin transcription” (FACT) complex was detected, while three subunits are found in other trypanosomatids. The reasons for the specific absence of these proteins in the endosymbiont-bearing species are unclear. Their absence may be related to the fact that these species replicate much faster than other trypanosomes.

There are four recognized bromodomain proteins in trypanosomatids (Brd1, 2, 3 and 4) that bind acetylated lysines in several proteins including histones . These four classes are present in *A. deanei*, but only Brd2 was identified in *S. culicis* (Table S7). The lack of Brd1, 3 and 4 in *S. culicis* is unexpected, and could suggest a simplified regulatory role for histone modifications in this species. As expected, no chromo domain proteins are detected in *A. deanei* and *S. culicis*. This type of protein binds to mono, di and trimethylated lysines, such as those found in histones, and are also not found in other trypanosomatids.

## Kinetoplast DNA replication

*A. deanei* and *S. culicis* have genes encoding the major proteins involved in the replication of kDNA (Table S8), already described and characterized in *C. fasciculata* and *T. brucei* . Investigation of both the *A*. *deanei* and *S. culicis* genomes also revealed the presence of DNA Topoisomerases IA, IB (large and small subunits), II mitochondrial, II alpha, III alpha and III beta, which are also present in other Kinetoplastida . *A. deanei* and *S. culicis* display one helicase each, and curiously, helicase I and II are found in the endosymbiont genomes. This contrasts with *T. brucei,* which encodes eight PIF1-like helicases, six of which are mitochondrial, as well as TbPIF-2, that is essential for maxicircle replication . Thus, it remains unknown whether helicases of bacterial origin can act in the trypanosomatids. Polymerase-beta (Pol-β) is present in *A. deanei* and *S. culicis.* In *T. cruzi*, this protein is involved in kDNA replication and the repair of oxidative lesions . *A. deanei* and *S. culicis* also contain DNA polymerase III-ε, but lack Pol III-α and Pol III-γ. The *A. deanei* endosymbiont has all these DNA polymerases III, while in *S. culicis* the symbiont has only Pol III-α and Pol III-ε.

## DNA replication

Differently from most eukaryotes but similar to other trypanosomatids, *A. deanei* and *S. culicis* do not have sequences that code for protein subunits of the pre-replication complex (pre-RC) that compose the eukaryotic Origin Recognition Complex (ORC) . Instead, these protozoa have a gene with a sequence highly related to Cdc6, which is similar to Orc1 (Table S9). In trypanosomatids, including *A. deanei* and *S. culicis*, this protein is named Orc1/Cdc6, which is able to replace Cdc6 in a yeast complementation assay . In *T. brucei*, two other Orc1/Cdc6 interacting proteins were recently identified and sequences with similarities can be found in our database, but further studies are necessary to confirm whether they are Orc-like factors*. A. deanei* and *S. culicis* contain subunits of the Minichromosome Maintenance (MCM) helicase complex, which is essential for DNA replication , and are homologous to those in *S. cerevisiae*, suggesting that these organisms use a heterohexamer helicase, as expected for eukaryotic organisms . However, as in other trypanosomes, both symbiont-bearing species lack genes homologous to Cdt1, the eukaryotic helicase loader. We speculate that Orc1/Cdc6 recruits the MCM complex, as observed in Archaea . Another possibility is that an unknown protein could recruit MCM to the replication origins in a manner analogous to Cdt1. *A. deanei* also has Cdc45 and GINS, which together convert inactive MCM to active Cdc45-Mcm-GINS (CMG) replicative helicase .

The three DNA polymerases α, ε and δ, which are essential for DNA replication, are present in the genomes of *A. deanei* and *S. culicis* (Table S9)*.* DNA polymerase α-primase complex subunits α1, α2, Pri1 and Pri2 are identified in *A. deanei.* However, only the subunits α1 and Pri2 were found in *S. culicis*. A prokaryotic DNA polymerase I was identified in both trypanosomatid species, as well as the subunits RNase H, DpoI and SSB (single-strand binding). These could be involved in the mitochondrion replication. Four ORFs coding for Proliferating Cell Nuclear Antigen (PCNA), which is also essential for DNA replication and for clamping the replication machinery to the DNA , are present in the genome of *A. deanei,* and three are present in the *S. culicis* genome. In *S. culicis* these ORFs represent multiple copies of the same protein, which means that in this organism, as well as in all eukaryotes, PCNA might be a multimeric complex composed of the same subunit. The four PCNA ORFs in *A. deanei* are very similar, but one copy (AGDE08212) lacks the first 49 amino acids, while another copy (AGDE02121) lacks the last 25 amino acids. As eukaryotic PCNA is composed of identical subunits , it is necessary to verify whether these shorter subunits are part of the PCNA clamp molecule.

Eukaryotic Replication Factor C (RFC) clamp loader consists of five subunits, one large (RFCL) and four small subunits (RFCS). RFCS is composed of 1, 2, or 4 distinct sequence types depending on the phylogenetic group. *A. deanei* contains all five subunits, as described for eukaryotes . Finally, replication Protein A (RPA), a single-stranded DNA-binding protein , is also present in both genomes. Taken together, our genomic searches indicate that *A. deanei* and *S. culicis* have DNA replication mechanisms similar to that described for other eukaryotes.

*A. deanei* and *S. culicis* symbiotic bacteria contain the typical prokaryotic machinery of DNA replication, including the initiator DnaA, the helicase component DnaB, the primase DnaG and DNA polymerase III (Table S10). DNA replication in the *A. deanei* endosymbiont involves the DNA polymerase III complex subunits α, β, ε, γ and τ, while for the endosymbiont of *S. culicis* only the subunits α, β, and ε were identified. No DnaC sequence was found in the endosymbiont genomes. DnaC is usually essential for DNA replication, but coliphages l and P2, which do not need the host cell DnaC, encode their own DnaC analogues to load DnaB at their respective origins . Therefore, further analysis could confirm whether DnaC or its orthologs are present in endosymbiont genomes.

## DNA repair and microsatellite variations among the species

As for other trypanosomes, both symbiont-bearing protozoa contain DNA repair systems such as nucleotide and base excision repair (NER and BER), post-replicative and mismatch repair (MMR), and homologous recombination . Conserved forms of RAD23, RAD50, and RAD51 are present in *A. deanei* and *S. culicis*. Similarly to other trypanosomatids, the pathway of non-homologous end joining is incomplete in *A. deanei* and *S. culicis*, although the homologs KU80 and putative DNA ligase are present. KU80 and these other proteins may be involved in telomere length regulation, as shown for *T. brucei* .

Components of the bacterial nucleotide excision repair mechanism are also present in the endosymbionts of *A. deanei* and *S. culicis*. Damage recognition, DNA incision, and nucleotide excision are mediated by the ABC exonuclease by the action of the gene products UvrA, UvrB, and UvrC, which assemble on DNA at the site of the altered nucleotides . These gene products are found in the endosymbionts. In addition, the two species contain the subunit UvrD, which is related to the resistance to thymine starvation and death as described for *E. coli* .

## RNA Metabolism

At least 96 and 61 ORFs related to transcription, splicing, and RNA transport are present in the *A. deanei* and *S. culicis* databases, respectively (Table 3 and Table S11). The RNAP I, II, and III components resemble the genes in other trypanosomatids, which differ considerably from those of most eukaryotic organisms . The endosymbionts of *A. deanei* and *S. culicis* contain the RNA polymerase (RNAP) α, β, and ω subunits (RpoD and RpoH) (Table S12). These are conserved when compared to other bacteria, as is the transcription-repair coupling factor (TRCF).

Spliced-leader RNA (SL-RNA) is encoded by multiple copies of a gene in trypanosomatids. A 39-nucleotide sequence of SL-RNA is inserted in the 5' end of all nuclear mRNAs in a trans-splicing reaction. Comparative sequence analysis of these 39 nucleotides found in the SL gene exon of *S. culicis* and *A. deanei* was carried with previously known SL genes. Both neighbor-joining and maximum parsimony analysis (1000 bootstraps) revealed the same tree topology, clustering together the digenetic and mammalian infective *T. cruzi* and *T. rangeli* in a single branch, despite the low bootstrap value (Figure S5). Sequences from both *A. deanei* and *S. culicis* SL genes are clustered with their homologous genes retrieved from GenBank in clearly separated branches. Except for *A. deanei* sequences, and despite the reduced bootstrap values, *S. culicis* sequences were clustered with those of the other monoxenic species (*Leptomonas* and *Herpetomonas*), while both *Crithidia* sequences were clustered in a single branch with a high bootstrap value. Ribonucleoprotein components for mRNA processing such as U1, U2, U2-related, U4/6 and U5 proteins associated with *A. deanei* and *S. culicis* are listed in Table S11. These components are similar to those of other trypanosomatids, as are the proteins involved in the mRNA surveillance pathway and RNA transport in both organisms.

Table S11 also lists the genes encoding for potential RNAP II basal transcription factor homologues in *A. deanei* and *S. culicis.* Components of eukaryotic basal transcription factors are well represented in both symbiont-containing trypanosomatids. The prokaryotic transcription machinery is maintained in both endosymbiont genomes.

## Translation

The components of the translation machinery of both *A. deanei* and *S. culicis* are similar to the genes described for other trypanosomes, including conserved components related to ribosome biogenesis, the mRNA surveillance pathway, and RNA transport in eukaryotes. Proteins for aminoacyl t-RNA biosynthesis are found in *A. deanei* and *S. culicis* together with key molecules related to translation listed in Table S13. Initiation and elongation factors for protein synthesis are present in the host protozoa symbiont.

Endosymbionts also contain most enzymes needed for prokaryotic protein synthesis, such as methionyl-tRNA formyltransferase and initiation and elongation factors. The presence of elongation factors (Ts and Tu) in symbionts of both trypanosomatids reinforces the idea that such bacteria are capable of autonomous protein synthesis .

# Materials and Methods

## Cell culture

*Angomonas deanei* was isolated from *Zelus leucogrammus* in 1973 by A.L.M. Carvalho (ATCC 30255). *Strigomonas culicis* was isolated from *Aedes vexans* in 1942 by F.G. Wallace (ATCC 30268). Both symbiont-containing strains were grown at 28°C for 24 h in Warren culture medium supplemented with 10% fetal calf serum.

## DNA extraction

Genomic DNA was extracted from cultured trypanosomatids using the phenol/chloroform method. To minimize the amount of kinetoplast DNA present in the sequencing reaction, the total genomic DNA (~50 µg in 300 µL) from each trypanosomatid isolate was loaded onto a 0.8% low melting agarose gel in 0.5 x TAE buffer. Electrophoresis was performed at a low voltage not exceeding 4 V/cm for 4–5 hours. After electrophoresis, the gel was washed in distilled water for ~1 hour for destaining and buffer removal. The genomic band was carefully cut out and purified by treatment with beta-agarase I (New England Biolabs) as described by the manufacturer. The DNA was precipitated with ethanol in 0.3 M sodium acetate, washed with 70% ethanol, dried, and finally dissolved in 100–120 µL TE, pH 8.0. Our results (not shown) indicate that this genomic DNA enrichment protocol reduces the proportion of kDNA in the sample to less than 5%, and therefore provides a template that yields coverage of both genomic and kDNA.

## DNA library construction and sequencing

Library generation and sequencing were performed at Computational Genomics Unity Darcy Fontoura de Almeida (UGCDFA) of the National Laboratory of Scientific Computation (LNCC) (Petrópolis, RJ, Brazil). For the 454 GS-FLX Titanium sequencing, each library was constructed using 5 µg of genomic DNA following the GS FLX Titanium series protocols. Three sequencing libraries were prepared from *A. deanei* gDNA: two Shotgun Libraries (SG) and one 3 kb Paired End Library (PE); and two sequencing libraries were prepared from *S. culicis* gDNA: one SG library and one PE library. All titrations, emulsions, PCR, and sequencing steps were carried out according to the manufacturer's protocol. One of the SG libraries of *A. deanei* was sequenced using one of the two regions of a PicoTiterPlate (PTP), while the other SG library was sequenced in two full PTP runs. One full PTP was used to sequence the PE library of *A. deanei*. Each library of *S. culicis* was sequenced in one full PTP run.

## Reference-guided assembly of A. deanei and S. culicis genomes

It was possible to predict 9,964 genes but only 2,591 were validated using *de novo* assembly strategy. The reference-guided assembly gave us an overview of the predicted proteome of both trypanosomatid genomes, with 7,899 valid genes predicted. Therefore, the genomes were assembled using the last strategy that improves the quality of genome assembly, minimizing the low coverage sequencing and the presence of repetitive DNA sequences. The reference sequences represent chromosomal DNA from a large percentage of parasitic genomes and create ambiguities for sequence alignment and assembly programs.

A total of 73,808 protein sequences were selected from the TryTRipDB (release 3.3 – <http://tritrypdb.org/common/downloads/>) . Sequences containing start codons different from ATG or containing stop codons in the middle of the sequence were filtered out. The selected sequences were compared to reads of *A. deanei* and *S. culicis* using tblastn, applying an E-value cut-off threshold of 1e−05 to define a set of significant reads to reconstruct each protein sequence. Each protein sequence was reconstructed with the counterpart set of reads selected using the software Newbler 2.6 according to the following parameters: -rip -r -urt -minlen 15 -ml 8.

For each contig obtained, the start and end points were identified by the EMBOSS program getorf. To confirm whether each predicted protein-coding gene corresponds to the reference protein sequence, the contig sequence was submitted to a tblastn similarity search against the respective protein reference sequence. An assembly was considered closed when the following conditions were fulfilled: (a) each hit has only one high-scoring segment pair (HSPs), (b) query and subject coverage is greater than or equal to 60% and (c) the hit has a 90% minimum coverage of an in-frame predicted protein-coding gene.

The assemblies that did not fulfill the above conditions were treated as follows: contig sequences of unclosed assemblies were submitted to a blastn similarity search against the reads that were not selected to reconstruct the protein sequence or those that were not completely assembled (including the unclosed and closed assemblies). In the assembly, the new set of reads selected for each protein reference sequence was joined to the previous set of reads used in the reconstruction of the corresponding reference protein sequence. The analysis of predicted protein-coding genes and assembly followed the conditions mentioned above, except in the assembly step where condition (a) was not applied.

For those reads that were not selected or were not completely assembled, a new assembly was performed using the software Newbler 2.6 with the same parameters cited above. The contigs from this new assembly were compared using blastn to the contigs from the closed assemblies to detect regions that did not align, indicating exclusive regions. Possible ORFs in those regions were predicted using the software Glimmer.

Microsatellites were identified in the genomes of the protozoan parasites and of their symbiotic bacteria using the Repeat Finder program (<http://gicab.decom.cefetmg.br:8080/bio-web/>). Repeats were defined as repeating sequences ranging from 1 to 20 repeating nucleotides, with up to 1 gap between repeat units, and up to 20% change in sequence compared with neighboring repeat units. Microsatellites formed by mono-, di-, tri-, tetra or more nucleotides should repeat at least 5, 4, 3 or 2 times, respectively.

## Bacterial endosymbiont genome assembly

To identify the contigs generated for *A. deanei* that correspond to its bacterial endosymbiont, the contigs obtained using our strategy were compared with four contigs of the *A. deanei* bacterial endosymbiont genome (kindly provided by Fiocruz, Paraná, Brazil). Scaffolds corresponding to the bacterial endosymbiont were identified and assembled. To close the gaps in the scaffolds, each assembled contig was inserted in the scaffold assembly using the program Consed. Three copies of ribosomal operons were inserted to close the final gaps between the scaffolds. The final assembly generated only one contig containing 821,813 bp. A region of approximately 15,000 bp was absent in the *A. deanei* bacterial endosymbiont genome used as reference.

The same strategy was used to obtain a putative assembly of the bacterial endosymbiont genome of *S. culicis,* as the endosymbiont was not isolated*.* The scaffolds generated for *S. culicis* that correspond to its endosymbiont were identified by comparison with the four contigs of the *A. deanei* bacterial endosymbiont genome. The scaffolds identified comprised 1.2 Mbp. To obtain only one contig, all gaps were closed and the three copies of the ribosomal operon were inserted. This contig comprises 823,673 bp. The remaining 380,000 bp were not used in the assembly. Experimental work would be required to validate this putative assembly of the bacterial endosymbiont genome of *S. culicis*.

## Automatic Functional Annotation

Automatic functional annotation of the *A. deanei* and *S. culicis* genomes was performed using the System for Automated Bacterial Integrated Annotation (SABIA) (Almeida et al., 2004) according to the following criteria:

- ORFs with blastP hits in the KEGG database and with a minimum 50% coverage of both the query and the subject sequence: the first ten hits were analyzed and the product was imported from KEGG ORTHOLOGY (KO) if one was associated with the hit, or from KEGG GENES definition if no KO was associated with the first ten hits.
- Remaining ORFs with blastP hits in NCBI-nr, UniProtKB/Swiss-Prot or TCDB databases and with a subject and query coverage ≥ 50% were assigned as valid or hypothetical, depending on the annotation imported from the database.
- ORFs with no blastP hits in the databases mentioned above and no InterPro results or ORFs that did not fit the criteria above were assigned as hypothetical.

The above criteria were also used for automatic functional annotation of bacterial endosymbiont genomes from both trypanosomatids. The similarity search parameters were modified as follows: minimum 60% coverage of both the query and subject sequence and minimum 60% positive for the *A. deanei* endosymbiont genome. A blastP similarity search was performed against KEGG with query and subject coverage ≥ 70% and ≥ 60% positive for the *S. culicis* endosymbiont genome, while against other databases the similarity search was performed with a query and subject coverage ≥ 60% and ≥ 60% positive. Some ORFs were manually annotated in both bacterial endosymbiont genomes.

## Genomic alignment and clustering analysis

Sequences were retrieved from TriTrypDB version 4.0 including *Leishmania braziliensis (*p=8,357 proteins), *L. infantum* (p=8,241), *L. major* (p=8,412), *L. mexicana* (p=8,250), *L. tarentolae* (p=8,452), *Trypanosoma brucei* (p=9,826), *T. congolense* (p=13,459), *T. cruzi* (p=23,311) and *T. vivax* (p=11,885). Sequences from *A. deanei* (p=17,324) and *S. culicis* (p=12,465) are from the present work. All sequences were compared against each other using BLAST with a 1x10^-30^ e-value threshold and clustered with the MCL algorithm using a 1.2 inflation value, providing a highly agglomerative solution. We also included ORFs from *Crithidia fasciculata* that are available at TriTrypDB (225,991 ORFs), which were compared to the previously obtained clusters.

A phylogenomic approach was also applied in order to establish the evolutionary relationship among *Achromobacter xylosoxidans A8*, *Bordetella petrii DSM 12804*, *Taylorella asinigenitalis MCE3*, *Taylorella equigenitalis MCE9*, *Candidatus* Kinetoplastibacterium blastocrithidii and *Candidatus* Kinetoplastibacterium crithidii. *Pseudomonas aeruginosa* PA7, which is a Gammaproteobacteria, was used as outgroup. The first step of the phylogenomic analysis was the identification of orthologs through bidirectional best hit (BBH) and the exclusion of paralog clusters, which are defined as a gene set where every gene is a BBH. Multi-FASTA putative ortholog files were used as input for multiple alignments using CLUSTALw algorithm [32] with default parameters.

The gene concatenation of 235 alignments was performed using SCaFos software . Phylogenies involving seven concatenated deduced amino acid sequences were estimated by NJ and maximum parsimony (MP) , both available in the Molecular Evolutionary Genetics Analysis (MEGA) program version 5.05 . The evolutionary distances were computed using the p-distance and the Poisson-corrected amino acids distance. The datasets contained 80,062 and 87,004 positions for the complete and pairwise deletion of gaps or missing data, respectively. The bootstrap test of phylogeny was performed using 1,500 repetitions. The MP tree was obtained using the close-neighbor-interchange algorithm with search level 3 in which the initial trees were obtained by random addition of sequences (10 replicates). The complete deletion, partial deletion, and all sites included were tested. The bootstrap test was implemented using 1,500 replicates.

## Endosymbiont genome analysis

The genomic alignments were performed with the Artemis Comparison Tool (ACT) .

A blastp analysis was performed to compare the genomes of the *A. deanei* and *S. culicis* endosymbionts, *T. equigenitalis* MCE9, *T. asinigenitalis* MCE3, *B. petrii* DSM 12804, and *A. xylosoxidans* A8, including its two plasmids (pA81 and pA82). In this analysis, genes with bidirectional best reads were grouped together as a cluster. The minimum criteria for inclusion in a cluster were 70% coverage, 70% positive similarity and an e-value of at least 1e-5.

# References

1. Figueiredo LM, Cross GA, Janzen CJ (2009) Epigenetic regulation in African trypanosomes: a new kid on the block. Nature Reviews Microbiology 7: 504-513.

2. Glover L, Horn D (2012) Trypanosomal histone gammaH2A and the DNA damage response. Molecular and Biochemical Parasitology 183: 78-83.

3. Alsford S, Kawahara T, Isamah C, Horn D (2007) A sirtuin in the African trypanosome is involved in both DNA repair and telomeric gene silencing but is not required for antigenic variation. Molecular Microbiology 63: 724-736.

4. Carafa V, Nebbioso A, Altucci L (2012) Sirtuins and disease: the road ahead. Frontiers in pharmacology 3: 4.

5. Ingram AK, Horn D (2002) Histone deacetylases in T*rypanosoma brucei:* two are essential and another is required for normal cell cycle progression. Molecular Microbiology 45: 89-97.

6. Nguyen AT, Zhang Y (2011) The diverse functions of Dot1 and H3K79 methylation. Genes and Development 25: 1345-1358.

7. Qian C, Zhou MM (2006) SET domain protein lysine methyltransferases: Structure, specificity and catalysis. Cellular and molecular life sciences : CMLS 63: 2755-2763.

8. Park YJ, Luger K (2008) Histone chaperones in nucleosome eviction and histone exchange. Current Opinion in Structural Biology 18: 282-289.

9. Mujtaba S, Zeng L, Zhou MM (2007) Structure and acetyl-lysine recognition of the bromodomain. Oncogene 26: 5521-5527.

10. Liu B, Liu Y, Motyka SA, Agbo EE, Englund PT (2005) Fellowship of the rings: the replication of kinetoplast DNA. Trends Parasitol 21: 363-369.

11. Das A, Dasgupta A, Sengupta T, Majumder HK (2004) Topoisomerases of kinetoplastid parasites as potential chemotherapeutic targets. Trends in Parasitology 20: 381-387.

12. Liu B, Wang J, Yaffe N, Lindsay ME, Zhao Z, et al. (2009) Trypanosomes have six mitochondrial DNA helicases with one controlling kinetoplast maxicircle replication. Molecular Cell 35: 490-501.

13. Schamber-Reis BL, Nardelli S, Regis-Silva CG, Campos PC, Cerqueira PG, et al. (2012) DNA polymerase beta from *Trypanosoma cruzi* is involved in kinetoplast DNA replication and repair of oxidative lesions. Molecular and Biochemical Parasitology 183: 122-131.

14. Mendez J, Stillman B (2003) Perpetuating the double helix: molecular machines at eukaryotic DNA replication origins. BioEssays 25: 1158-1167.

15. Godoy PD, Nogueira-Junior LA, Paes LS, Cornejo A, Martins RM, et al. (2009) Trypanosome prereplication machinery contains a single functional orc1/cdc6 protein, which is typical of archaea. Eukaryotic Cell 8: 1592-1603.

16. Dang HQ, Li Z (2011) The Cdc45.Mcm2-7.GINS protein complex in trypanosomes regulates DNA replication and interacts with two Orc1-like proteins in the origin recognition complex. The Journal of Biological Chemistry 286: 32424-32435.

17. Veiga-Santos P, Barrias ES, Santos JF, de Barros Moreira TL, de Carvalho TM, et al. (2012) Effects of amiodarone and posaconazole on the growth and ultrastructure of *Trypanosoma cruzi*. International Journal of Antimicrobial Agents 40: 61-71.

18. Chia N, Cann I, Olsen GJ (2010) Evolution of DNA replication protein complexes in eukaryotes and Archaea. PLoS One 5: e10866.

19. Akita M, Adachi A, Takemura K, Yamagami T, Matsunaga F, et al. (2010) Cdc6/Orc1 from *Pyrococcus furiosus* may act as the origin recognition protein and Mcm helicase recruiter. Genes to cells 15: 537-552.

20. Ilves I, Petojevic T, Pesavento JJ, Botchan MR (2010) Activation of the MCM2-7 helicase by association with Cdc45 and GINS proteins. Molecular Cell 37: 247-258.

21. Maga G, Hubscher U (2003) Proliferating cell nuclear antigen (PCNA): a dancer with many partners. Journal of Cell Science 116: 3051-3060.

22. Oakley GG, Patrick SM (2010) Replication protein A: directing traffic at the intersection of replication and repair. Frontiers in bioscience 15: 883-900.

23. Odegrip R, Schoen S, Haggard-Ljungquist E, Park K, Chattoraj DK (2000) The interaction of bacteriophage P2 B protein with *Escherichia coli* DnaB helicase. The Journal of Virology 74: 4057-4063.

24. El-Sayed NM, Myler PJ, Blandin G, Berriman M, Crabtree J, et al. (2005) Comparative genomics of trypanosomatid parasitic protozoa. Science 309: 404-409.

25. Burton P, McBride DJ, Wilkes JM, Barry JD, McCulloch R (2007) Ku heterodimer-independent end joining in *Trypanosoma brucei* cell extracts relies upon sequence microhomology. Eukaryotic Cell 6: 1773-1781.

26. Van Houten B, Gamper H, Hearst JE, Sancar A (1988) Analysis of sequential steps of nucleotide excision repair in *Escherichia coli* using synthetic substrates containing single psoralen adducts. The Journal of Biological Chemistry 263: 16553-16560.

27. Fonville NC, Vaksman Z, DeNapoli J, Hastings PJ, Rosenberg SM (2011) Pathways of resistance to thymineless death in Escherichia coli and the function of UvrD. Genetics 189: 23-36.

28. Ivens AC, Peacock CS, Worthey EA, Murphy L, Aggarwal G, et al. (2005) The genome of the kinetoplastid parasite, *Leishmania major*. Science 309: 436-442.

29. Novak E, Haapalainen EF, Da Silva S, Da Silveira JF (1988) Protein Synthesis in Isolated Symbionts from the Flagellate Protozoon *Crithidia deanei*. Journal of Eukaryotic Microbiology 35: 375-378.

30. Aslett M, Aurrecoechea C, Berriman M, Brestelli J, Brunk BP, et al. (2010) TriTrypDB: a functional genomic resource for the Trypanosomatidae. Nucleic Acids Research 38: D457-D462.

31. Van Dongen S (2000) Graph Clustering by Flow Simulation. Utrecht: Universisty of Utrecht.

32. Larkin MA, Blackshields G, Brown NP, Chenna R, McGettigan PA, et al. (2007) Clustal W and Clustal X version 2.0. Bioinformatics 23: 2947-2948.

33. Roure B, Rodriguez-Ezpeleta N, Philippe H (2007) SCaFoS: a tool for selection, concatenation and fusion of sequences for phylogenomics. BMC evolutionary biology 7 Suppl 1: S2.

34. Saitou N, Nei M (1987) The neighbor-joining method: A new method for reconstructing phylogenetic trees. Molecular Biology and Evolution 4: 406-425.

35. Lake JA (1987) A rate-independent technique for analysis of nucleic acid sequences: evolutionary parsimony. Molecular Biology and Evolution 4: 167-191.

36. Kumar S, Tamura K, Jakobsen IB, Nei M (2001) MEGA2: molecular evolutionary genetics analysis software. Bioinformatics 17: 1244-1245.

37. Kumar S, Nei M, Dudley J, Tamura K (2008) MEGA: a biologist-centric software for evolutionary analysis of DNA and protein sequences. Briefings in bioinformatics 9: 299-306.

38. Carver TJ, Rutherford KM, Berriman M, Rajandream MA, Barrell BG, et al. (2005) ACT: the Artemis Comparison Tool. Bioinformatics 21: 3422-3423.
